# STX17 dynamically regulated by Fis1 induces mitophagy via hierarchical macroautophagic mechanism

**DOI:** 10.1038/s41467-019-10096-1

**Published:** 2019-05-03

**Authors:** Hongxu Xian, Qiaoyun Yang, Lin Xiao, Han-Ming Shen, Yih-Cherng Liou

**Affiliations:** 1 Department of Biological Sciences, Faculty of Science, National University of Singapore, 14 Science Drive 4, 117543 Singapore, Singapore; 2 Department of Physiology, Yong Loo Lin School of Medicine, National University of Singapore, Singapore, 117597 Singapore; 3 NUS Graduate School for Integrative Sciences and Engineering, National University of Singapore, Singapore, Singapore

**Keywords:** Mitophagy, SNARE, Mitochondria

## Abstract

Mitophagy is the selective autophagic targeting and removal of dysfunctional mitochondria. While PINK1/Parkin-dependent mitophagy is well-characterized, PINK1/Parkin-independent route is poorly understood. Using structure illumination microscopy (SR-SIM), we demonstrate that the SNARE protein Syntaxin 17 (STX17) initiates mitophagy upon depletion of outer mitochondrial membrane protein Fis1. With proteomics analysis, we identify the STX17-Fis1 interaction, which controls the dynamic shuffling of STX17 between ER and mitochondria. Fis1 loss results in aberrant STX17 accumulation on mitochondria, which exposes the N terminus and promotes self-oligomerization to trigger mitophagy. Mitochondrial STX17 interacts with ATG14 and recruits core autophagy proteins to form mitophagosome, followed by Rab7-dependent mitophagosome-lysosome fusion. Furthermore, Fis1 loss impairs mitochondrial respiration and potentially sensitizes cells to mitochondrial clearance, which is mediated through canonical autophagy machinery, closely linking non-selective macroautophagy to mitochondrial turnover. Our findings uncover a PINK1/Parkin-independent mitophagic mechanism in which outer mitochondrial membrane protein Fis1 regulates mitochondrial quality control.

## Abstract (web summary)

Mitophagy plays a critical role in cellular homeostasis, and PINK1/Parkin-mediated mitophagy is the most thoroughly characterized. Here, Xian et al. show that STX17 induces mitophagy via a macroautophagy pathway regulated by Fis1, by a PINK1/Parkin-independent route.

## Introduction

Mitochondria are subcellular organelles that integrate cellular metabolism and cell fate. Dysregulated mitochondrial activity in oxidative phosphorylation leads to the overproduction of reactive oxidative species (ROS), which damage cellular DNA and proteins^1,2^. Thus, the quality control of mitochondria is crucial for cellular functions. Removal of damaged mitochondria is mediated by autophagy^3,4^. Autophagy is an evolutionarily conserved and programmed process through which damaged or unwanted cellular proteins and organelles are degraded^5,6^. Autophagic elimination of mitochondria occurs via a selective autophagic process known as mitophagy, whereby the isolation membranes enrich on the mitochondria and emerge as double-membrane structures, called as autophagosomes. Mature autophagosome fuses with lysosome, followed by the subsequent degradation of mitochondria^7–10^. Of note, dysregulation of mitophagy is particularly implicated in aging, neurodegenerative diseases, cancer, and cellular development^11–14^.

Several mechanisms have been reported to account for mitophagy^4,15,16^. The best-characterized route of mitophagy is the PINK1/Parkin-mediated pathway, whereby PINK1 is stabilized on the outer membrane of damaged mitochondria in response to mitochondrial depolarization. PINK1 recruits cytosolic Parkin, an E3 ligase, onto mitochondria, leading to a feed-forward amplification loop of the ubiquitination of outer mitochondrial membrane (OMM) proteins, and priming of mitophagy by the accumulation of P62 and LC3 on mitochondria^17–24^. Moreover, PINK1/Parkin-independent pathways of mitochondrial elimination involving mitophagy receptors, including Nix (BNIP3L, BCL2/adenovirus E1B 19 kDa protein-interacting protein 3-like), FUNDC1 (FUN14 domain-containing protein 1), Bcl-2-L-13 (Bcl-2-like protein 13), and FKBP8 (peptidyl-prolyl *cis*–*trans* isomerase FKBP8) have been reported^14,25–30^. However, it remains unclear whether additional mechanisms of PINK1/Parkin-independent mitophagy could exist in fetal tissues or cell lines, which show no or low endogenous Parkin expression^31,32^.

Mitochondrial fission protein 1 (Fis1) is a 16 kDa OMM protein, with a single transmembrane domain integrating mitochondrial outer membrane at its C terminus, and two tetratricopeptide repeat (TPR) motifs facing cytosol. Fis1 was first identified in budding yeast *Saccharomyces cerevisiae* to physically interact with Dnm1 (the yeast ortholog of Drp1), mediating the assembly of GTPase protein Dnm1 to promote mitochondrial division^33^. However, the role of Fis1 in mitochondrial dynamics of mammals has become controversial with the discovery that loss of Fis1 fails to alleviate Drp1 recruitment and prevent mitochondrial fission, given by the conditional knockout of Fis1 in human colon carcinoma cells^34^, although the overexpression of Fis1 promotes mitochondrial fission^35,36^. Additionally, more Drp1 receptors, including mitochondrial fission factor (Mff), mitochondrial dynamics proteins of 49 and 51 kDa (MiD49 and MiD51), are shown to be essential for the recruitment of Drp1 onto the mitochondria^34,37–41^. In contrast, human Fis1 was debated whether it is indispensable for mitochondrial fragmentation. Hence, the bona fide role of mitochondrial Fis1 remains unknown.

Syntaxin 17 (STX17) is an ER-resident SNARE (soluble *N*-ethylmaleimide-sensitive factor attachment protein receptor) protein involved in autophagy. Different roles of STX17 have been proposed concerning its mechanisms of action. Studies have shown that STX17 is recruited to the outer membrane of mature autophagosome, to interact with cytosolic SNAP29 and lysosomal VAMP8 to facilitate autophagosome–lysosome fusion^42–44^. Paradoxically, another study has showed that upon starvation-induced autophagy, STX17 translocalizes to ER–mitochondria contact site, whereby STX17 binds and recruits ATG14 to initiate the formation of phagophore, supporting a positive role of STX17 in the early step of autophagy^45^. In addition, STX17 has been reported to regulate mitochondrial dynamics by regulating the localization and GTPase activity of Drp1, integrating mitochondrial dynamics and autophagy, in response to nutrient conditions^46^. Given the novel and impactful roles of STX17 on autophagy and mitochondrial morphology, it would be interesting to study whether STX17 contributes to mitochondrial autophagy.

In the present study, we demonstrate that the TPR2 domain of Fis1 interacts with STX17, preventing the exposure of N terminus of STX17 for self-oligomerization and restraining its entry onto the mitochondria. Strikingly, STX17 overexpression induces mitophagy upon Fis1 depletion in a PINK1/Parkin-independent pathway. Here we uncover that loss of Fis1 promotes the relocalization of STX17 onto ER–mitochondria contact sites and mitochondria, whereby it recruits ATG14 on the mitochondria for canonical autophagy biogenesis, with the mitochondria as cargoes. Further recruitments of downstream ATG (autophagy-related) proteins, including ATG5 and ATG16, incorporate mitochondria into mature mitophagosome for turnover. Therefore, our study reveals that STX17 and Fis1 autonomously regulate mitophagy, which occurs in the absence of ectopic toxins as mitochondrial damage, implying the fine-tune regulation of mitochondrial quality control.

## Results

### Fis1 binds STX17 biochemically

**Fig. 1:**
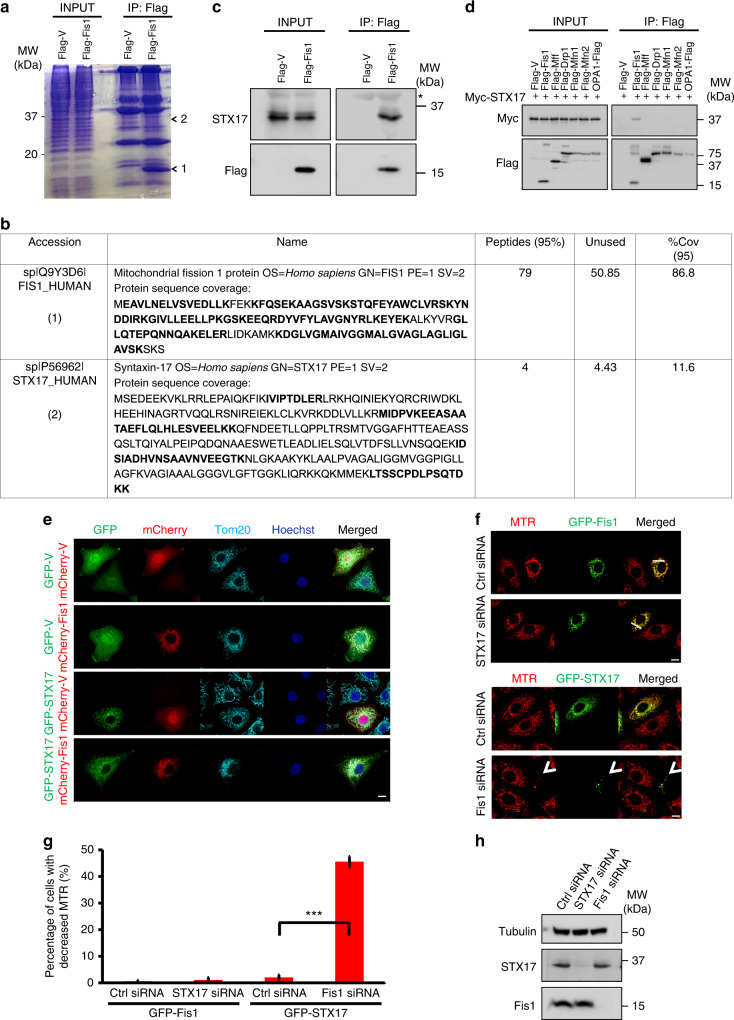
Mitochondrial fission 1 protein (Fis1) and syntaxin 17 (STX17) interact and partially colocalize. **a**, **b** HeLa cells were transfected with Flag-tagged vector or Fis1. After 24 h, cells were collected for immunoprecipitation (IP) with anti-Flag beads. Coomassie blue staining was used to visualize bands 1 and 2 (**a**). Results for mass spectrometry analysis of band 1 and 2 are summarized (**b**). **c** Cells treated as in **a** were extracted. Anti-Flag immunoprecipitates were separated by sodium dodecyl sulfate-polyacrylamide gel electrophoresis (SDS-PAGE) and immunoblotted for STX17 and Flag. Asterisk indicates a non-specific band. **d** HEK293T cells were co-transfected with Myc-tagged STX17 and Flag-tagged plasmids as indicated. Cells were solubilized for IP with anti-Flag and analyzed with Myc and Flag antibodies respectively. **e** HeLa cells were transfected with green fluorescent protein (GFP)-tagged vector or STX17 (green) and mCherry-tagged vector or plasmid encoding Fis1 (red) for 24 h. Cells were fixed and stained with anti-Tom20 (cyan). Hoechst, blue. Scale bar, 10 µm. **f** HeLa cells were treated with the indicated small interfering RNA (siRNA) for 24 h before transfecting with GFP-tagged Fis1 (green) or GFP-STX17 (green) for further 24 h. Representative confocal images of live cells are shown. Mitochondrial morphology was visualized using MitoTracker Red (MTR, red). Scale bar, 10 µm. White arrowhead indicates cells with decreased MTR. **g** Quantification of cells with decreased MTR as shown in **f**. Error bars, SD. ***P < 0.001 (two-tailed unpaired Student’s *t* test, *n* = 150 cells from three independent experiments). **h** STX17 and Fis1 silencing was verified in HeLa cells by immunoblotting

Given the uncertain role of mitochondrial Fis1, we first sought to identify proteins interacting with mitochondrial Fis1 in mammalian cells. We performed pull-down assay with Flag-tagged Fis1-expressing HeLa cells, followed by mass spectrometry analysis of bands of interest (Figs. 1a bands 1 and 2). Interestingly, we identified the SNARE protein STX17 as a putative binding partner and confirmed the interaction by co-immunoprecipitation (Fig. 1b, c and Supplementary Fig. 1a, b). Moreover, STX17 was found to interact specifically with Fis1, but not with other proteins involved in mitochondrial dynamics, including Mff, Drp1, Mfn1, Mfn2, and OPA1, suggesting a selective association between Fis1 and STX17 (Fig. 1d). To corroborate this interaction, we examined the localization of mCherry-tagged Fis1 and green fluorescent protein (GFP)-tagged STX17 by immunofluorescence analysis in HeLa cells, due to the incapability of commercial antibody to analyze endogenous STX17 (Fig. 1e and Supplementary Fig. 1c). Line-scan analysis confirmed the partial colocalization of STX17 and Fis1. Then, we attempted to investigate the role of STX17 on Fis1 localization or vice versa. Upon *STX17* knockdown, Fis1 remained on the mitochondria, which are indicated by MitoTracker (MTR, Fig. 1f and Supplementary Fig. 1d). However, in Fis1-deficient cells, GFP-STX17 formed punctate structures and 45.5 ± 2.0% of GFP-STX17-positive cells possessed markedly abrogated MTR signal (Fig. 1f–h).

**Fig. 2:**
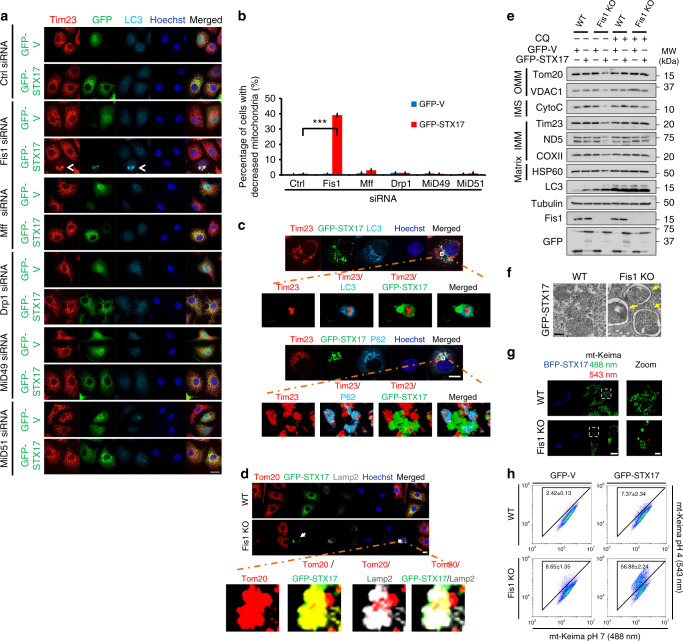
Syntaxin 17 (STX17) initiates mitophagy upon mitochondrial fission 1 protein (Fis1) depletion. **a** HeLa cells were transfected with respective small interfering RNAs (siRNAs) for 24 h, before transient transfection with green fluorescent protein (GFP)-tagged vector or STX17 (green) for further 24 h. Cells were fixed and immunostained with Tim23 (red) and LC3 (cyan) antibodies. Hoechst, blue. Scale bar, 10 µm. White arrowhead indicates mitophagic cell. **b** Quantification of cells with decreased mitochondria as shown in **a**. Data are mean ± SD (*n* = 150 cells from three independent experiments). ***P < 0.001 (two-tailed unpaired Student’s *t* test). **c** Fis1 knockout (KO) HeLa cells were transfected with GFP-tagged STX17 for 24 h. Cells were fixed and analyzed by immunofluorescence against Tim23 (red) and LC3 or P62 (cyan). Z-stack images were collected and a representative three-dimensional reconstruction example is shown. Hoechst, blue. Scale bar, 10 µm. **d** Wild-type (*WT*) or *Fis1 KO* HeLa cells were transiently transfected with GFP-tagged STX17 (green) for 24 h. Images were acquired by super-resolution structured illumination microscopy (SR-SIM) after staining for Tom20 (red) and Lamp2 (gray). Hoechst, blue. Scale bar, 10 µm. Enlarged image represents in three-dimensional reconstruction. White arrow indicates the signal of GFP-STX17. **e** *WT* or *Fis1 KO* HeLa cells were transiently transfected with GFP-tagged vector or STX17 for 6 h. Cells were cultured with or without chloroquine (CQ) for further 66 h. Cell lysates were immunoblotted for outer mitochondrial membrane (OMM), intermembrane space (IMS), inner membrane mitochondrial (IMM), and matrix proteins. **f** *WT* or *Fis1 KO* HeLa cells transiently expressing GFP-tagged STX17 were analyzed by conventional transmission electron microscopy. Scale bars, 0.2 µm. Yellow arrows indicate autophagic structures enclosing mitochondria. **g** *WT* or *Fis1 KO* HeLa cells stably expressing mitochondrial-targeted form of the fluorescent reporter Keima (mt-Keima) were transiently transfected with blue fluorescent protein (BFP)-tagged STX17 (blue). Live cells were visualized by confocal microscopy excited with 488 nm (green) and 543 nm (red). Scale bar, 10 μm. **h** *WT* or *Fis1 KO* HeLa cells stably expressing mt-Keima were transiently transfected with GFP-tagged vector or STX17 for 48 h. mt-Keima fluorescence in GFP-positive cells was measured using flow cytometry by excitation at 488 nm (neutral) and 543 nm (acidic). Data are mean ± SD (*n* = 3)

### STX17 initiates mitophagy upon Fis1 depletion

The loss of mitochondrial signal in Fis1-depleted cells (Fig. 1f–h) led us to address the question whether STX17 might induce mitophagy in the absence of Fis1. To this end, knocking down of *Fis1* in HeLa cells transiently expressing GFP-tagged STX17 induced a robust decrease of mitochondrial inner membrane protein Tim23, which colocalized with autophagosome marker LC3 (Fig. 2a, b and Supplementary Fig. 2a). Congruently, Fis1 silencing essentially reached the same observation in 41.8 ± 1.0% of HeLa cells stably expressing GFP-tagged STX17 (Supplementary Fig. 2b, c). To further investigate the role of Fis1, we then generated *Fis1 knockout* (*KO*) HeLa cells using CRISPR/Cas9 (Supplementary Fig. 1e, f). Interestingly, when STX17 was introduced into *Fis1 KO* cells, fragmented mitochondria were enclosed within autophagosome (LC3), autophagy receptor (P62), and lysosome (Lamp2), observed by three-dimensional reconstructed images (Fig. 2c, d, Supplementary Fig. 2e, f, and Supplementary Movie 1). Meanwhile, STX17 was found to occasionally colocalized with the mitochondria (MTR or Tom20) (Supplementary Fig. 1g). Furthermore, STX17 induced a significant decrease of overall mitochondrial proteins in *Fis1 KO* cells detected by immunoblotting, and lysosomal inhibitor chloroquine (CQ) substantially rescued the turnover of mitochondrial proteins, suggesting that the reduction of overall mitochondrial protein levels stems from mitophagy (Fig. 2e). Strikingly, the degradation of mitochondrial overall proteins induced by *Fis1* siRNA interference in HeLa cells stably expressing GFP-STX17 complemented this notion (Supplementary Fig. 2d).

Electron microscopy (EM) of STX17-transfected *Fis1 KO* cells revealed a significantly increased number of mitochondria within autophagosome (Fig. 2f and Supplementary Fig. 3a, b). Total number of mitochondria was markedly decreased upon STX17 overexpression of Fis1-depleted cells (Supplementary Fig. 3c). To further validate mitophagy, we utilized ratiometric measurement applied with mitochondrial-targeted form of the fluorescent reporter Keima (mt-Keima). Mitochondrial matrix-targeted mt-Keima shifts its fluorescence excitation, and labels mitochondria in acidic lysosome excited at 543 nm, due to pH-sensitive properties of Keima protein (Fig. 2g). Analyzed from the ratio of acidified mt-Keima per cell by flow cytometry, STX17 induction in Fis1-depleted cells was observed to increase acidic mt-Keima proportion from 2.42 ± 0.13 to 56.88 ± 2.24% (Fig. 2h and Supplementary Fig. 3d), confirming undergoing mitophagy. Additionally, by pulsing cells with mitochondrial membrane potential-dependent dye, TMRM (tetramethylrhodamine, methyl ester), mitochondrial membrane potential was decreased by 18 ± 2% at 24 h and 45 ± 4% at 48 h, respectively, in *Fis1 KO* cells, upon GFP-tagged STX17 transfection, indicative of impaired integrity of mitochondria (Supplementary Fig. 3e, f). Collectively, our results imply that STX17 overexpression induces mitophagy upon Fis1 depletion in HeLa cells.

### STX17-induced mitophagy is PINK1/Parkin-independent

**Fig. 3:**
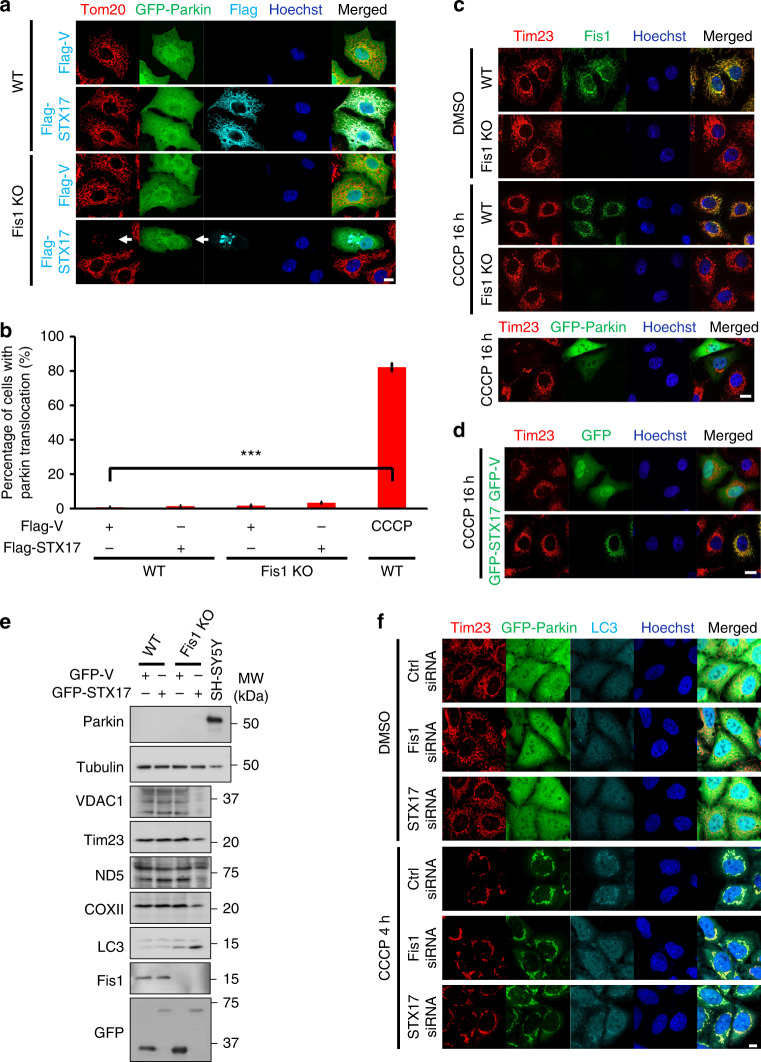
Syntaxin 17 (STX17)-induced mitophagy is PINK/Parkin-independent. **a** Wild-type (*WT*) or mitochondrial fission 1 protein knockout (*Fis1 KO*) HeLa cells were transfected with Flag-tagged vector or STX17 and green fluorescent protein (GFP)-tagged Parkin (green) for 24 h. Cells were analyzed by immunofluorescence microscopy using antibodies against Tom20 (red) and Flag (cyan). Hoechst, blue. Scale bar, 10 µm. White arrow indicates cell with STX17 overexpression-mediated mitophagy. **b** Bar chart shows the percentage of cells with Parkin translocation as indicated in **a**. Parkin translocation upon carbonyl cyanide 3-chlorophenylhydrazone (CCCP) treatment at 10 µM for 4 h was quantified as positive control. Data are mean ± SD (*n* = 150 cells from three independent experiments). ***P < 0.001 (two-tailed unpaired Student’s *t* test). **c ***WT* or *Fis1 KO* HeLa cells were incubated with dimethyl sulfoxide (DMSO) or 10 µM CCCP for 16 h and then fixed and immunostained for Tim23 (red) and Fis1 (green). Cells transiently expressing GFP-tagged Parkin and treated with 10 µM CCCP for 16 h were used as a positive control for mitophagy. Hoechst, blue. Scale bar, 10 µm. **d** HeLa cells expressing GFP-tagged vector or STX17 were treated with 10 µM CCCP for 16 h and immunostained for Tim23 (red). Hoechst, blue. Scale bar, 10 µm. **e** *WT* or *Fis1 KO* HeLa cells were transfected with GFP-tagged vector or STX17 for 72 h. SH-SY5Y cell lysate was used as the positive control for the expression of endogenous Parkin. Cell lysates were immunoblotted for Parkin and mitochondrial proteins. **f** HeLa cells stably expressing GFP-Parkin (green) were transfected with small interfering RNA (siRNA) against control, *Fis1* or *STX17* for 48 h. Then, cells were treated with or without 10 µM CCCP for further 4 h. Cells were visualized by immunostaining for Tim23 (red) and LC3 (cyan). Hoechst, blue. Scale bar, 10 µm

Mammalian PINK1 and Parkin have been described to play important roles in mitophagy^19,21,22,24^. However, the impact of PINK1 and Parkin on mitophagy relies on ectopic toxicity such as mitochondrial depolarization^22,24^. In addition, evidence supports that HeLa cells express undetectable levels of endogenous Parkin, and PINK1/Parkin-dependent mitophagy in HeLa cells requires the introduction of exogenous Parkin^31^. Thus, it is reasonably assumed that STX17-induced mitophagy in *Fis1 KO* cells is uncoupled to PINK1/Parkin-mediated pathway. To address this, we expressed GFP-Parkin in *WT* or Fis1-depleted cells, with or without Flag-STX17, to monitor mitochondrial translocation of Parkin (Fig. 3a, b). As expected, translocation of GFP-Parkin was not induced by Fis1 loss, overexpression of STX17, or even during STX17 overexpression-mediated mitophagy of Fis1-depleted cells. Furthermore, carbonyl cyanide 3-chlorophenylhydrazone (CCCP) treatment of Fis1-depleted or STX17-overexpressing cells was unable to induce mitophagy (Fig. 3c, d). Notably, Parkin was not expressed even when STX17 initiated mitophagy upon Fis1 loss, albeit substantial turnover of overall mitochondrial proteins was confirmed (Fig. 3e). CCCP-induced translocation of Parkin was unaltered by siRNA silencing of *Fis1* or *STX17* under 10 µM CCCP treatment for 4 h (Fig. 3f and Supplementary Fig. 4a), suggesting that STX17-induced mitophagy upon Fis1 depletion is uncoupled to PINK1/Parkin.

The notion that Fis1 and TBC1D15 (TBC1 domain family member 15) block mitophagy has been proposed for PINK1/Parkin-dependent mitophagy^47,48^. To this end, we assessed mitophagy initiation by knocking down *TBC1D15*, compared with Fis1 deficiency. Loss of TBC1D15 had no impact on mitophagy formation (Supplementary Fig. 4b–d). Correspondingly, we observed no interaction of STX17 and TBC1D15 (Supplementary Fig. 4e), revealing that TBC1D15, unlike Fis1, is not involved in STX17 overexpression-mediated mitophagy. Interestingly, this also further highlights the selective regulation between Fis1 and STX17 on PINK1/Parkin-independent mitophagy.

Alternatively, ubiquitination of mitochondrial proteins is significantly induced following Parkin translocation^18,23^. Hereby we analyzed mitochondrial ubiquitin (Ub) accumulation in STX17-induced mitophagy of Fis1-depleted cells by immunofluorescence. Gross enrichment of mitochondrial Ub was not apparent in STX17-induced mitophagy of *Fis1 KO* cells, although we did observe small amount of mitochondrial Ub in cells, possibly mediated by P62, which contains the UBA (Ub-associated) domain to recruit Ub (Supplementary Fig. 4f). Together, our results provide strong evidence that in *Fis1 KO* HeLa cells, STX17 initiates mitophagy through a PINK1/Parkin-independent route.

### The functional domains of STX17 and Fis1 for mitophagy

**Fig. 4:**
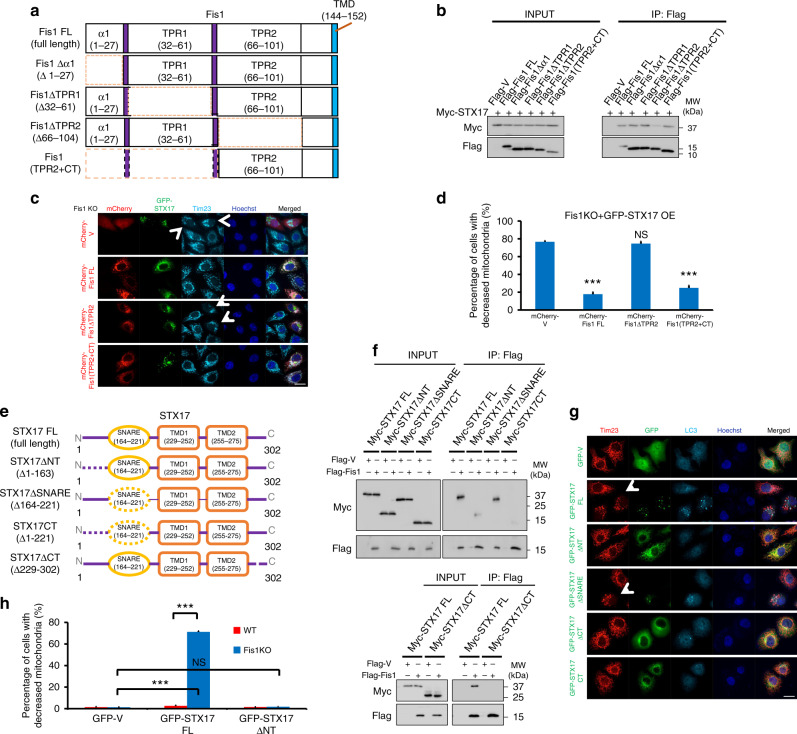
The functional domains of mitochondrial fission 1 protein (Fis1) and syntaxin 17 (STX17) for mitophagy. **a** Schematic diagram of Fis1 truncations. The deleted regions are represented by dotted lines. **b** HEK293T Cells were transiently co-transfected with the indicated plasmids for 24 h. Cells were lysed and immunoprecipitated with anti-Flag antibody. Co-immunoprecipitated Myc-tagged STX17 was detected by immunoblotting. **c** *Fis1 KO* HeLa cells were co-transfected with green fluorescent protein (GFP)-tagged STX17 (green) and plasmids encoding mCherry-Fis1 truncations (red). Cells were fixed, stained with Tim23 antibody (cyan), and then imaged for fluorescence microscopy. Hoechst, blue. Scale bar, 10 µm. Arrowheads indicate mitophagic cells. **d** Quantification of **c**. ***P < 0.001, NS, not significant (two-tailed unpaired Student’s *t* test, *n* = 150 cells from three independent experiments). **e** Schematic representation of STX17 domain mutants. Dotted regions represent deleted domains. **f** HEK293T cells were co-transfected with the indicated plasmids. After 24 h, cells were extracted and co-immunoprecipitated with anti-Flag beads, followed by immunoblotting with anti-Myc and anti-Flag antibodies, respectively. **g** *Fis1 KO* cells were transfected with GFP-tagged vector or the indicated STX17 truncations for 24 h. Cells were imaged following immunostaining for Tim23 (red) and LC3 (cyan). Hoechst, blue. Scale bar, 10 µm. Arrowheads indicate mitophagic cells. **h** Quantitative analysis of mitophagy induced by STX17 full-length (FL) or truncations in *Fis1 KO* cells. ***P < 0.001, NS, not significant (two-tailed unpaired Student’s *t* test, *n* = 150 cells from three independent experiments). Data are mean ± SD (**d**, **h**)

To delineate the functional domain of Fis1, which is responsible for the interaction between Fis1 and STX17, we assembled deletion constructs of Fis1 (Fig. 4a). We transiently expressed Flag-tagged Fis1 deletion constructs in HeLa cells and assessed the association of Fis1 truncations with STX17 by co-immunoprecipitation (Fig. 4b). Deletion of the TPR2 domain significantly decreased STX17 binding, whereas the construct containing TPR2 and C-terminal transmembrane domain (Flag-Fis1(TPR2 + CT)) was able to bind STX17, although both would affect the fission activity of Fis1 (Supplementary Fig. 5a, b), which is supported by previous finding^49^. We then addressed whether the TPR2 domain of Fis1 is critical for the regulation of mitophagy. The expression of Fis1 full-length (FL) or Flag-(TPR2 + CT) successfully reverted STX17-induced mitophagy from 76.6 ± 1.2 to 17.7 ± 2.3% or 24.7 ± 2.7%, respectively, in Fis1-depleted cells (Fig. 4c, d), indicating that the TPR2 domain of Fis1 is vital to negatively regulate STX17-induced mitophagy.

We then mapped the corresponding interaction domain of STX17 and determined that the N-terminal domain is required for Fis1 binding, although the transmembrane domain (CT) of STX17 also accounts for this association (Fig. 4e, f). Additionally, the N terminus deletion fragment of STX17 (STX17ΔNT) failed to induce mitophagy in *Fis1 KO* cells (Fig. 4g, h), substantiating that the interaction domain of STX17 with Fis1 is pivotal for the positive regulation of mitophagy. Interestingly, we observed that STX17 formed punctate structures in *Fis1 KO* cells during mitophagy (Figs. 2a, d and 4c, g), leading us to surmise that the formation of STX17 puncta is dependent on its N terminus, which in turn contributes to mitophagy initiation. To address this question, we examined the domain functions of STX17 and found that transfection of STX17 absent in its N-terminal domain (STX17ΔNT and STX17 CT) into *Fis1 KO* cells substantially abrogated puncta formation (Supplementary Fig. 5c, d) and self-oligomerization (Supplementary Fig. 5e). However, somewhat surprisingly, deletion of the STX17 SNARE domain (STX17ΔSNARE) could still induce mitophagy in *Fis1 KO* cells (Fig. 4 g). The SNARE domain is well known to function in autophagosome–lysosome fusion^42^. To reconcile this issue, we generated *STX17*-knockdown cells and re-introduced siRNA-resistant STX17ΔSNARE (Supplementary Fig. 5f–h). Even under conditions of Fis1 depletion, STX17ΔSNARE overexpression was unable to induce mitophagy when endogenous STX17 was silenced, suggesting that the endogenous STX17 is crucial for autophagosome–lysosomal fusion (the late stage of autophagy), whereas STX17 functioning in mitophagy induction (the early stage of mitophagy) could be in separate subcellular fractions and rely on distinct expression level. These findings essentially reach the conclusion that the interaction of the N terminus of STX17 and the TPR2 domain of Fis1 are vital to regulate mitophagy, among which the N-terminal domain of STX17 is crucially positive to initiate mitophagy, whereas the TPR2 domain of Fis1 is to block mitophagy.

### STX17 on MAM/mitochondria recruits ATG14 for mitophagy

**Fig. 5:**
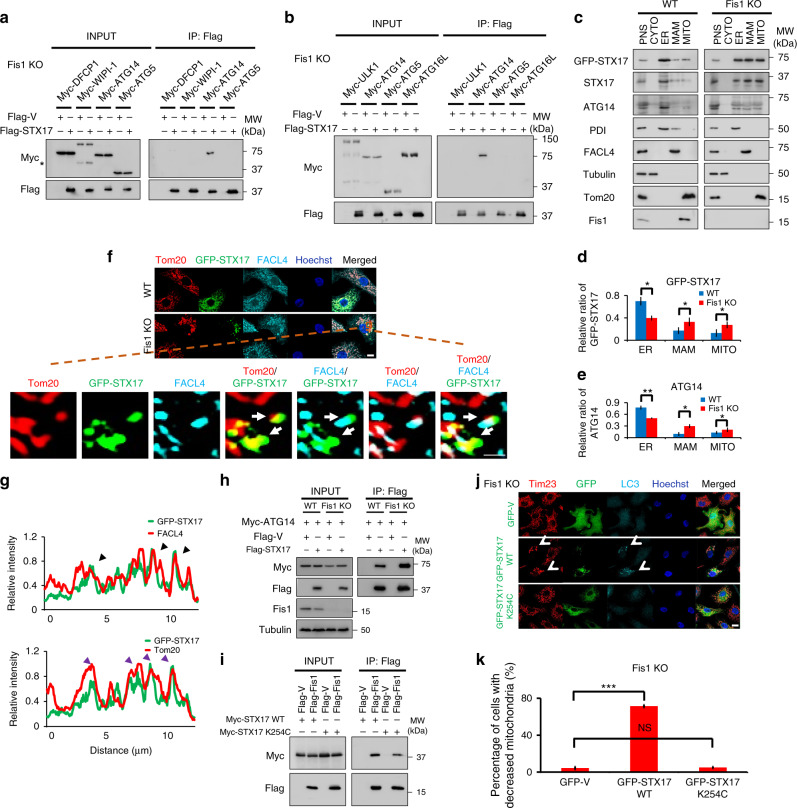
Mitochondrial fission 1 protein (Fis1) regulates the translocation of syntaxin 17 (STX17) onto MAM/mitochondria. **a**, **b** *Fis1 knockout* (*KO*) HeLa cells were transfected with the indicated plasmids. After 24 h, cells were lysed and immunoprecipitated with anti-Flag beads followed by immunoblotting. Asterisk indicates a non-specific band. **c** Immunoblotting of subcellular fractions from wild-type (*WT*) or *Fis1 KO* HeLa cells expressing green fluorescent protein (GFP)-tagged STX17 for 24 h is shown. PNS, post-nuclear supernatant; CYTO, cytosol; ER, endoplasmic reticulum; MAM, mitochondria-associated membranes; MITO, mitochondria. **d**, **e** Quantification of relative ratio of GFP-tagged STX17 (**d**) or endogenous ATG14 (**e**) in the respective subcellular fractions. *P < 0.05, **P < 0.01 (two-tailed unpaired Student’s *t* test from three independent experiments). **f** *WT* or *Fis1 KO* HeLa cells were transfected with GFP-tagged STX17 (green). Cells were imaged following immunostaining for Tom20 (red) and FACL4 (fatty acid-CoA ligase 4) (cyan). Images at single focal plane were acquired. Hoechst, blue. Scale bar, 10 µm. Cropped image was enlarged and shown as a two-dimensional deconvoluted example. White arrows indicate the colocalization of STX17 with Tom20 (mitochondria) and FACL4 (MAM). **g** Line scans corresponding to **f**. The colocalization between GFP-STX17 with FACL4 (MAM) or Tom20 (mitochondria) of *Fis1 KO* cells is indicated by black triangles or purple triangles, respectively. **h** Plasmids encoding respective proteins were transfected into *WT* or *Fis1 KO* HeLa cells for 24 h. Cells were extracted and immunoprecipitated with anti-Flag beads, followed by immunoblotting. **i** Plasmids as indicated were transfected into HeLa cells. After 24 h, cells were extracted and co-immunoprecipitated with anti-Flag beads, followed by immunoblotting with anti-Myc and anti-Flag antibodies, respectively. **j** *Fis1 KO* HeLa cells were transfected with GFP-tagged plasmids (green) as indicated. Cells were fixed and stained with Tim23 (red) and LC3 (cyan) antibodies. Representative confocal microscopy images are shown. Hoechst, blue. Scale bar, 10 µm. Arrowheads indicate mitophagic cells. **k** Mitophagic cells as shown in **j** of *Fis1 KO* cells were quantified. ***P < 0.001, NS, not significant (two-tailed unpaired Student’s *t* test, *n* = 150 cells from three independent replicates). Error bars indicate SD (**d**, **e**, **k**)

To ravel the molecular mechanism by which STX17 overexpression initiates mitophagy under conditions of Fis1 depletion, we examined the interaction of STX17 with various ATG proteins (Fig. 5a, b). Co-immunoprecipitation of Flag-tagged STX17 in *Fis1 KO* cells identified a specific interaction with ATG14, but not ULK1, ATG5, ATG16, WIPI-1, or DFCP1. The recruitment of ATG14 onto mitochondria-associated membranes (MAMs) by STX17 is known to function in the initiation of autophagy^45^. Therefore, we analyzed the accumulation of STX17 and ATG14 on MAM/mitochondria upon Fis1 loss. The subcellular distribution of GFP-STX17 and ATG14 in *WT* or *Fis1 KO* HeLa cells was determined by Percoll density-gradient centrifugation and immunoblotting (Fig. 5c–e). Cellular fractions were identified with respective organelle markers: PDI (protein disulfide isomerase, ER), FACL4 (fatty acid-CoA ligase 4, MAM), Tubulin (cytosol), and Tom20 (mitochondria). GFP-STX17 and ATG14 were substantially enriched on MAM and mitochondrial fractions in response to Fis1 depletion, despite that obvious increase of MAM and mitochondrial STX17 was detected endogenously by Fis1 loss itself (Supplementary Fig. 6a), whereby ATG14 was not found to translocate onto MAM or mitochondrial fractions, indicating that the core autophagy machinery proteins such as ATG14 may be recruited onto mitochondria by dose-dependent regulation of STX17. Approached from immunofluorescence analysis, punctate STX17 colocalized with MAM (FACL4) and mitochondria (Tom20) (Fig. 5f, g). Of note, increased association of ATG14 and STX17 was also observed in *Fis1 KO* cells (Fig. 5h), and this interaction is reliant on the N terminus of STX17 (Supplementary Fig. 6b). To this end, we therefore postulated that mitochondria-associated STX17 initiates mitophagy via the recruitment of ATG14. To further elaborate this issue, we used the STX17 mutant construct (STX17 K254C), which is known to redistribute STX17 on ER^46^. Line-scan analysis confirmed the preferential localization of STX17 K254C mutant on ER (Supplementary Fig. 6c, d). Importantly, STX17 K254C displayed markedly reduced binding affinity with Fis1 (Fig. 5i and Supplementary Fig. 6e), and abrogated mitophagy from 71.4 ± 1.4% of STX17 WT to 5.1 ± 1% of STX17 K254C (Fig. 5j, k). Moreover, we observed significant decreased interaction of STX17 K254C and ATG14, even in *Fis1 KO* cells (Supplementary Fig. 6f–g). Taken together, these data demonstrate that loss of Fis1 triggers the translocation of STX17 and ATG14 onto the mitochondria, which initiates mitophagy.

### Fis1 regulates mitophagy via hierarchical autophagy model

**Fig. 6:**
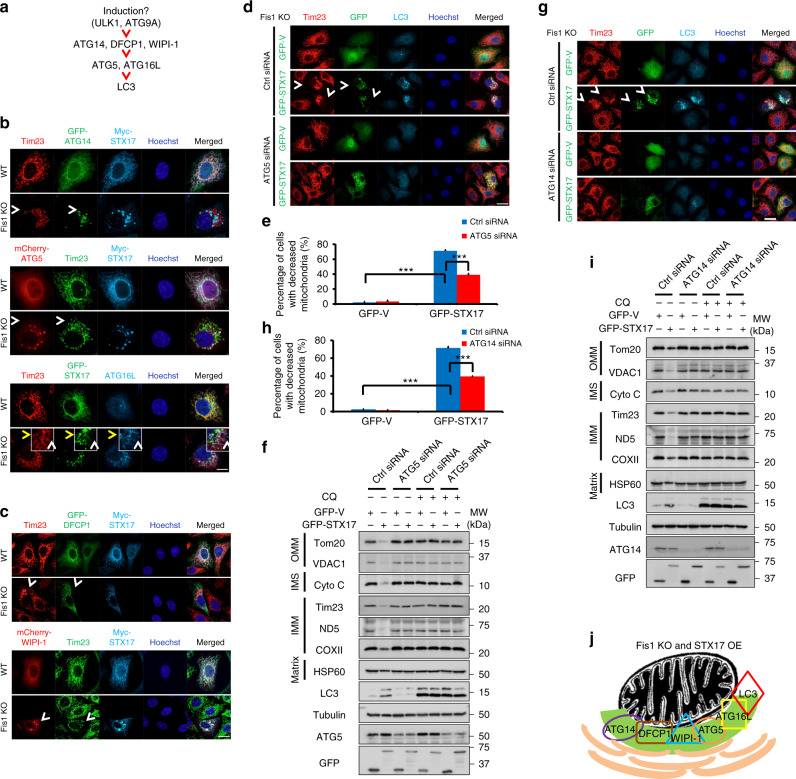
Syntaxin 17 (STX17) initiates mitophagy in the hierarchical autophagic pathway. **a** The schematic model illustrating the genetic hierarchy and temporal relationship of autophagy proteins. **b**, **c** *Wild-type* (*WT*) or *mitochondrial fission 1 protein knockout* (*Fis1 KO*) HeLa cells were transfected with the indicated plasmids for 24 h. Cells were fixed and imaged by fluorescence microscopy. Hoechst, blue. Scale bar, 10 µm. Arrowheads indicate mitophagic cells. **d**, **g** *Fis1 KO* cells were transfected with respective small interfering RNA (siRNA) to silence the indicated genes for 24 h. Then, green fluorescent protein (GFP)-tagged vector or STX17 (green) was transfected into cells for further 24 h. Mitochondrial Tim23 (red) and LC3 (cyan) signals are shown by confocal microscopy. Hoechst, blue. Scale bar, 10 µm. Arrowheads indicate mitophagic cells. **e**, **h** Quantification of mitophagy percentage of **d** or **g**. Error bar represents SD. ***P *<* 0.001 (two-tailed unpaired Student’s *t* test from three independent experiments, *n* = 150). **f, i** Fis1 KO HeLa cells were transfected with the indicated siRNA for 24 h and then treated with respective siRNA again plus GFP-tagged vector or STX17 for further 6 h. Hereafter, cells were cultured in medium with or without chloroquine (CQ) for another 66 h. Cell lysates were immunoblotted for outer mitochondrial membrane (OMM), intermembrane space (IMS), inner membrane mitochondrial (IMM), and matrix proteins. **j** A schematic summary of the recruitment of canonical autophagy proteins for STX17 overexpression-induced mitophagy of *Fis1 KO* cells

Given that STX17 and ATG14 are crucial for mitophagy in *Fis1 KO* cells, we then attempted to investigate whether canonical autophagy machinery could be involved hierarchically (Fig. 6a). We observed no satisfactory differences in ULK1 and ATG9A localization with or without STX17-induced mitophagy upon Fis1 loss (Supplementary Fig. 7a, b). However, when we studied the localization of downstream autophagy markers in *Fis1 KO* cells during STX17-induced mitophagy, we found ATG14, ATG5, ATG16 (Fig. 6b), DFCP1 and WIPI-1 (Fig. 6c) formed distinguished puncta, recruited onto decreased mitochondria. Complementarily, when we silenced ATG5 (Fig. 6d, e) or ATG14 (Fig. 6g, h), STX17-induced mitophagy of *Fis1 KO* cells was reduced from 71 ± 1.3 to 39 ± 1.7% or from 71.4 ± 1.7 to 39.4 ± 0.5% respectively, which is supported by immunoblotting, whereby lysosomal inhibitor CQ significantly blocked the overall degradation of mitochondrial proteins (Fig. 6f, i). Meanwhile, we found that ATG14 and ATG5 are also essential for the puncta formation of STX17 (Supplementary Fig. 7c–e), integrating the regulation of mitophagy and the self-oligomerization of STX17. Importantly, although Beclin1 interacted with ATG14 (Supplementary Fig. 7f), which is known to form phosphoinositide 3-kinase (PI3K) complex^50^, we found that Beclin1 lacked the effect to induce mitophagy in Fis1-depleted cells, unlike STX17. Similarly, no appreciable mitophagy was initiated by PI3K complex downstream effectors, including DFCP1 and WIPI-1 (Supplementary Fig. 7g, h). In addition, other SNARE proteins such as SNAP29 and VAMP8 show failure in mitophagy induction (Supplementary Fig. 7i), suggesting that the specific regulation between Fis1 and STX17 is the key to mitophagy, among which STX17 assembles autophagy proteins to mediate mitophagy processively. Collectively, our results illustrate that, upon Fis1 deficiency, STX17 translocates onto MAM/mitochondria, recruiting ATG14 and activating the downstream of hierarchical autophagy machinery sequentially, to target mitochondria for degradation (Fig. 6j).

### Rab7 and TFEB mediate STX17-induced mitophagy

**Fig. 7:**
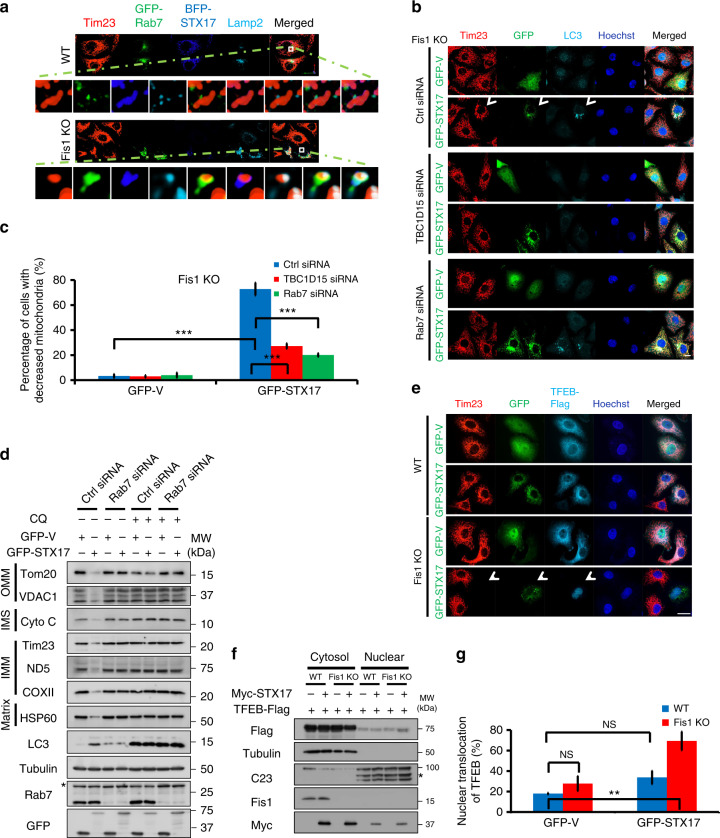
Transcription factor EB (TFEB) and Rab7 are involved in syntaxin 17 (STX17)-initiated mitophagy upon mitochondrial fission 1 protein (Fis1) loss. **a** Wild-type (*WT*) or *Fis1 knockout* (*KO*) HeLa cells were co-transfected with plasmids encoding green fluorescent protein (GFP)-Rab7 (green) and blue fluorescent protein (BFP)-STX17 (blue) for 24 h. Cells were fixed and immunostained for Tim23 (red) and Lamp2 (cyan). Cells were visualized as three-dimensional reconstruction images. **b** *Fis1 KO* HeLa cells were transfected with control-, *TBC1D15*-, or *Rab7*-small interfering RNA (siRNA) for 24 h. Cells were transiently transfected with GFP-tagged vector or STX17 (green) for further 24 h. Mitochondria and autophagosome were highlighted by Tim23 (red) and LC3 (cyan). Hoechst, blue. Scale bar, 10 µm. Arrowhead indicates mitophagic cell. **c** Quantification of mitophagic cells in **b**. ***P < 0.001 (two-tailed unpaired Student’s *t* test from three independent experiments, *n* = 150). **d** *Fis1 KO* cells were transfected with control- or *Rab7*-siRNA for 24 h. Cells were then again transfected with respective siRNA, plus GFP-tagged vector or STX17 for further 6 h. Hereafter, cells were cultured with or without chloroquine (CQ) for another 66 h. Cells were harvested and lysed, followed by sodium dodecyl sulfate-polyacrylamide gel electrophoresis (SDS-PAGE) gel separation and immunoblotting against overall mitochondrial proteins. **e** *WT* or *Fis1 KO* HeLa cells were co-transfected with GFP-tagged vector or STX17 (green) and TFEB-Flag (cyan) for 24 h. Cells were fixed and immunostained against Tom20 (red) and Flag (cyan). Hoechst, blue. Scale bar, 10 µm. Arrowhead indicates mitophagic cell. **f** *WT* or *Fis1 KO* HeLa cells treated as in **e** were fractioned, lysed, and immunoblotted. Asterisk indicates non-specific bands. **g** Quantification of **f**. TFEB-Flag expression level was normalized to Tubulin (cytosol) or C23 (nuclear). Nuclear TFEB-Flag was indicated as the percentage of total TFEB-Flag. **P < 0.01, NS, not significant (two-tailed unpaired Student’s *t* test, *n* = 3). Error bars represent SD (**c** and **g**)

Previous studies have demonstrated that endosomal Rab7 governs mitophagosome formation and influences mitophagy^47,51^; here we aimed to examine the role of Rab7 in STX17-induced mitophagy of Fis1-depleted cells. In *Fis1 KO* cells, blue fluorescent protein (BFP)-tagged STX17 was found to engulf mitochondria (Tim23), which partially colocalized with Lamp2-positive lysosomal structures. Interestingly, GFP-Rab7 enclosed this mitophagosome (Fig. 7a), albeit endogenous Rab7 was not pronounced by Fis1 loss (Supplementary Fig. 8a). To determine the importance of Rab7 for STX17-induced mitophagy, we then manipulated Rab7 cycle by depleting either Rab7 or TBC1D15, the GTPase-activating protein for Rab7. Loss of Rab7 or TBC1D15 dramatically impaired STX17-induced mitophagy in *Fis1 KO* cells from 72.8 ± 2.1% to 20.1 ± 1.4% or 27.2 ± 2.1%, respectively (Fig. 7b, c). Degradation of overall mitochondrial proteins was also blocked in the presence of *Rab7* siRNA and lysosomal inhibitor CQ essentially blocked mitochondrial turnover (Fig. 7d). In addition, the dominant-negative (DN) form of Rab7 (Rab7 T22N) was found to interfere with STX17 overexpression-mediated mitophagy, from 65 ± 2.7% of Rab7 WT-treated cells to 16.6 ± 1.9% of Rab7 DN-treated cells (Supplementary Fig. 8b, c), indicating that the GTP/GDP cycle of Rab7 accounts for mitophagy. In contrast, Rab5 DN had no impact on mitophagy mediated by STX17 overexpression in *Fis1 KO* cells (Supplementary Fig. 8d, e). As expected, without the overexpression of STX17, Rab7 WT or even the constitutive active form of Rab7 (Rab7 CA) lacked the initiative function to induce mitophagy, albeit in the conditions of Fis1 depletion (Supplementary Fig. 8f), illustrating that the induction of mitophagy relies on the specific regulation between Fis1 and STX17. Taken together, these results reveal that Rab7, most likely dependent on its GTPase activity, is essential for STX17-induced mitophagy in the absence of Fis1.

We then wondered the importance of lysosomal activation for STX17-initiated mitophagy in *Fis1 KO* cells. To address this, we analyzed the subcellular localization of the transcription factor EB (TFEB), which controls lysosome biogenesis by coordinating the expression of proteins involved in lysosome function and autophagy^52,53^. During STX17 overexpression-mediated mitophagy in *Fis1 KO* cells, TFEB was dephosphorylated, allowing its translocation from the cytosol to the nucleus (Fig. 7e–g), demonstrating that lysosomal activation occurs during STX17 overexpression-mediated mitophagy.

### Fis1 depletion impairs mitochondrial respiration

**Fig. 8:**
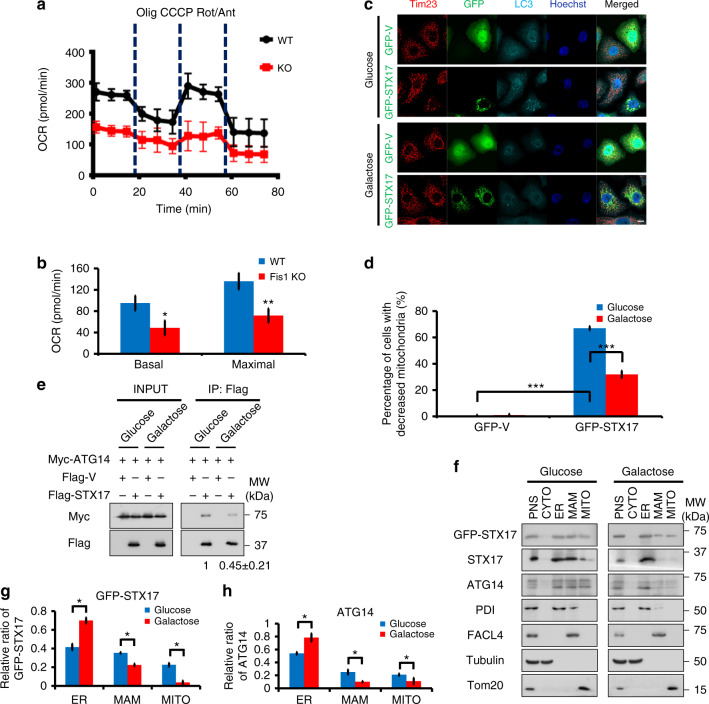
Mitochondrial fission 1 protein (Fis1) depletion accompanies mitochondrial respiration dysfunction. **a** Oxygen consumption rate (OCR) was measured in wild-type (*WT*) and *Fis1* knockout (*KO*) HeLa cells. Dashed vertical lines indicate the addition of 1 µM oligomycin (Olig), 0.5 µM carbonyl cyanide 3-chlorophenylhydrazone (CCCP), and 1 µM rotenone plus 1 µM antimycin A (Rot/Ant), respectively (*n* = 8). **b** Individual parameters for basal respiration and maximal respiration analysis of **a**. **P < 0.01, *P < 0.05 (two-tailed unpaired Student’s *t* test, *n* = 3). **c** Glucose or galactose-cultured *Fis1 KO* HeLa cells were transiently transfected with green fluorescent protein (GFP)-tagged vector or syntaxin 17 (STX17) for 24 h. Cells were fixed and immunostained with anti-Tim23 (red) and anti-LC3 (cyan) antibodies. Hoechst, blue. Scale bar, 10 µm. **d** Quantification of **c**. ***P < 0.001 (two-tailed unpaired Student’s *t* test). *n* = 150 GFP-positive cells/group from three replicates. **e** Plasmids as indicated were transfected into *Fis1 KO* HeLa cells. At 6 h post-transfection, cells were cultured in medium with glucose or galactose for further 18 h. Cells were extracted and co-immunoprecipitated with anti-Flag beads, followed by immunoblotting with respective antibodies. The fold change of the interaction between ATG14 and STX17 is shown as mean ± SD from three independent experiments. **f** GFP-tagged STX17 was transfected into *Fis1 KO* HeLa cells for 6 h. Then, cells were cultured in medium with glucose or galactose for further 18 h. Immunoblotting of subcellular fractions were analyzed. PNS, post-nuclear supernatant; CYTO, cytosol; ER, endoplasmic reticulum; MAM, mitochondria-associated membranes; MITO, mitochondria. **g**, **h** Quantification of relative ratio of GFP-tagged STX17 (**g**) or endogenous ATG14 (**h**) in the respective subcellular fractions. *P < 0.05 (two-tailed unpaired Student’s *t* test, *n* = 3). Error bars represent SD (**a**, **b**, **g** and **h**)

To understand the underlying physiological basis for triggering STX17 overexpression-mediated mitophagy by loss of OMM protein Fis1, we hypothesized that Fis1 deficiency impairs mitochondrial function, which in turn signals for mitochondrial removal. Notably, we observed a dramatic decrease of oxygen consumption rate (OCR) of *Fis1 KO* cells (Fig. 8a, b), indicating a defect of mitochondrial respiration. Indeed, our data were complementarily supported by a previous study in which mitochondrial respiratory function was assessed in INS1 cells expressing *Fis1* RNA interference^54^. This study reached essentially the same conclusion that reduction of Fis1 expression decreases mitochondrial respiratory capacity. To further characterize the effect of mitochondrial respiration dysfunction on STX17 overexpression-mediated mitophagy, we cultured cells in medium containing galactose instead of glucose, to push cells to increase reliance on oxidative phosphorylation (OXPHOS). Interestingly, in OXPHOS-inducing medium, STX17 overexpression-mediated mitophagy induced by Fis1 loss was substantially suppressed from 67 ± 1.6% in the glucose medium to 31.9 ± 2.8% by the galactose medium (Fig. 8c, d), indicating that mitochondrial metabolism is pivotal for STX17 overexpression-mediated mitophagy. Complementarily, a substantial reduction of interaction between ATG14 and STX17, by 0.55 ± 0.21-fold, was observed using galactose culture, even in Fis1-depleted cells (Fig. 8e). In addition, galactose pronouncedly abrogated the translocation of STX17 and ATG14 onto MAM and mitochondrial fractions (Fig. 8f–h). Collectively, these results elaborate that mitochondrial respiration dysfunction, coupled with Fis1 loss, primes cells for STX17 overexpression-mediated mitophagy.

### Effects of mitophagy receptors on STX17-initiated mitophagy

Among the studies of PINK1/Parkin-independent mitophagy, selective mitochondrial clearance could be mediated by well-characterized mitophagy receptors, including OPTN (optineurin), NDP52 (also called as CALCOCO2, calcium-binding and coiled-coil domain-containing protein 2), Nix, FUNDC1, and PHB2 (prohibitin-2)^19,26,27,55–57^. Destined damaged mitochondria are recognized by mitophagy receptors, which bridge the recruitment of autophagosomal protein LC3 to engulf mitochondria, followed by the lysosomal digestion. Given this, we attempted to examine whether these typical mitophagy receptors would be responsible for STX17-initiated mitophagy. Of note, no appreciable suppression of STX17-initiated mitophagy was observed when canonical mitophagy receptors were silenced (Supplementary Fig. 9a–c), providing evidence that these well-established mitophagy receptors possess minimal impacts on STX17 overexpression-mediated mitophagy upon Fis1 loss.

## Discussion

**Fig. 9:**
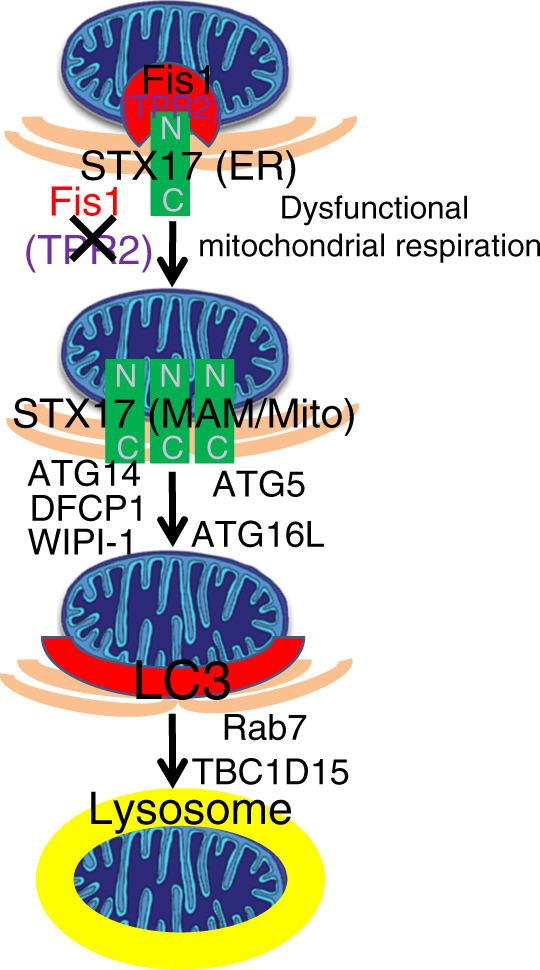
Model. Hypothetical model of syntaxin 17 (STX17)-induced mitophagy upon mitochondrial fission 1 protein knockout (*Fis1 KO*)

In this study, we report a PINK1/Parkin-independent pathway of mitophagy, mediated by the SNARE protein, STX17, which is triggered by the loss of OMM protein, Fis1. Under basal conditions, Fis1 acts as a gatekeeper, governing dynamic trafficking of STX17 from ER to mitochondria, and preventing the exposure and oligomerization of the N terminus of STX17. Loss of Fis1 primes the over-transportation of STX17 onto MAM and mitochondria, subsequently recruiting ATG14. The PI3K complex containing ATG14 further nucleates isolation membrane to initiate mitophagy. Mitophagy then proceeds in a hierarchical macroautophagic manner, in which the assembly of phagophore membranes (DFCP1, WIPI-1, ATG5, and ATG16L) complete. Mature mitophagosomes fuse with lysosomes, mediated by Rab7 and TBC1D15. Consequently, mitochondria dysfunctional in respiration, as the cargoes, are eliminated, extending to a model of canonical autophagy-involved mitophagy (Fig. 9).

The regulation by PINK1/Parkin has been considered as the principle pathway mediating mitophagy^21,22,58^. While structures containing non-selective autophagy proteins, such as ATG9, ULK1, and FIP200, have been shown to be employed by the PINK1/Parkin pathway^9,19,51^, the question has remained whether effectors of bulk degradation (i.e., autophagy) could initiate mitophagy directly. Growing evidence indicates that selective autophagy is mediated by organelle receptors which recognize cargoes tagged with degradation signals, under conditions of certain stress or damage. For instance, in the case of mitophagy, such prevalent signals include mitochondrial depolarization^19,21–23^ or hypoxic stress^25,26,59^. However, it is elusive whether mitophagy could undergo in an autonomous manner, without ectopic stimulation. Here our findings provide an additional mechanism in mammalian cells by which mitochondrial removal occurs via the deficiency of Fis1, which allows STX17 to initiate mitophagy in use of a non-selective autophagy machinery.

While the role of Fis1 in mammalian mitochondrial fission has been cast into doubt, we here demonstrate an important function of Fis1 in regulating mitochondrial quality control. In agreement with previous studies showing that Fis1 is not required for Drp1 recruitment in mitochondrial fission^34,47^, we observed no gross difference in the recovery rate and level properties between *WT* and *Fis1 KO* HeLa cells after mitochondrial photobleaching (Supplementary Fig. 10a), indicative of that Fis1 is indeed dispensable for mitochondrial dynamics. Additionally, although accumulated evidence indicates that mitochondrial fission accelerates mitophagy, whereas mitochondrial fusion suppresses mitophagy^29,54,60,61^, here we elaborate that mitochondrial dynamics is unlikely to be implicated in STX17 overexpression-mediated mitochondrial clearance. In sight of this, we found fission-to-fusion imbalance by silencing *Mff*, *Drp1*, *MiD49*, *MiD51* (Fig. 2a, b), *Mfn1*, and *Mfn2* (Supplementary Fig. 10b, c) failed to prime cells to STX17-initiated mitophagy. Complementarily, manipulation of mitochondrial morphology by knocking down *Drp1* or *Mff* in *Fis1 KO* cells showed no appreciable impact on STX17-induced mitophagy (Supplementary Fig. 10d, e), supporting the notion that Fis1 regulates mitophagy negatively, independent on mitochondrial dynamics, but by specifically restraining the trafficking of STX17 onto MAM and mitochondria.

Notably, growing evidence has indicated the positive regulation of Fis1 on PINK1/Parkin-dependent mitochondrial removal, in which low levels of Fis1 abrogates mitophagy^47,48^. It light of this, we reason that Fis1 may play differential roles in distinct pathways of mitophagy. In STX17 overexpression-mediated mitophagy, which is uncoupled to PINK1/Parkin, lack of Fis1 autonomously sensitizes cells to mitophagy, via a hierarchical autophagic pathway. In contrast, during PINK1/Parkin-dependent mitophagy, high level of Fis1 is pivotal through TBC1D15 and Rab7, for mitophagosome biogenesis^47^. These leave it unknown what, if any, specific stimuli to provoke essentiality of Fis1 on either pathway. Clearly, more needs to be studied about the regulatory function of Fis1 on mitophagy. Importantly, while this work was under revision, an interesting study reported that Fis1 is vital for the accumulation of misfolded proteins in the mitochondrial matrix, involved in FUNDC1-dependent aggrephagy^62^. However, the regulation of Fis1 on basal mitophagy may be dissimilar from the aggrephagy under proteostatic stress. In addition, STX17-mediated mitophagy may undergo a specific route, independent of canonical mitophagy receptors including FUNDC1 (Supplementary Fig. 9). Strikingly, the notion that STX17, on MAM, assists the cooperation between FUNDC1 and PGAM5 for mitochondrial removal has been well characterized in the field of PINK1/Parkin-mediated mitophagy^63^. However, STX17 may also possess distinct regulation on PINK1/Parkin-independent mitochondrial clearance. Strikingly, here we illustrate that overexpressed STX17, dynamically regulated by Fis1, autonomously initiates mitophagy in HeLa cells devoid of Parkin, suggesting STX17 performs selectively in distinctive pathways for mitophagy. Given this, it would be important to reveal modulators on ER–mitochondrial interface accounting for different routes of mitophagy mechanistically, in a larger scale. Of note, our observations highlight a fundamental concept that, under basal conditions, mitochondria may require alternative mechanisms to escape from autophagy (mitophagy), in which a certain expression level of Fis1 must be maintained to play the role as gatekeeper to govern the formation of mitophagosome. Furthermore, at physiological level regarding mitochondrial homeostasis, we show that Fis1 regulates mitochondrial respiration, providing new insights into the relationship between OXPHOS dependency and mitophagy biogenesis.

In summary, here we identify a PINK1/Parkin-independent mitophagic pathway, via the cooperation of autophagy protein, STX17, and mitochondrial protein, Fis1. Informatively, Fis1 expression has been reported to link to glucose responsiveness in pancreatic beta cells^64^. It has been found that insulin secretion in INS1 cells is reliant on Fis1 level and dysregulation of interface between nutrient input and insulin secretion is highly impacted in type 2 diabetes mellitus. It is tempting to speculate that STX17-induced mitophagy could be crucial for governing cellular quality control in pancreas system. However, it is unclear to what extent the STX17-initiated mitophagy would be relevant to different physiological states. More needs to be learned about regarding this issue. Delineating the causative factor and sequential consequences of STX17-initiated mitophagy, regulated by Fis1, may lead to greater insights into interventions on diseases, particularly for therapeutics on neurodegenerative diseases. Additionally, the effects of STX17 over-translocation onto the mitochondria, which possibly induces mitochondrial damage, are mysterious. Study to clarify the importance of STX17 in mitochondrial quality control remains as another intriguing area of future work.

## Methods

### Antibodies

Anti-ATG9A (Cell Signaling Technology, 13509, 1:200), anti-ATG14 (Cell Signaling Technology, 5504, 1:500), anti-ATG16L (MBL international, PM040, 1:200), anti-cytochrome *c* (BD Bioscience, 556433, 1:1000), anti-cytochrome *c* oxidase subunit II (Abcam, ab79393, 1:1000), anti-C23 (Santa Cruz, sc-8031, 1:1000), anti-Drp1 (BD Bioscience, 611112, 1:2000), anti-Fis1 (GeneTex, GTX111010, 1:1000), anti-FLAG (Sigma-Aldrich, F3165, 1:2000), anti-FUNDC1 (AVIVA, ARP53280_P050, 1:500), anti-GFP (Santa Cruz, sc-9996, 1:1000), anti-Hsp60 (Santa Cruz, sc-1052, 1:2000), anti-Lamp2 (Santa Cruz, sc-271800, 1:200), anti-LC3-II (Cell Signaling, 3868, 1:2000 and MBL, PM036, 1:500), anti-Mff (Abcam, 139026, 1:1000), anti-Mfn1 (Santa Cruz, sc-50330, 1:1000), anti-Mfn2 (Santa Cruz, sc-100560, 1:1000), anti-MiD49 (Proteintech, 16413-1-AP, 1:1000), anti-MiD51 (Proteintech, 20164-1-AP, 1:1000), anti-MT-ND5 (Abcam, ab92624, 1:1000), anti-Myc (Sigma-Aldrich, C3956, 1:1000), anti-NDP52 (Abcam, ab68588, 1:1000), anti-Nix (GeneTex, GTX111876, 1:1000), anti-OPTN (Proteintech, 10837-1-AP, 1:1000), anti-PDI (Cell Signaling Technology, 3501, 1:1000), anti-P62 (MBL International, PM045, 1:500), anti-Rab7 (Abcam, ab50533, 1:1000 and Cell Signaling Technology, 9367, 1:200), anti-STX17 (Sigma-Aldrich, HPA001204, 1:1000 and Thermo Fisher Scientific, PA5-40127, 1:2000), anti-TBC1D15 (Abcam, ab121396, 1:1000), anti-Tim23 (BD Bioscience, 611222, 1:1000), anti-Tom20 (Santa Cruz, sc-17764, 1:2000), anti-Tubulin (Sigma-Aldrich, T5168, 1:30,000) and anti-VDAC1 (Santa Cruz, sc-8828, 1:1000).

### Plasmids

Flag-Fis1 was kindly provided by Dr. Victor Chun-Kong Yu (National University of Singapore, Singapore) and then subcloned into **mCherry-pXJ40** and **GFP-pXJ40** vectors. Flag-STX17 (#45911) was purchased from Addgene and subcloned into **pXJ40** vectors with Myc, GFP, mCherry, and BFP tags. GFP-Parkin was kindly provided by Dr. Kah-Leong Lim (National University of Singapore, Singapore)^65^. Truncations of Fis1 and STX17 and site-directed mutations of STX17 were generated using a QuikChange II site-directed mutagenesis kit (Agilent Technologies). Myc-ULK1 and Myc-ATG5 were kindly provided by Dr. Shengcai Lin (Xiamen University, China). pEGFP-ATG14 (#21635), SECFP-ATG16L (#58994), GFP-WIPI-1 (#38272), GFP-DFCP1 (#86746), and retrovirus plasmid GFP-STX17 (#45909) were purchased from Addgene. GFP-Rab7, GFP-Rab5, and their site-directed mutants were kind gifts from Dr. Alexander Bershadsky (National University of Singapore, Singapore). Beclin1 was purchased from Addgene (#21150) and subcloned into Flag-pXJ40 vector. Mito-RFP and PSICOR-mt-Keima plasmids were kindly provided by Dr. Quan Chen (Nankai University, China). All primer sequences used for cloning are listed in Supplementary Data 1.

### RNA interference

Oligonucleotides for siRNA were synthesized by Invitrogen or Sigma-Aldrich, and the sequences were as follows:

*ATG5* siRNA: 5′-CCUUUCAUUCAGAAGCUGU-3′; *ATG14* siRNA: 5′-GGCAAAUCUUCGACGAUCCCAUAUA-3′; Control siRNA: 5′-UUCUCCGAACGUGUCACGU-3′; *Drp1* siRNA: 5′-UUCAAUCCGUGAUGAGUAUGCUUUUCUUC-3′; *Fis1* siRNA: 5′-AACGAGCUGGUGUCUGUGGAG-3′; *FUNDC1* siRNA: 5′-GCAGCACCUGAAAUCAACA-3′; *Mfn1* siRNA: 5′-CUUCCUAAGUGUUGAAGGA-3′; *Mfn2* siRNA: 5′-GUGAUGUGGCCCAACUCUA-3′; *MiD49* siRNA: 5′-ACUUUCGGAGCAAGUUCCCGGAACU-3′; *MiD51* siRNA: 5′-GCCAAGCAAGCUGCUGUGGACAUAU-3′; *Mff* siRNA: 5′-AACGCUGACCUGGAACAAGGA-3′; *NDP52* siRNA: 5′-UUCAGUUGAAGCAGCUCUGUCUCCC-3′; *OPTN* siRNA: 5′-CCACCAGCUGAAAGAAGCC-3′; *PHB2* siRNA: 5′-AGAUUCGAGCAGCCCAGAAUAUCUC-3′; *Rab7* siRNA: 5′-CACGUAGGCCUUCAACACAAU-3′; *STX17* siRNA: 5′-GAAAGTCCGAAAGGATGACCTAGTA-3′; *TBC1D15* siRNA: 5′-GAACCAGGAUUUGAAGUCAUCACAA-3′.

### Cell culture and transfection

HeLa, HEK 293T cells, and SH-SY5Y were purchased from ATCC. HeLa cells stably expressing GFP-Parkin was kindly provided by Dr. Kah-Leong Lim (National University of Singapore, Singapore). Cells were cultured in Dulbecco’s modified Eagle's medium (DMEM) (HyClone), and then supplemented with 10% (v/v) fetal bovine serum (FBS, GE Healthcare Hyclone) at 37 °C and 5% CO_2_.

Lipofectamine 2000^TM^ (Invitrogen) was used to transfect siRNA or plasmids into HeLa cells at 70% confluence, according to the manufacturer’s instructions.

Carbonyl cyanide 3-chlorophenylhydrazone (CCCP, Sigma-Aldrich) was dissolved in dimethyl sulfoxide and used at a final concentration of 10 µM. CQ (Sigma-Aldrich) was dissolved in phosphate-buffered saline (PBS) and used at a working dose of 10 µM.

### Conduction of *Fis1 KO* HeLa cells

To generate *Fis1* *KO* HeLa cells, hCas9 WT of lentiCRISPR v2 (Addgene, #52961) was modified to hSpCas9 nickase, which was then inserted before the EFS-NS promoter together with the Golden Gate assembly cloning cassette from pLV hUbC-Cas9-T2A-GFP (Addgene, #53190)^66,67^. One of the BsmB1 overhangs, GGAC, in phH1-gRNA (Addgene, #53186) was modified to CTAT for one pair of single guide RNA (sgRNA) assembly. Fis1-sgRNA1 AGACACCAGCTCGTTCAGCA and Fis1-sgRNA2 GTGAGGCCTGGCCCGGACAG were targeted to Fis1 exon1. Two sgRNAs were cloned into modified phH1-gRNA and ph7SK-gRNA vectors (Addgene, #53189), respectively. Then, the modified lentiCRISPR v2, Fis1-sgRNA1 and Fis1-sgRNA2 were integrated into one construct using the Golden Gate Assembly method. Two homolog arms of *Fis1* gene (−1045 to 13) and (63 to 1165) were inserted to flank the hCas9n-P2A-puro cassette. The construct was then transfected into HeLa cells and selected with 2 µg ml^−1^ puromycin with serial dilutions to obtain the *Fis1 KO* clone.

### Generation of stable cell lines

To establish HeLa cells stably expressing GFP-STX17, *WT* or *Fis1 KO* HeLa cells stably expressing mt-Keima, retroviruses (GFP-STX17), or lentiviruses (mt-Keima) were packaged in HEK293T cells. HeLa cells were then transduced with respective viruses for 72 h with 8 µg ml^−1^ polybrene (Sigma), and further optimized for interested protein expression via selection (puromycin for GFP-STX17) or fluorescence sorting (mt-Keima).

### Immunofluorescence and image acquisition

Cells were fixed in 4% paraformaldehyde/PBS at room temperature for 10 min, then washed with PBS, permeabilized, and blocked in 3% FBS/PBS containing 0.2% Triton X-100. Primary antibodies and Alexa Fluor-conjugated secondary antibodies (Thermo Fisher Scientific, 1:200) were diluted in 3% FBS/PBS and PBS, respectively, and then incubated with cells sequentially. Samples were then washed with PBS and stained with Hoechst 33342 (Invitrogen, 1:1000) at room temperature for 10 min to label the nucleus. Coverslips were then mounted onto glass slides using FluorSave^TM^ (Calbiochem).

Confocal slices (<10 µm) were acquired via a laser scanning confocal microscope (Olympus FV3000) through a 63 × 1.4 NA objective lens or a spinning disc microscope (Volocity^TM^, PerkinElmer) through a 100 × 1.4 NA objective lens using excitation wavelengths of 405, 488, 561, and 640 nm. Live-cell imaging was performed in a chamber heated to 37 °C at 5% CO_2_.

For structure illumination microscopy (SR-SIM) imaging, fixed samples or live cells were imaged by Nikon SIM attached to a Ti-E-inverted microscope (Nikon) with Perfect Focus System of 100 × oil immersion objective (1.40 NA, CFI Plan-ApochromatVC). Images were acquired with DU-897 cameras (Andor Technology) and NIS-Elements AR software (Nikon) was used to control the acquisition. Cells were imaged at a step size of 0.5 µm with a total height of 15 µm.

For FRAP (fluorescence recovery after photobleaching) analysis, *WT* or *Fis1 KO* HeLa cells transiently expressing RFP-Mito were monitored with the live-cell imaging system using the Ultraview Vox spinning disc confocal microscope (PerkinElmer). The 561 nm laser was used to bleach a 2 × 2 μm^2^ area placed on the mitochondrial network fiber. A recovery time of 15 s for each bleaching was used to make sure the recovery has reached the plateau. The intensity of Mito-RFP fluorescence in the bleached area from 15 different cells was measured and analyzed^37^.

### Subcellular fractionation

For the extraction of mitochondrial membranes, cells cultured in 10 cm dishes were washed with PBS and collected using pre-chilled mitochondrial extraction buffer 1 (220 mM mannitol, 70 mM sucrose, 20 mM HEPES-KOH, pH 7.5, 1 mM EDTA and 2 mg^−1^ ml^−1^ bovine serum albumin) supplemented with protease inhibitors including 10 μg^−1^ ml^−1^ aprotinin, 1 mM phenylmethylsulfonyl fluoride, 1 μM pepstatin, and 10 μM leupeptin. Cells were passed through a 25-G syringe (BD Biosciences) 20 times on ice. The homogenized cells were centrifuged at 1000 × *g* for 15 min at 4 °C. The supernatant was further centrifuged at 10,000 × *g* for 10 min at 4 °C to pellet the mitochondria. The supernatant fraction was retained as the cytosolic fraction. The mitochondrial pellets were resuspended in RIPA buffer (50 mM HEPES, pH 7.4, 150 mM NaCl, 1% NP-40, 0.1% SDS, 0.25% sodium deoxycholate and 1 mM EDTA) supplemented with phosphatases inhibitors, including 20 mM NaF, 0.2 mM Na_3_VO_4_, and protease inhibitors, and then subjected to SDS-PAGE and immunoblotting^68^.

For the extraction of ER, MAMs, and mitochondrial fractions, 40 confluent plates in 10 cm dishes of HeLa cells were collected and resuspended in ice‐cold buffer 2 (225 mM mannitol, 75 mM sucrose, 0.1 mM EGTA, and 30 mM Tris-HCl, pH 7.4, containing protease inhibitors including 10 μg^−1^ ml^−1^ aprotinin, 1 mM phenylmethylsulfonyl fluoride, 1 μM pepstatin, and 10 μM leupeptin). After gentle homogenization with a Dounce homogenizer (200 strokes), cell extractions were centrifuged at 600 × *g* for 5 min at 4 °C (PNS). The supernatant was further centrifuged at 7000 × *g* for 10 min at 4 °C to obtain the crude mitochondrial pellet. The supernatant was centrifuged at 100,000 × *g* for 1 h at 4 °C to result in ER (pellet) and cytosolic (supernatant) fractions. The crude mitochondrial pellet was gently resuspended in 2 ml of ice‐cold buffer 3 (250 mM mannitol, 5 mM HEPES, pH 7.4, and 0.5 mM EGTA with protease inhibitors, including 10 μg^−1^ ml^−1^ aprotinin, 1 mM phenylmethylsulfonyl fluoride, 1 μM pepstatin, and 10 μM leupeptin) and overlaid onto 8 ml of Percoll medium in an ultracentrifuge tube. Centrifugation was carried out at 95,000 × *g* for 60 min at 4 °C to isolate the MAM and pure mitochondria^69,70^.

For the separation of cytosolic and nuclear fractions, cells cultured in 10 cm dishes were washed with PBS, collected and lysed in 400 µl buffer 4 (50 mM HEPES, 150 mM NaCl, and 100 µg^−1^ ml^−1^ digitonin) supplemented with protease inhibitors, including 10 μg^−1^ ml^−1^ aprotinin, 1 mM phenylmethylsulfonyl fluoride, 1 μM pepstatin, and 10 μM leupeptin. Cells were then rotated at 4 °C for 10 min. Cell homogenates were centrifuged at 4000 × *g* for 2 min at 4 °C to obtain the cytosolic fraction. The pellets were washed twice with cold PBS and then solubilized in 100 µl buffer 5 (50 mM HEPES, 150 mM NaCl, 0.5% sodium deoxycholate, and 0.1% sodium dodecyl sulfate) for 15 min at 4 °C. Soluble solution was separated from the nuclei-enriched pellets by centrifuging at 13,000 × *g* for 5 min at 4 °C and obtained as nuclear fraction.

### Immunoblotting and immunoprecipitation

Collected cells were lysed in RIPA buffer (50 mM HEPES, pH 7.4, 150 mM NaCl, 1% NP-40, 0.1% SDS, 0.25% sodium deoxycholate and 1 mM EDTA) supplemented with protease inhibitors including 10 μg^−1^ ml^−1^ aprotinin, 1 mM phenylmethylsulfonyl fluoride, 1 μM pepstatin, and 10 μM leupeptin. Equivalent proteins were loaded, separated by SDS-PAGE, and detected by immunoblotting using Amersham Imager 600 (GE Healthcare Life Sciences). Uncropped scans of all western blots are shown in the Supplementary Figs. 11–15. For immunoprecipitation, cells were collected and lysed in 1 ml NP-40 lysis buffer (50 mM Tris-HCl, pH 7.4, 150 mM NaCl, 10 mM sodium pyrophosphate, 2 mM EDTA, and 5% NP-40, supplemented with protease inhibitors, including 10 μg^−1^ ml^−1^ aprotinin, 1 mM phenylmethylsulfonyl fluoride, 1 μM pepstatin, and 10 μM leupeptin) for 10 min. Then, the soluble fraction was isolated by centrifugation at 21,130 × *g* for 20 min at 4 °C. FLAG M2 beads (Sigma-Aldrich) were incubated with 1 ml cell lysate at 4 °C for 3 h. After incubation, complexes were washed five times with NP-40 lysis buffer and the bead-conjugated proteins were denatured in 2× SDS loading buffer for 15 min at 95 °C. Protein samples were then separated by SDS-PAGE and detected by immunoblotting. Uncropped blots can be found in Supplementary Figs. 11–15.

### Seahorse analysis

OCR was measured using Seahorse XF96 equipment (Seahorse Bioscience Inc.). Cells were seeded at 25,000 cells per well 24 h prior the measurement and equilibrated in a CO_2_-free incubator at 37 °C for 1 h. Analyses were performed using 1 µM oligomycin, 0.5 µM CCCP, and 1 µM rotenone plus 1 µM antimycin A as indicated.

### Flow cytometry analysis

Mitochondrial membrane potential was measured by incubating live cells in DMEM supplemented with 10 nM TMRM for 15 min, after which cells were trypsinized and resuspended in PBS. The labeled samples were analyzed using flow cytometry (BD LSR Fortessa or Cytoflex LX) and results were analyzed using SummitTM 4.3 or CyExpert software. Ten thousand events were recorded for each experiment.

For the mt-Keima-based mitophagy assay, *WT* or *Fis1 KO* HeLa cells stably expressing mt-Keima were transiently transfected with the plasmid as indicated. Cells were harvested and analyzed for flow cytometry as indicated^19^.

### Electron microscopy

Cells as treated were fixed with 2.5% glutaraldehyde in 0.2 M sodium phosphate buffer (pH 7.4), followed by PBS wash. Then, dehydration was performed in a graded series of ethanol: 30%, 50%, 70%, 95%, and 100% ethanol twice for 15 min and propylene oxide (PO) for further 15 min. Cells were resuspended in 1:1 PO/resin and embedded at 80 °C. The embedded samples were trimmed and sectioned for EM. The samples were visualized using a 120 kV Jeol electron microscope at 80 kV and images were captured using an AMT digital camera. Quantitative analysis of mitophagosome or mitochondria numbers were assessed across 20 randomly selected fields of view.

### Statistical analysis

For quantification, values were obtained from three independent experiments, shown as mean ± SD. Statistical data were processed in GraphPad Prism software or Excel. Analyses were calculated using the two-tailed unpaired Student’s *t* test, *P < 0.05, **P < 0.01, and ***P < 0.001 are considered significant.

## Supplementary information

Supplementary Information

Peer review

Supplementary Movie 1

Supplementary Data 1

## Data Availability

The data generated from this study are available from the corresponding author upon reasonable request.
