# Author Correction: STX17 dynamically regulated by Fis1 induces mitophagy via hierarchical macroautophagic mechanism

**DOI:** 10.1038/s41467-021-26559-3

**Published:** 2021-11-16

**Authors:** Hongxu Xian, Qiaoyun Yang, Lin Xiao, Han-Ming Shen, Yih-Cherng Liou

**Affiliations:** 1 Department of Biological Sciences, Faculty of Science, National University of Singapore, 14 Science Drive 4, 117543 Singapore, Singapore; 2 Department of Physiology, Yong Loo Lin School of Medicine, National University of Singapore, Singapore, 117597 Singapore; 3 NUS Graduate School for Integrative Sciences and Engineering, National University of Singapore, Singapore, Singapore

**Keywords:** Mitophagy, SNARE, Mitochondria

Correction to: *Nature Communications* 10.1038/s41467-019-10096-1, published online 03 May 2019.

In the Acknowledgements section of this article the grant numbers relating to the Ministry of Education, Singapore given for Yih-Cherng Liou were incorrectly given as R-154-000-637-511 and A15-114 and should have been MOE2017-T2-1-131 and R-154-000-A15-114. The original article has been corrected.

