# Supplementary material for: STX17 dynamically regulated by Fis1 induces mitophagy via hierarchical macroautophagic mechanism
Source: Nat Commun. 2019 May 3;10:2059. doi: 10.1038/s41467-019-10096-1 (PMC6499814; doi:10.1038/s41467-019-10096-1)
Supplement: Supplementary file 1 — Supplementary Information [file 41467_2019_10096_MOESM1_ESM.pdf]

Supplementary Information

STX17 dynamically regulated by Fis1 induces mitophagy via hierarchical macroautophagic mechanism

Xian et al.

Supplementary Movie 1. STX17 initiates mitophagosome upon Fis1 loss. *Fis1KO* HeLa cells transfected with RFP-Mito, GFP-LC3 and BFP-STX17 were imaged by structure illumination microscopy (SR-SIM) for 5 min at 30 s intervals (related to **Supplementary Figure 2e**).

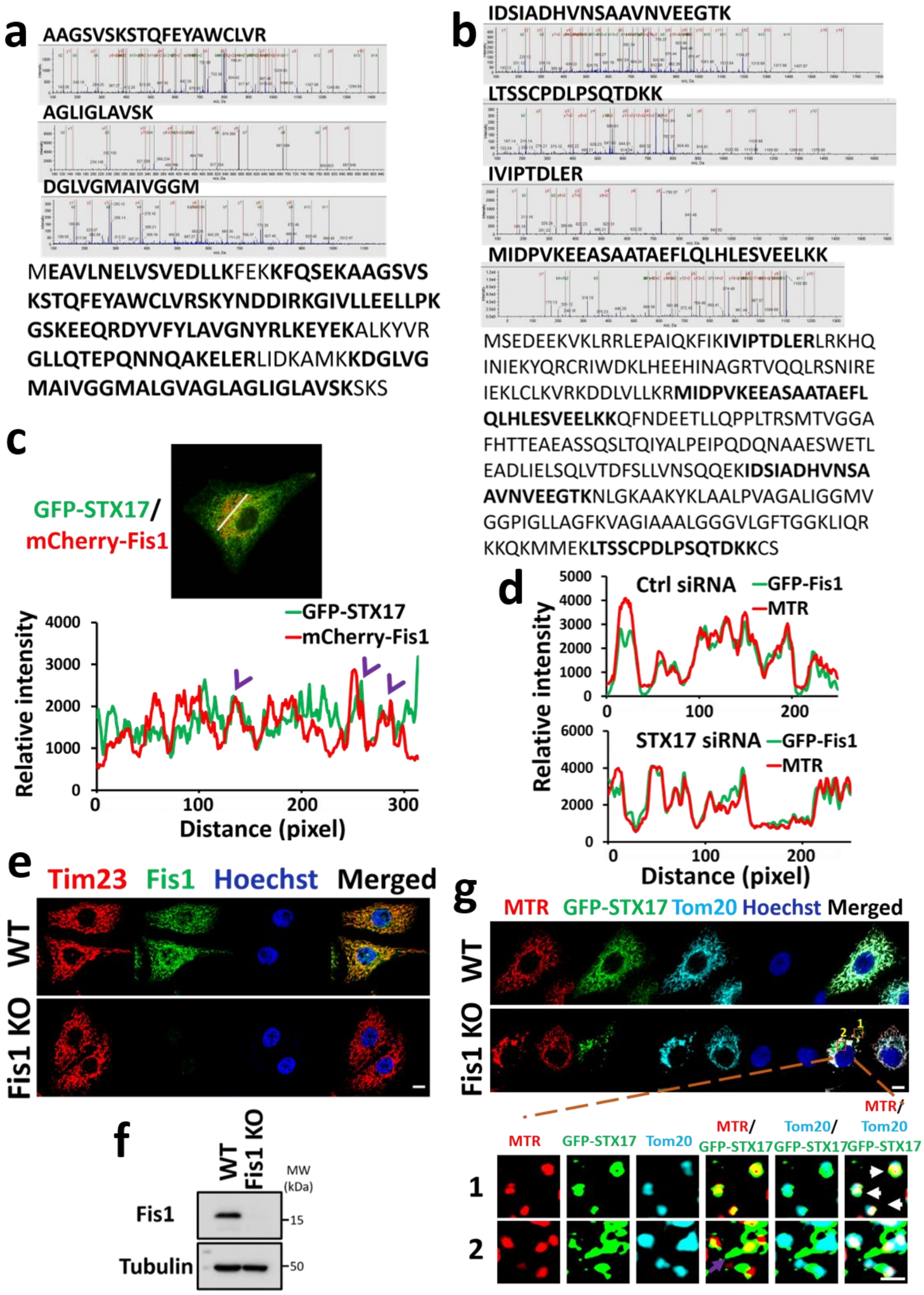

Supplementary Figure 1. The interaction between Fis1 and STX17. (**a-b**) Mass spectrometry analysis of Fis1 (**a**) and STX17 (**b**) peptides. (**c**) The co-localization of Fis1 and STX17 in **Fig. 1e** (last panel). Graph represents the relative intensity of GFP-STX17 (green) and mCherry-Fis1 (red) along the line scan. Purple arrowheads indicate the colocalization of STX17 and Fis1. (**d**) Line scans corresponding to Fig. 1**f** (upper panel). STX17 silencing does not perturb mitochondrial targeting of Fis1. (**e-f**) The validation of *Fis1 KO* HeLa cells. The single cell colony derived and expanded from *Fis1 KO* HeLa cells was verified by immunofluorescence analysis (**e**) and immunoblotting (**f**). (**g**) *WT* or *Fis1 KO* HeLa cells were transfected with GFP-tagged STX17 (green) for 24 h. Mitochondria were labelled using MitoTracker Red (MTR, red) and Tom20 (cyan). Hoechst, blue. Scale bar, 10 µm. Representative enlarged images (1 & 2) are shown. White arrows indicate the colocalization of STX17, MTR and Tom20. Un-colocalization is indicated by purple arrow.

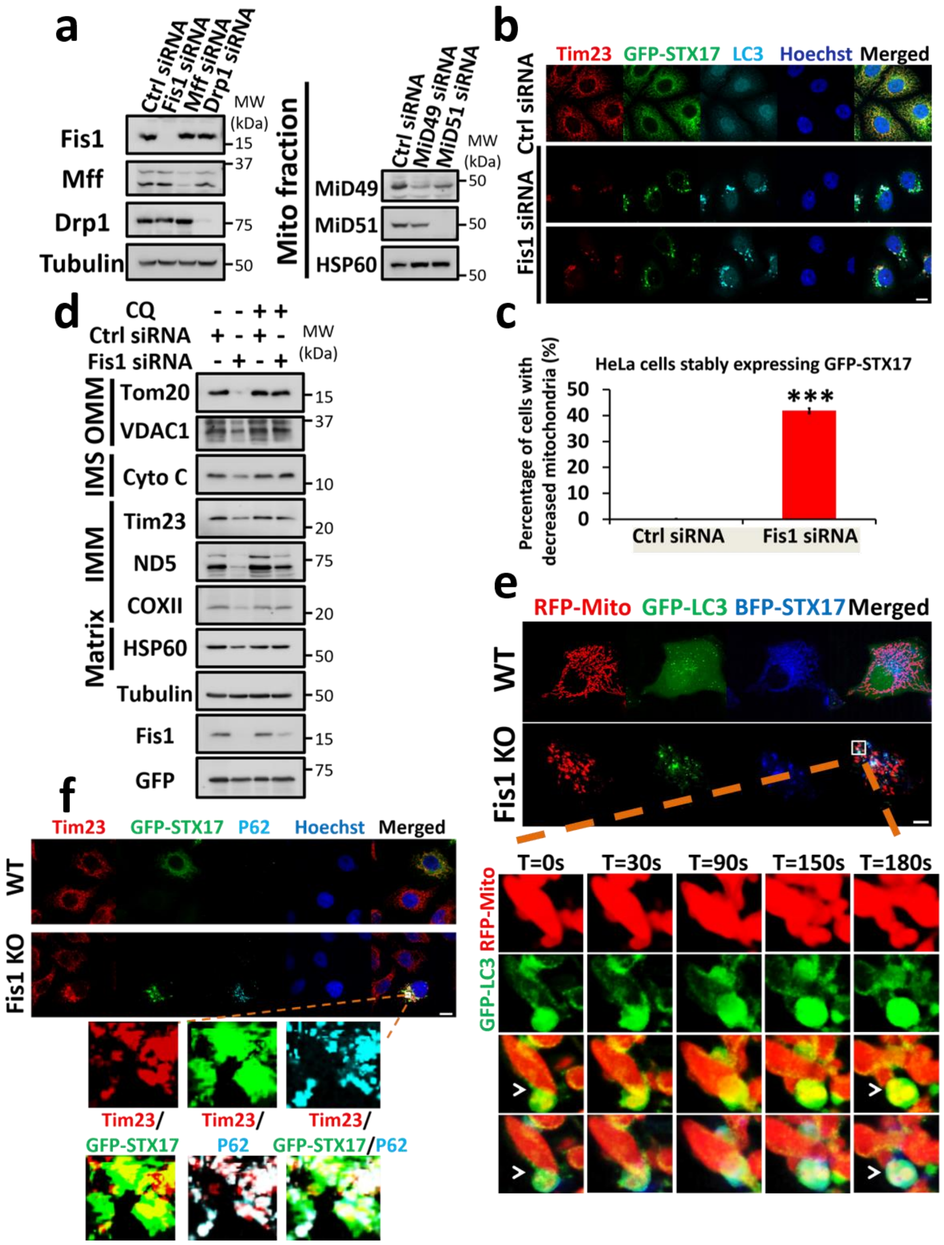

Supplementary Figure 2. Loss of Fis1 specifically triggers STX17-initiated mitophagy. (**a**) Immunoblotting of total lysates against Fis1, Mff, Drp1 and Tubulin and immunoblotting of mitochondrial extractions against MiD49, MiD51 and HSP60 are shown to verify the silencing in **Fig 2a**. (**b**) HeLa cells stably expressing GFP-STX17 were transfected with control- or *Fis1*- siRNA for 48 h. Cells were fixed and immunostained against Tim23 (red) and LC3 (cyan). Hoechst, blue. Scale bar, 10 µm. (**c**) Quantification of cells with decreased mitochondria as shown in (**b**). Error bars, SD. ***$P < 0.001$ (two-tailed Student's t test). n = 150 cells from three independent experiments. (**d**) Cells stably expressing GFP-STX17 treated as in (**b**) were cultured with or without CQ. Cells were harvested and analyzed by immunoblotting of overall mitochondrial proteins. (**e**) *WT* or *Fis1 KO* HeLa cells transfected with RFP-Mito, GFP-LC3 and BFP-STX17 were imaged by structure illumination microscopy (SR-SIM) for 5 min at 30 s intervals. Scale bar, 10 µm. The enlarged images indicate three-dimensional reconstruction. (**f**) *WT* or *Fis1 KO* HeLa cells transiently expressing GFP-STX17 were fixed and immunostained against Tim23 (red) and P62 (cyan). The colocalization of Tim23 and P62 is shown in three-dimensional reconstruction images acquired from SR-SIM. Hoechst, blue. Scale bar, 10 µm.

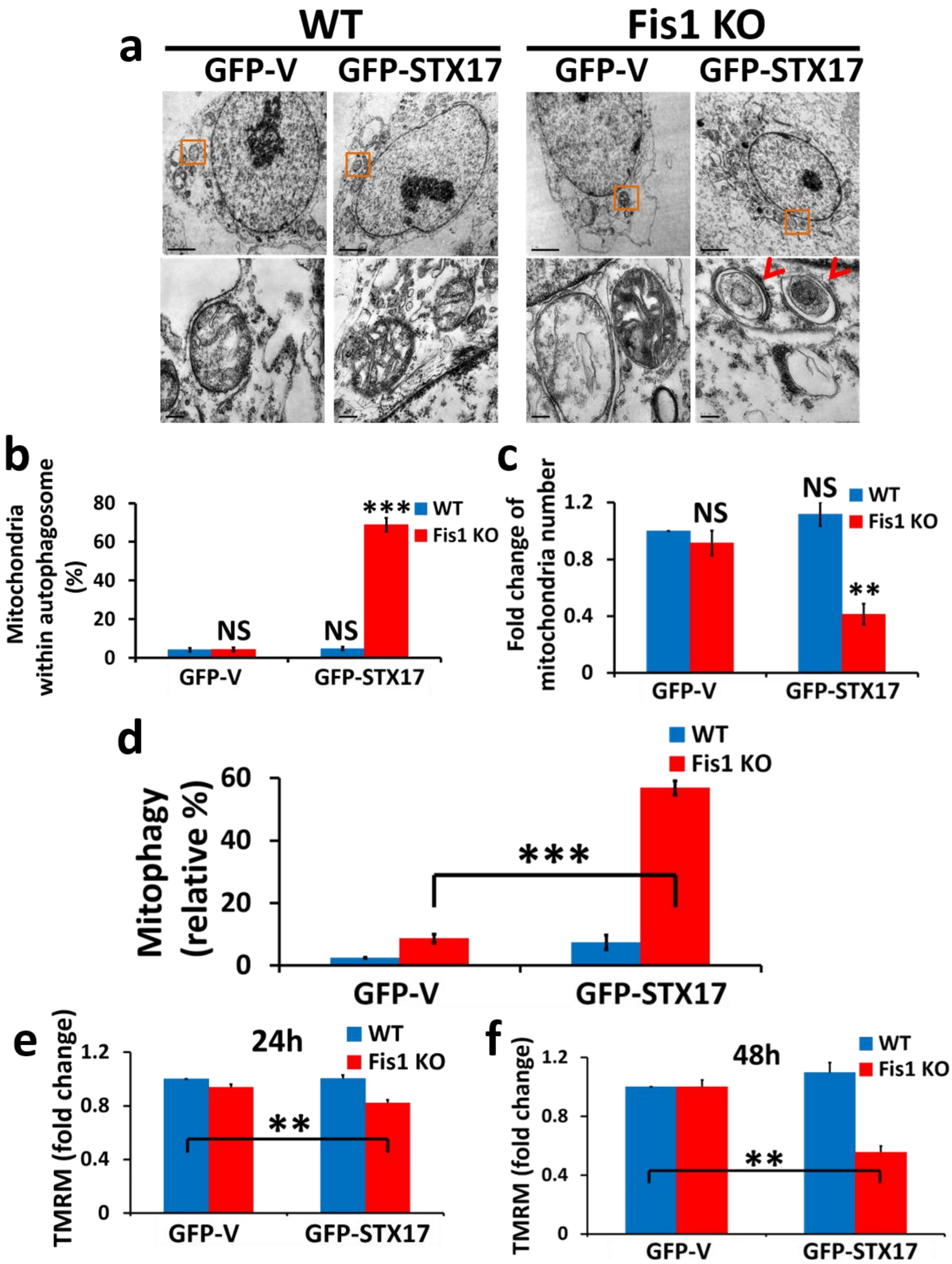

Supplementary Figure 3. Validation of mitophagy upon Fis1 loss. (**a**) *WT* or *Fis1 KO HeLa* cells transfected as indicated were analyzed by electron microscopy. Scale bars, 0.2 µm or 2 µm (enlarged). Red arrowheads indicate autophagic structures enclosing mitochondria. (**b-c**) Bar charts showing the percentage of autophagosomes containing mitochondria (**b**) or mitochondria number per area (**c**) counted from 20 randomly selected areas from three experiments treated as in (**a**). $^{**}P < 0.01$, $^{***}P < 0.001$, NS, not significant (two-tailed unpaired Student's *t* test). (**d**) Quantification of mitophagy in **Fig. 2h**. $^{***}P < 0.001$ (two-tailed unpaired Student's *t* test, n = 3). (**e-f**) Graphs indicate the normalized intensity of TMRM probed in *WT* or *Fis1 KO* HeLa cells expressing GFP-vector or GFP-STX17 for 24 h (**e**) or 48 h (**f**) respectively. $^{**}P < 0.01$ (two-tailed unpaired Student's *t* test, n = 3). Error bar, SD (**b-f**).

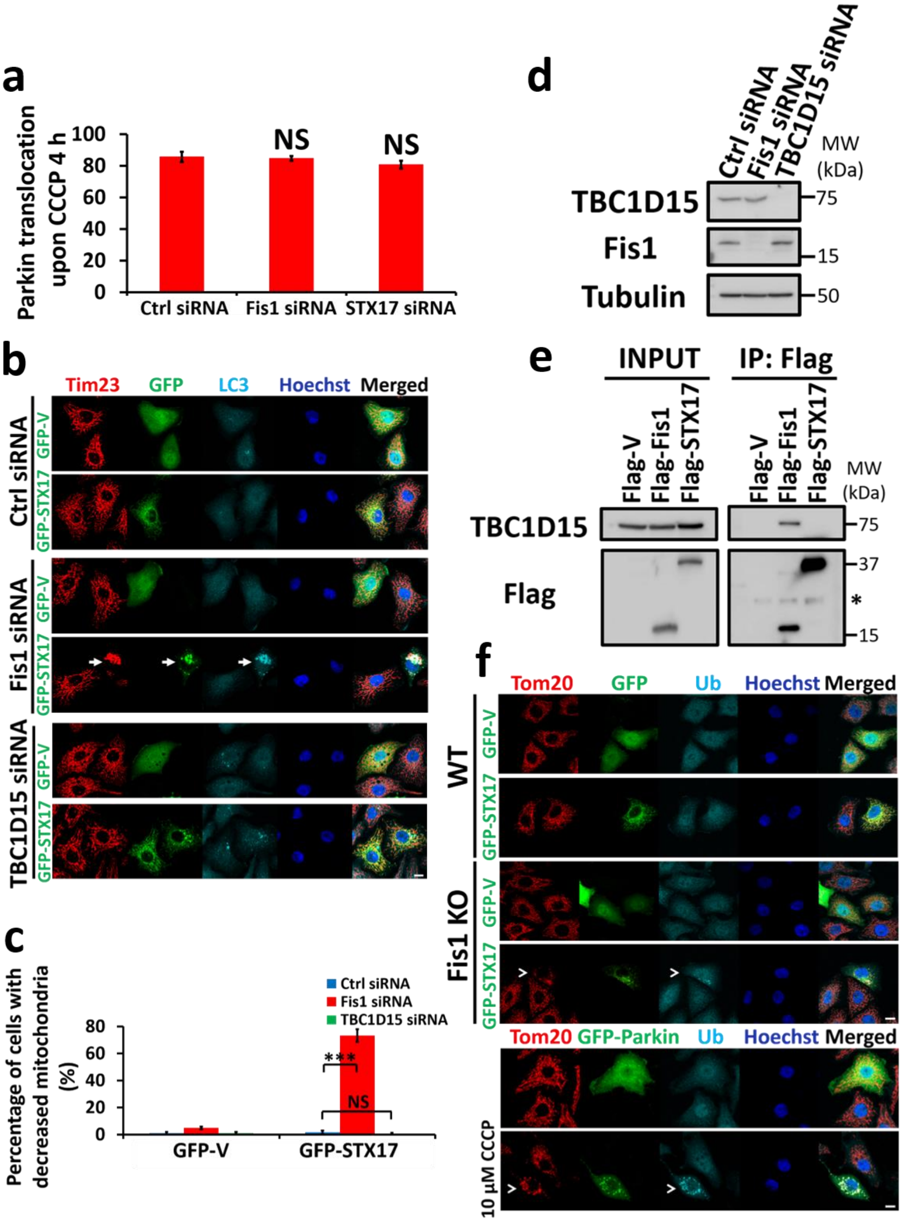

Supplementary Figure 4. Loss of TBC1D15 fails to initiate mitophagy upon STX17 OE. (**a**) Quantification of GFP-Parkin accumulation onto mitochondria in **Fig 3f**. NS, not significant (two-tailed unpaired Student's *t* test, n = 150 cells from three independent experiments). (**b**) HeLa cells treated with control-, *Fis1*- or *TBC1D15*- siRNA for 24 h were transiently transfected with GFP-tagged vector or STX17 for further 24 h. Cells were analyzed with immunofluorescence against Tim23 (red) and LC3 (cyan). Hoechst, blue. Scale bar, 10 µm. White arrow indicates mitophagic cell. (**c**) Scoring of mitophagy formation as in (**b**). ****P* < 0.001, NS, not significant (two-tailed unpaired Student's *t* test, n = 150 from three independent replicates). (**d**) The siRNA silencing in (**b**) was confirmed by immunoblotting. (**e**) Cells transfected as indicated were extracted and co-immunoprecipitated. (**f**) (Upper panel:) *WT* or *Fis1 KO* HeLa cells were transfected with GFP-vector or STX17 (green) for 24 h. Cells were fixed and immunostained for Tom20 (red) and Ub (cyan). Hoechst, blue. Scale bar, 10 µm. Arrowhead indicates mitophagic cell. (Lower panel:) HeLa cells transfected with GFP-Parkin for 24 h were treated with or without 10 µM CCCP for 4 h. Then cells were fixed and immunostained for Tom20 (red) and Ub (cyan). Hoechst, blue. Scale bar, 10 µm. Arrowhead indicates cell with Parkin translocation.

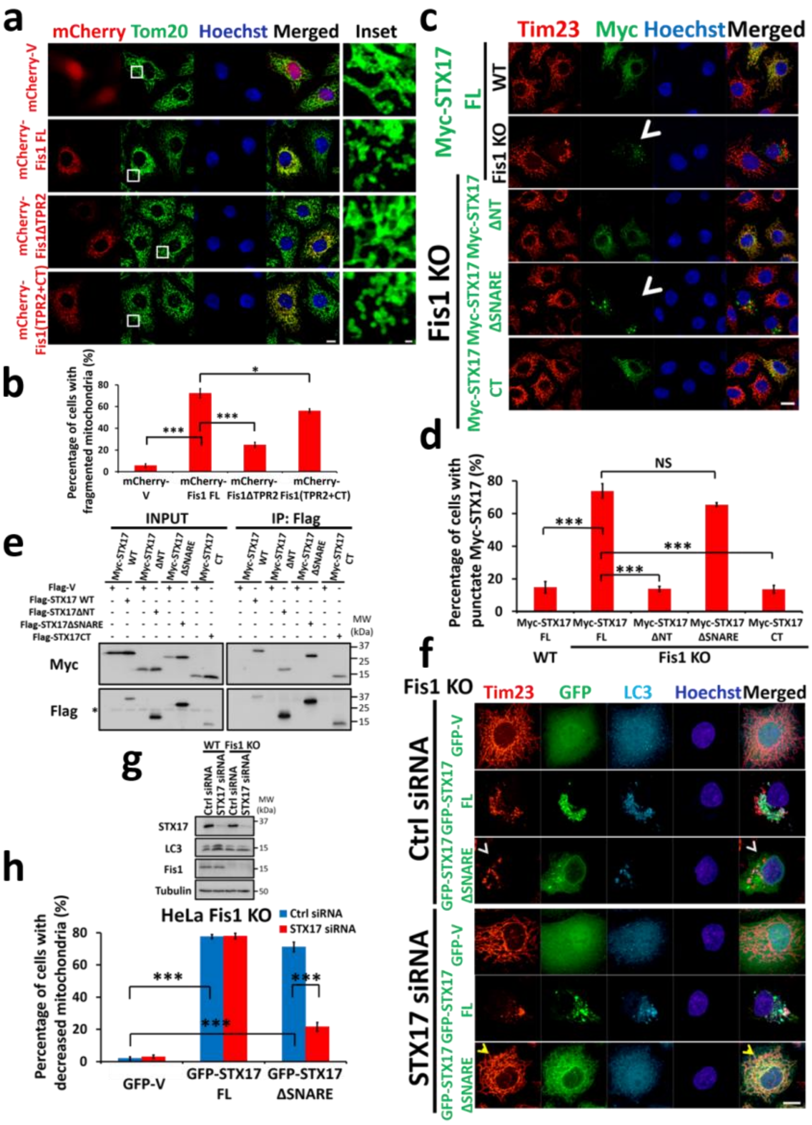

Supplementary Figure 5. STX17 oligomerizes for mitophagy, regulated by Fis1. (**a**) *Fis1 KO* HeLa cells were transfected with mCherry-tagged plasmids encoding respective truncations of Fis1. Mitochondria were highlighted with Tom20 (green). Hoechst, blue. Scale bar, 10 μm. Magnified images indicate mitochondrial morphology. Scale bar, 2 μm (Inset). (**b**) Quantitative analysis of mitochondrial fission induced by Fis1 truncations as shown in (**a**). **P* < 0.05, ****P* < 0.001 (two-tailed unpaired Student's *t* test, n = 150 from three independent replicates). (**c**) *WT* or *Fis1 KO* HeLa cells were transfected as indicated for 24 h. Cells were fixed and immunostained for Tim23 (red) and Myc (green). Hoechst, blue. Scale bar, 10 μm. Puncta formation is indicated as arrowhead. (**d**) Scoring of STX17 puncta formation as in (**c**). ****P* < 0.001, NS, not significant (two-tailed unpaired Student's *t* test. n = 150 from three replicates). (**e**) *Fis1 KO* HeLa cells expressing the indicated plasmids were extracted and co-immunoprecipitated. (**f**) *Fis1 KO* HeLa cells were transfected with the indicated siRNA for 24 h and then overexpressed with GFP-tagged vector or siRNA-resistant STX17 FL or STX17 ΔSNARE. Cells were fixed and immunostained with Tim23 (red) and LC3 (cyan). Hoechst, blue. Scale bar, 10 μm. White arrowhead indicates mitophagic cell, while yellow arrowhead represents non-mitophagic cell. (**g**) Verification of *STX17* siRNA silencing by immunoblotting. (**h**) Quantification of (**f**). ****P* < 0.001 (two-tailed unpaired Student's *t* test, n = 150 from three independent replicates). Data are mean ± SD (**b**, **d**, **h**).

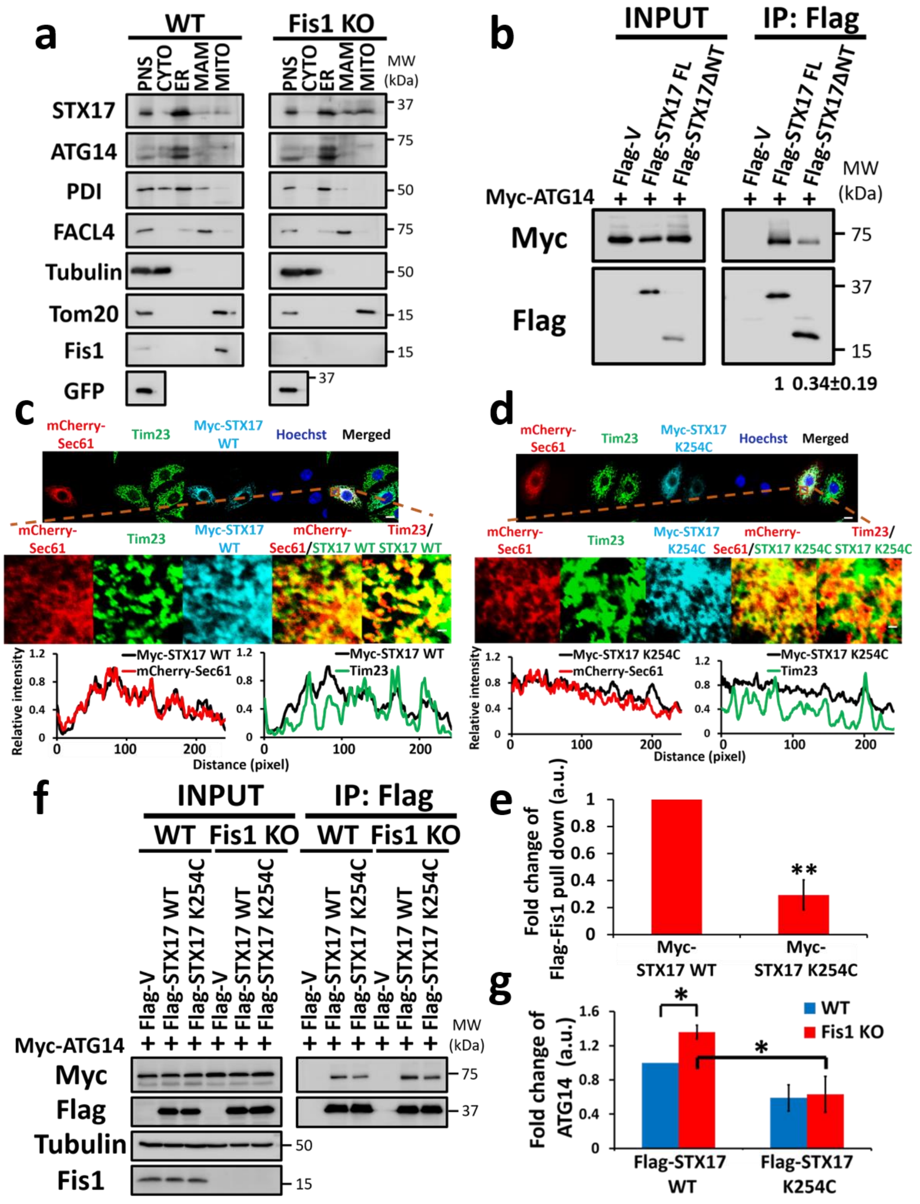

Supplementary Figure 6. Mitochondria-targeted STX17 induces mitophagy. (**a**) Immunoblotting of subcellular fractions from *WT* or *Fis1 KO* HeLa cells transiently expressing GFP-tagged vector for 24 h. PNS, post-nuclear supernatant; CYTO, cytosol; ER, endoplasmic reticulum; MAM, mitochondria-associated membranes; MITO, mitochondria. (**b**) HEK293T cells were transfected with the indicated plasmids for 24 h. Cells were extracted and immunoprecipitated, followed by immunoblotting. (**c-d**) HeLa cells transiently transfected with mCherry-tagged Sec61 (red, ER marker) and Myc-tagged STX17 WT (**c**) or K254C (**d**) for 24 h were fixed and immunostained against Tim23 (green) and Myc (cyan). Hoechst, blue. Scale bar, 10 μm. Enlarged images and line scans indicate the colocalization of STX17 WT or K254C on ER (Sec61) or mitochondria (Tim23) respectively. (**e**) Quantification of Fis1 binding to STX17 WT or STX17 K254C from **Fig 5i** is shown. $^{**}P < 0.01$ (two-tailed unpaired Student's *t* test, n = 3). (**f**) Flag-tagged plasmid encoding STX17 WT or K254C mutant was co-transfected with Myc-ATG14L into *WT* or *Fis1 KO* HeLa cells for 24 h. Cells were extracted and immunoprecipitated with anti-Flag beads, followed by immunoblotting. (**g**) Quantification of ATG14L binding to STX17 WT or STX17 K254C is shown. $^{*}P < 0.05$ (two-tailed unpaired Student's *t* test, n =3). Error bars indicate SD (**e** and **g**).

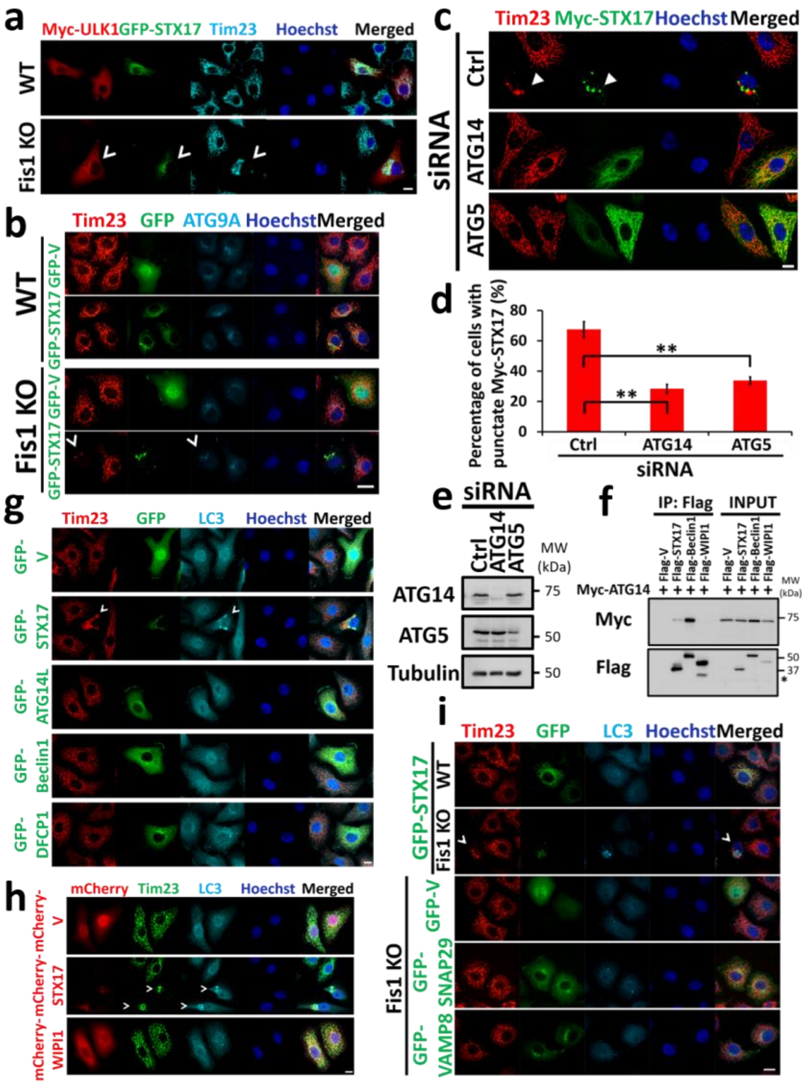

Supplementary Figure 7. Canonical autophagy proteins fail to induce mitophagy. (**a**) *WT* or *Fis1 KO* HeLa cells were transiently co-transfected with Myc-tagged ULK1 and GFP-tagged STX17. After 24 h, cells were fixed, immunostained with anti-Myc (red) and anti-Tim23 (cyan) antibodies, and further imaged by confocal microscopy. Hoechst, blue. Scale bar, 10 µm. Arrowhead indicates mitophagic cell. (**b**) *WT* or *Fis1 KO* HeLa cells were transfected with GFP-tagged STX17 for 24 h. Mitophagy was monitored by fixation and immunostaining for Tim23 (red) and ATG9A (cyan). Hoechst, blue. Scale bar, 10 µm. Arrowhead indicates mitophagic cell. (**c**) *Fis1 KO* HeLa cells were transfected with siRNA as indicated for 24 h. Myc-tagged STX17 was introduced into cells for further 24 h. Cells were fixed and immunostained with Tim23 (red) and Myc (green) antibodies. Hoechst, blue. Scale bar, 10 µm. Triangle indicates mitophagic cell, with STX17 puncta. (**d**) Scoring of STX17 puncta formation as in (**c**). Bars represent mean ± SD. $^{**}P < 0.01$ (two-tailed unpaired Student's *t* test, n =150 from three independent replicates). (**e**) The siRNA silencing in (**c-d**) was verified by immunoblotting. (**f**) HEK293T cells were transiently co-transfected with Flag-tagged plasmids encoding the indicated proteins and Myc-tagged ATG14 for 24 h. Cells were collected and co-immunoprecipitated with anti-Flag beads followed by immunoblotting. (**g-i**) *WT* or *Fis1 KO* HeLa cells as indicated were transfected with respective plasmid for 24 h before fixation and immunostaining with anti-Tim23 (green or red) and anti-LC3 (cyan) antibodies. Hoechst, blue. Scale bar, 10 µm. Arrowheads indicate mitophagic cells.

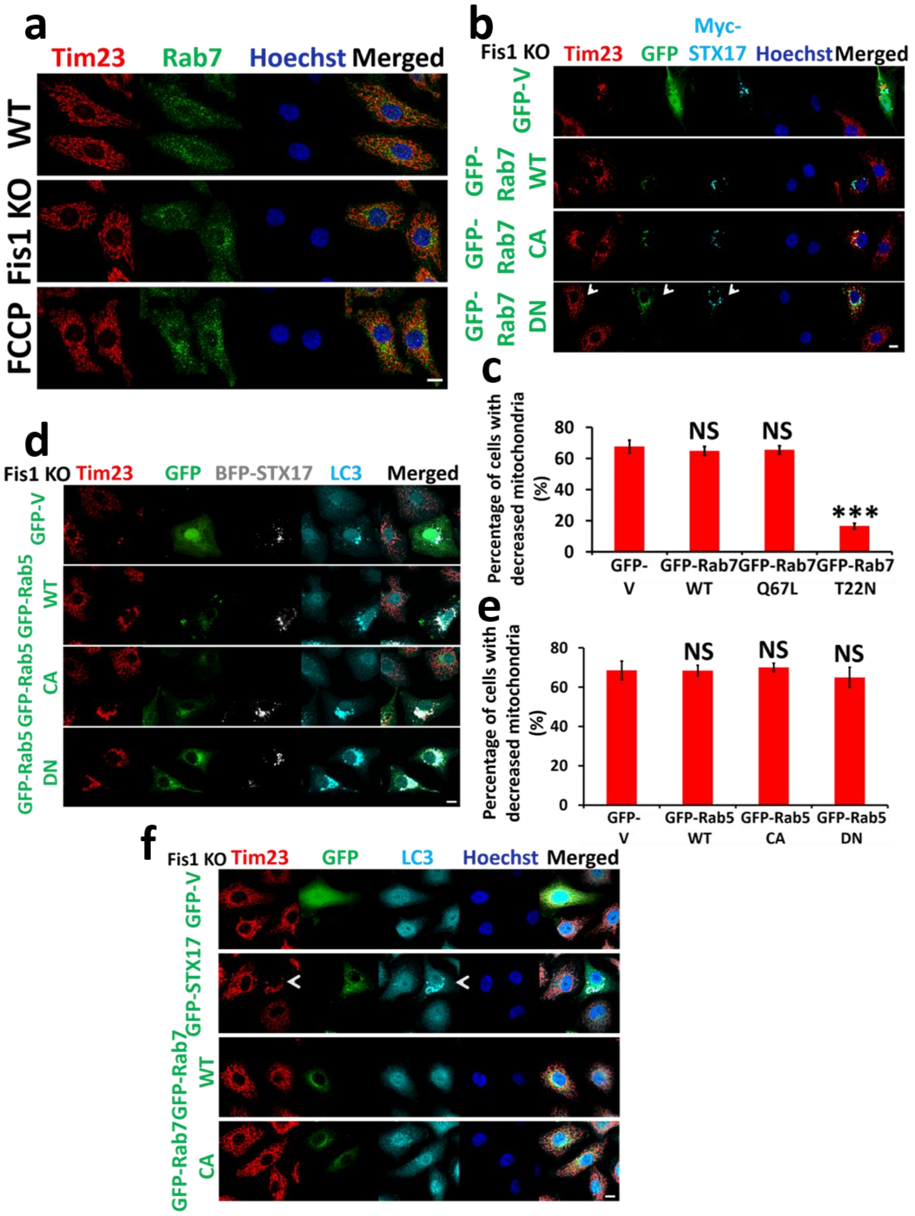

Supplementary Figure 8. Rab7 is indispensable for STX17-regulated mitophagy. (**a**) *WT*, *Fis1 KO* HeLa cells or *WT* HeLa cells treated with 10 μM FCCP for 6 h were fixed and hereafter immunostained for Tim23 (red) and Rab7 (green). Hoechst, blue. Scale bar, 10 μm. (**b**) *Fis1 KO* HeLa cells were transiently transfected with GFP-tagged plasmids encoding Rab7 WT or mutant (green) for 24 h. Cells were then transfected with Myc-STX17 for further 24 h. Cells were fixed and immunostained for Tim23 (red) and Myc (cyan). Hoechst, blue. Scale bar, 10 μm. Arrowhead indicates mitophagic cell. (**c**) Quantification of (**b**). NS, not significant, ***$P < 0.001$ (two-tailed unpaired Student's *t* test, n = 150 from three independent replicates). (**d**) *Fis1 KO* HeLa cells were transfected with GFP-tagged plasmids encoding Rab5 WT or mutant for 24 h. Hereafter cells were transfected with BFP-STX17 (gray) for further 24 h, followed by fixation and immunostaining for Tim23 (red) and LC3 (cyan). Hoechst, blue. Scale bar, 10 μm. (**e**) Quantification of (**d**). NS, not significant (two-tailed unpaired Student's *t* test, n = 150 from three independent replicates). Data are mean ± SD (**c** and **e**). (**f**) *Fis1 KO* HeLa cells were transiently transfected with GFP-tagged plasmid as indicated for 24 h. Cells were fixed and immunostained against Tim23 (red) and LC3 (cyan) antibodies. Hoechst, blue. Scale bar, 10 μm. Arrowhead indicates mitophagic cell.

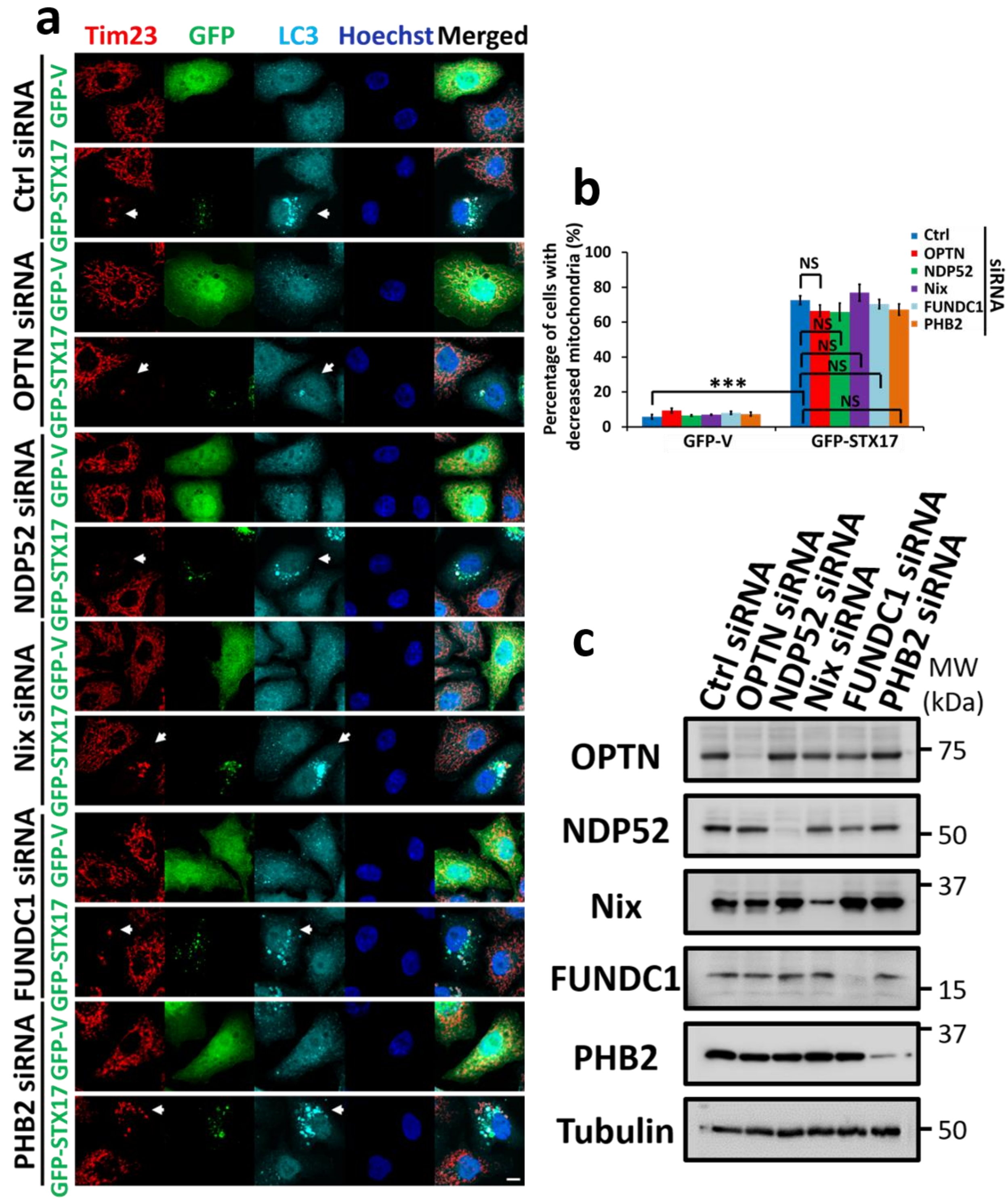

Supplementary Figure 9. Mitophagy receptors are not involved. (**a**) *Fis1 KO* HeLa cells were transfected with the indicated siRNA for 24 h. Cells were then transfected with GFP-tagged vector or STX17 for further 24 h, followed by fixation and immunostaining for Tim23 (red) and LC3 (cyan). Hoechst, blue. Scale bar, 10 μm. Arrowheads indicate mitophagic cells. (**b**) Quantitative analysis of mitophagic cells as in (**a**). Error bars represent SD. ***$P < 0.001$, NS, not significant (two-tailed unpaired Student's *t* test, n = 150 from three independent replicates). (**c**) Silencing efficiency of siRNAs used in (**a**) was verified by immunoblotting.

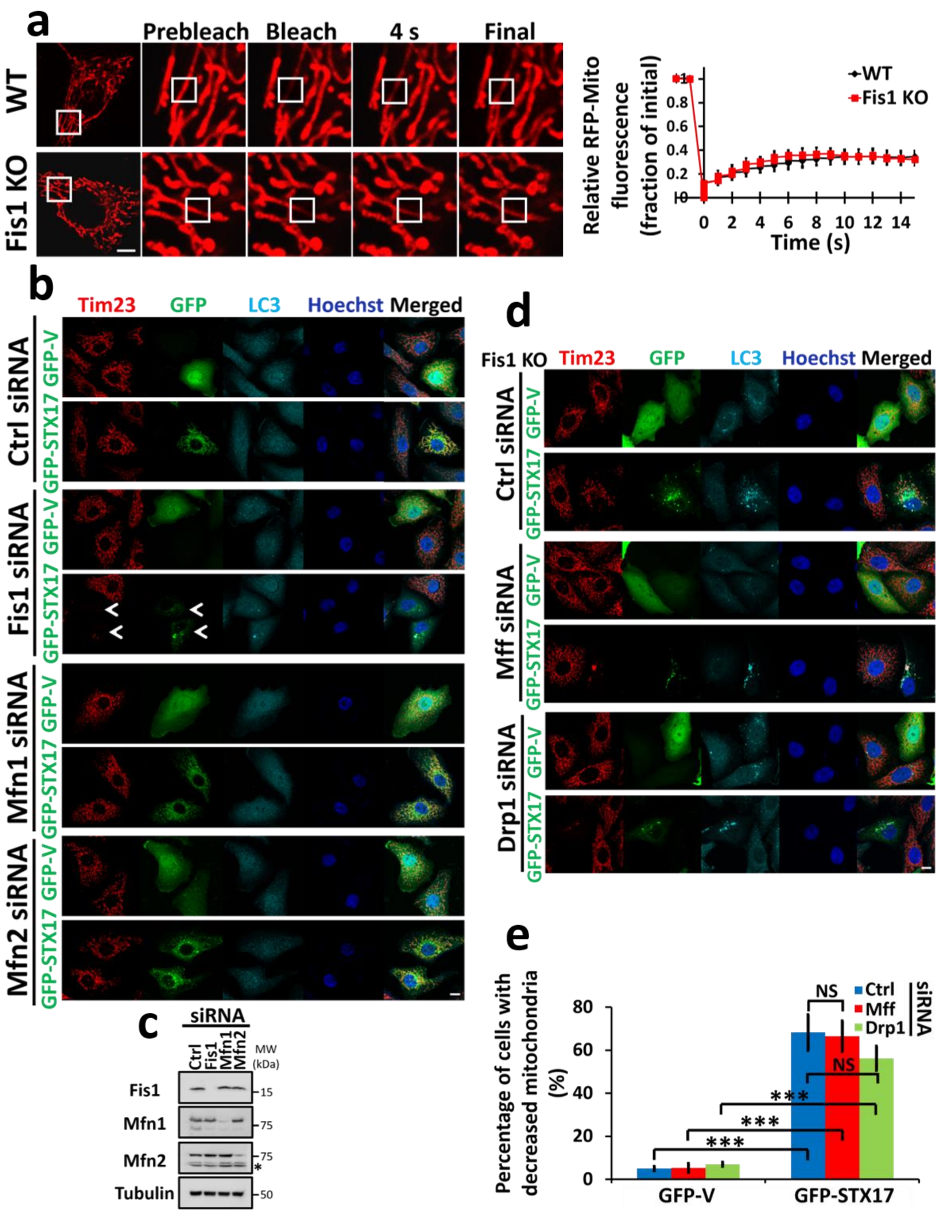

Supplementary Figure 10. The effect of mitochondrial dynamics on mitophagy. (**a**) FRAP analysis of *WT* and *Fis1 KO* HeLa cells. *WT* or *Fis1 KO* HeLa cells transfected with RFP-Mito plasmid were applied with FRAP analysis. The images were acquired in 0.5 s intervals. A 2 x 2 μm^2 square region of interest (ROI) was placed on the mitochondrial fibre and photobleached with a 561 nm laser. Scale bar, 10 μm. Normalized recovery curves of FRAP assay are shown. Data were collected from 15 ROIs in 15 cells from three independent experiments. (**b**) HeLa cells were silenced with the indicated siRNA for 24 h. Then cells were transfected with GFP-tagged vector or STX17 for further 24 h, followed by fixation and immunostaining against Tim23 (red) and LC3 (cyan). Hoechst, blue. Scale bar, 10 μm. Arrowhead indicates mitophagic cell. (**c**) Silencing efficiency of siRNAs used in (**b**) was verified by immunoblotting. (**d**) *Fis1 KO* HeLa cells were transfected with the indicated siRNA for 24 h. Cells were transfected with GFP-tagged vector or STX17 for further 24 h. Cells were fixed and immunostained for Tim23 (red) and LC3 (cyan). Hoechst, blue. Scale bar, 10 μm. (**e**) Quantitative analysis of mitophagic cells in (**d**). ***P < 0.001, NS, not significant (two-tailed unpaired Student's t test, n = 150 from three independent replicates). Error bars represent SD (**a** and **e**).

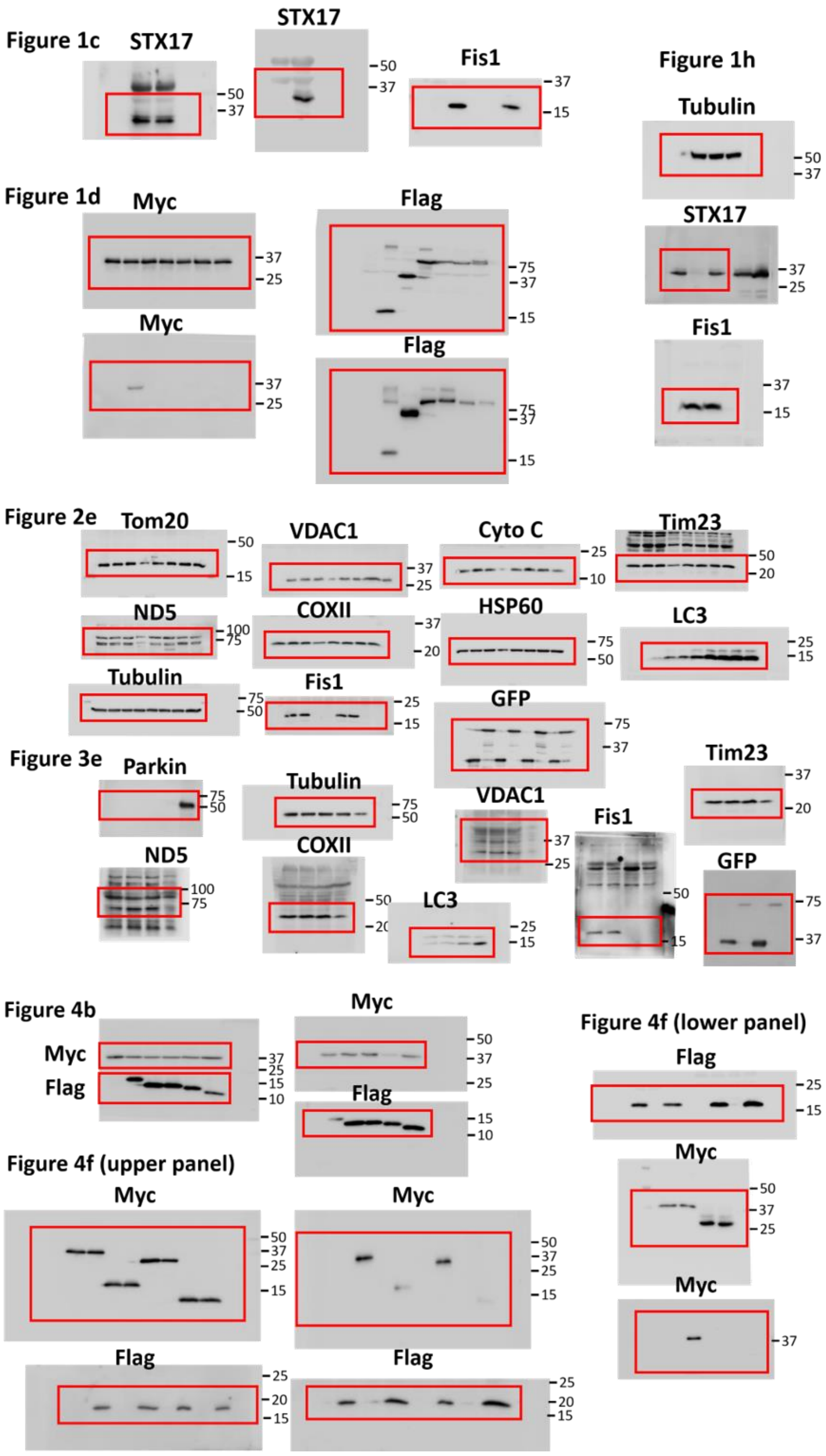

Supplementary Figure 11. Full scan for blots of Fig. 1-4.

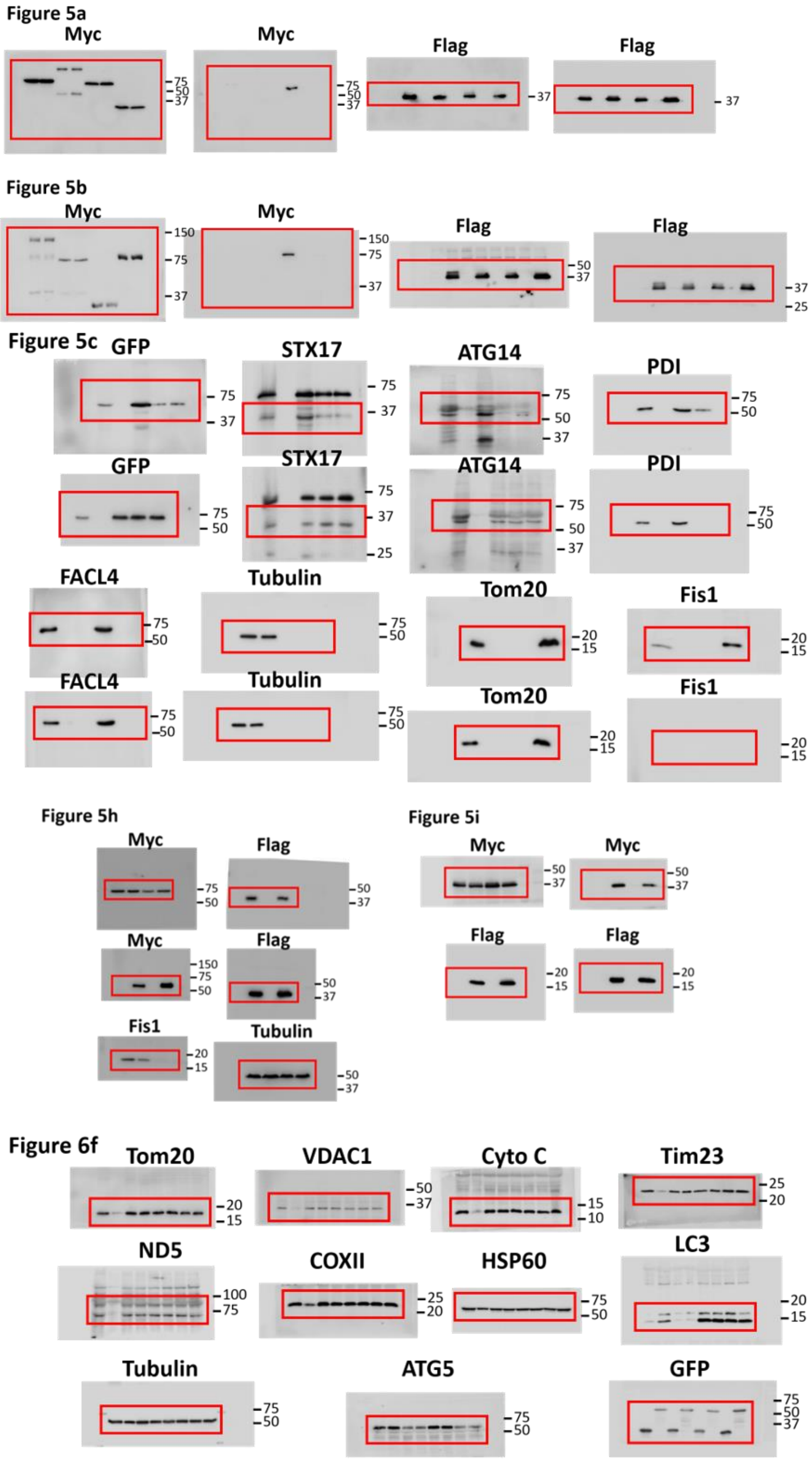

Supplementary Figure 12. Full scan for blots of Fig. 5a-6f.

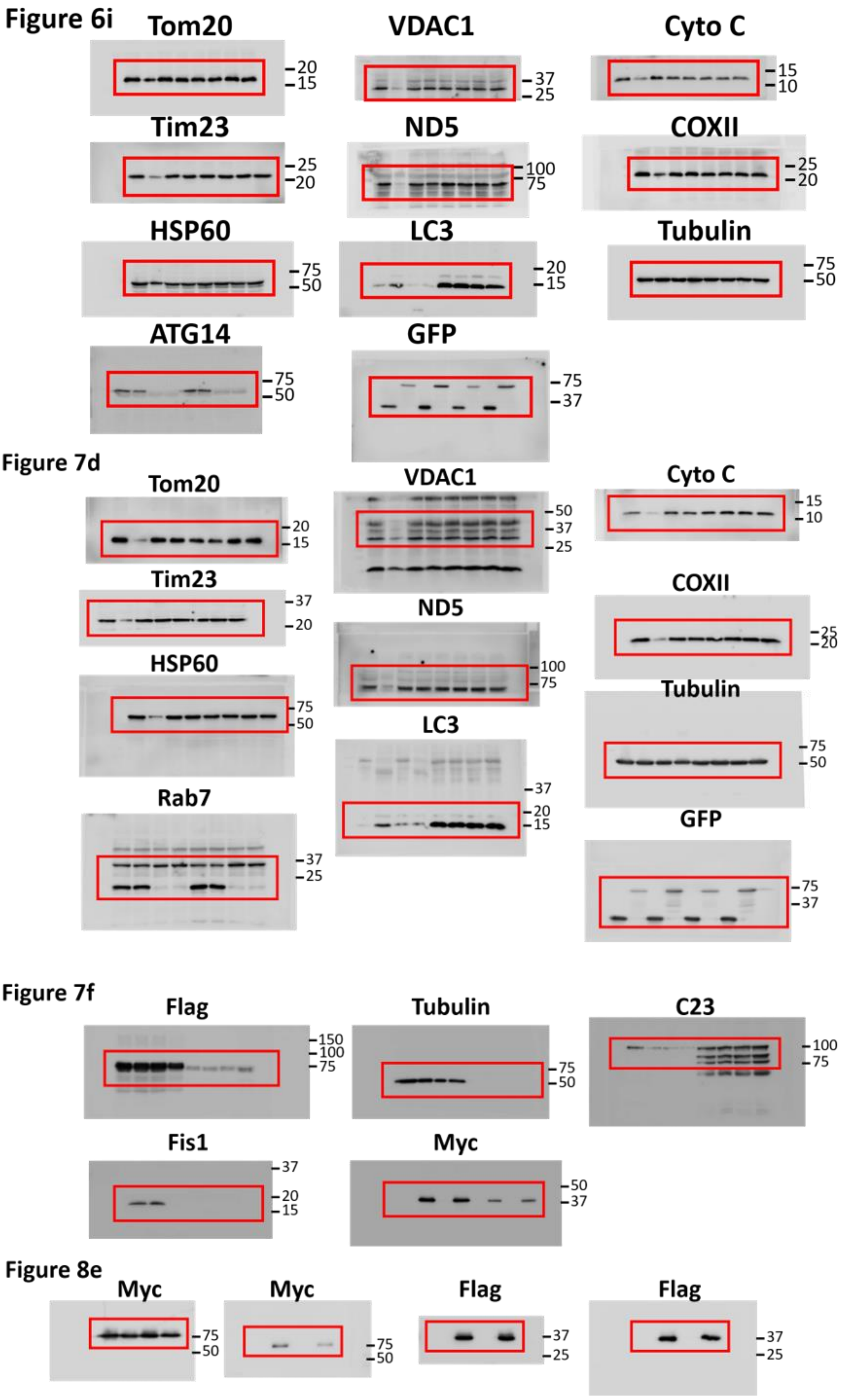

Supplementary Figure 13. Full scan for blots of Fig. 6i-8e.

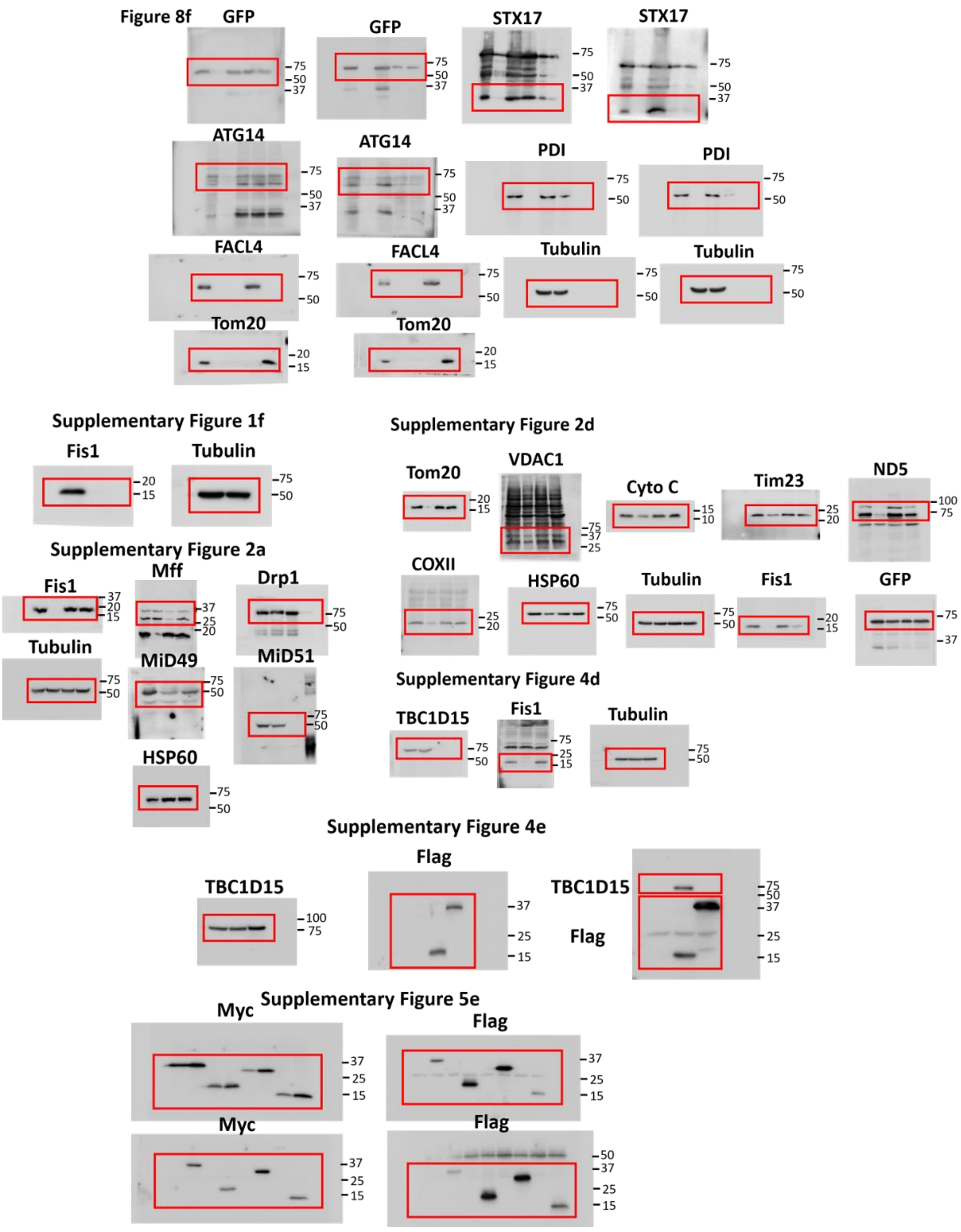

Supplementary Figure 14. Full scan for blots of Fig. 8f and Supplementary Figure 1f-5e.

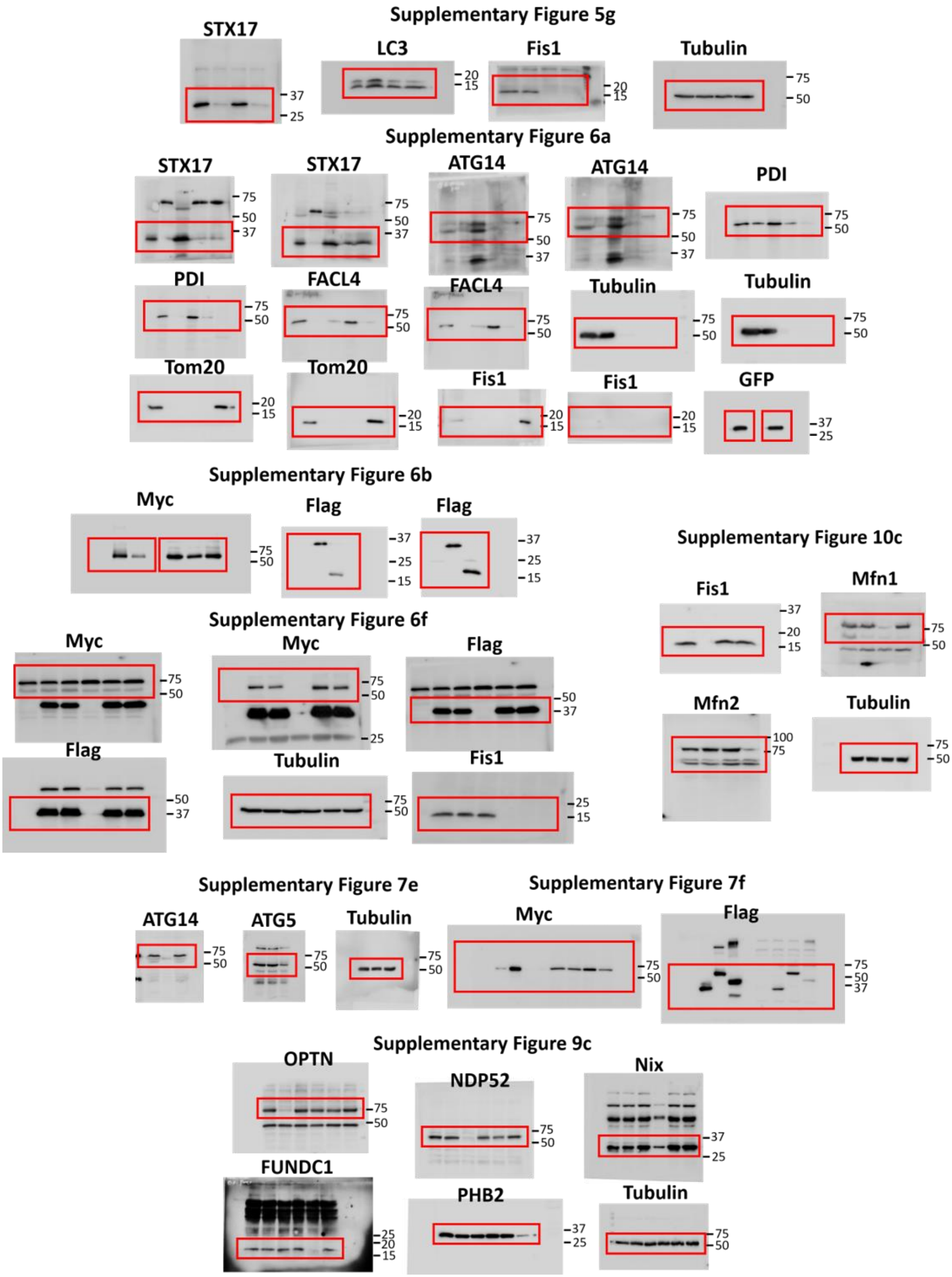

Supplementary Figure 15. Full scan for blots of Supplementary Figure 5g-9c.

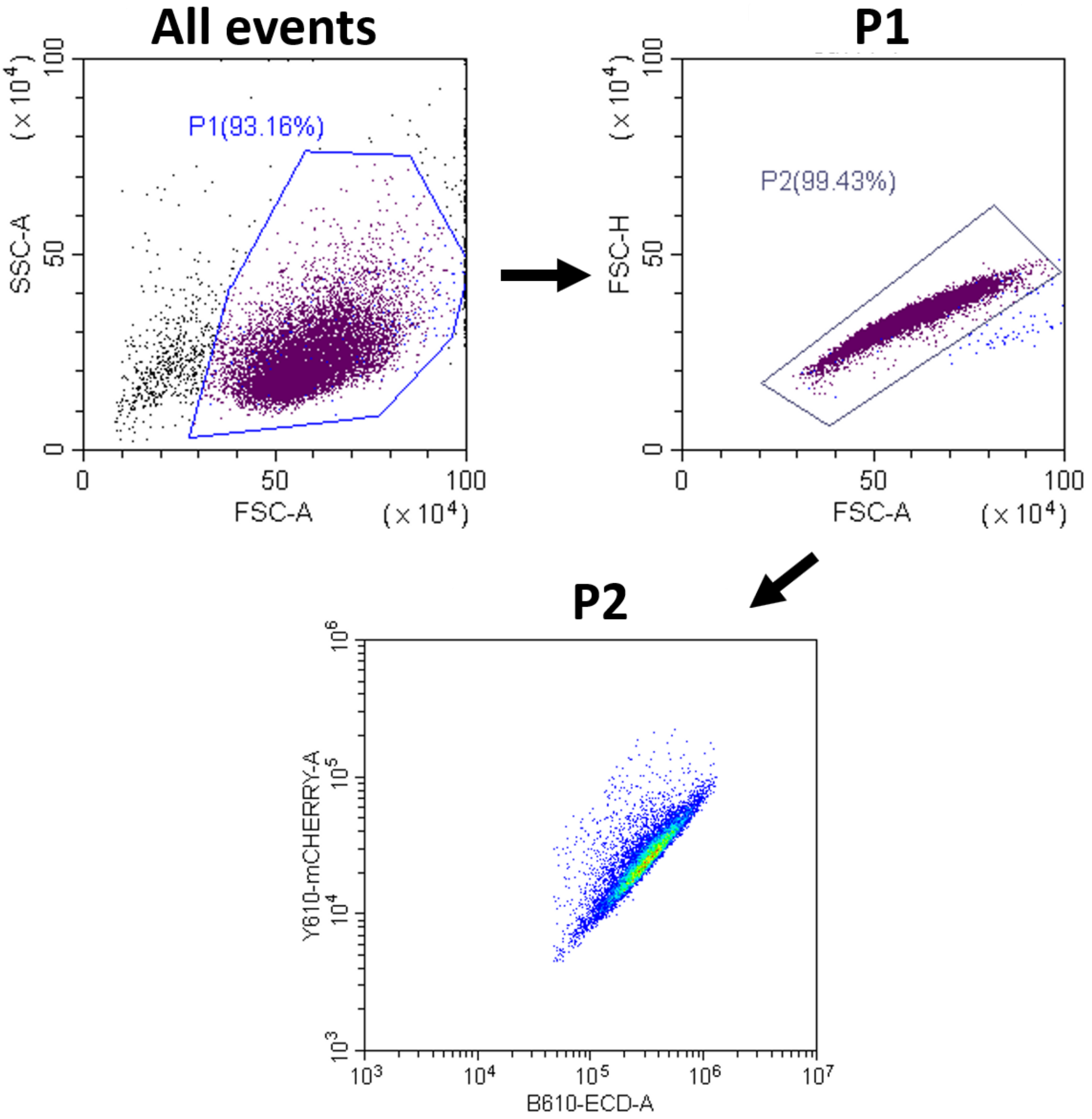

Supplementary Figure 16. Gating strategy for flow cytometry analysis. Cells were first gated for live cells (SSC-A vs. FSC-A) in P1 population and further gated as singlets (FSC-H vs. FSC-A) in P2 population. Single live cells were analyzed for the uptake of specific markers (In this example, neutral and acidic signals of mt-Keima were analyzed).

Name of primer	Vector	Sequence (5’-3’)
STX17	pXJ40	(F) CGGGATCCATGTCTGAAGATGAAGAAAAAG (R) CGCTCGAGTTAACTGCATTTCTTGTCAG
Beclin 1	pXJ40	(F) CGGGATCCATGGAAGGGTCTAAGACGTCC (R) GGGGTACCTCATTTGTTATAAAATTGTGAGGACA
ATG14	pXJ40	(F) ATAAGAATGCGGCCGCATGGCGTCTCCCAGTGGGAAG (R) GGGGTACCTTAACGGTGTCCAGTGTAAGC
WIPI1	pXJ40	(F) CCGCTCGAGATGGAGGCCGAGGCCGCGGAC (R) GGGGTACCTCATGACTGCTTCGTTTTGCCCTTC
DFCP1 (Mus musculus)	pXJ40	(F) CGGGATCCATGAGTGCCCAGACTTCCCTAGCAG (R) CCGCTCGAGTTAAAGGTCACCGGGCTTTTTATTGC
ATG5 (Mus musculus)	pXJ40	(F) CGGGATCCATGACAGATGACAAAGATGTGCTTC

		(R) CCGCTCGAGTCAATCTGTTGGCTGTGGGATG
STX17ΔNT	pXJ40	(F) CGGGATCCATGATTCCTCAAGATCAAAATGCTGCAG<br>(R) CGCTCGAGTTAACTGCATTTCTTGTCAG
STX17ΔSNARE	pXJ40	(F) CAGATATATGCCTTACCTGAAGCAAAATACAAGCTGGCAGCTC<br>(R) GAGCTGCCAGCTTGTATTTTGCTTCAGGTAAGGCATATATCTG
STX17ΔCT	pXJ40	(F) CGGGATCCATGTCTGAAGATGAAGAAGAAAAAG<br>(R) CCGCTCGAGTTAAGCCTTCCCTAAGTTTTTGGTTCCC
STX17 CT	pXJ40	(F) CGGGATCCAAGGCTGCAAAATACAAGCTGGC<br>(R) CGCTCGAGTTAACTGCATTTCTTGTCAG
STX17 (STX17 siRNA resistant)	pXJ40	(F) TGAGAAACTTTGTTTGAAAGTCCGGAAAGACGACCTAGTACTTCTGAAGAGAATG<br>(R) CATTCTCTTCAGAAGTACTAGGTCGTCTTTCCGGACTTTCAAACAAAGTTTCTCA

TFEB	c-Flag	(F)<br>CCCAAGCTTATGGCGTCACGCATAGGGTTGCGC<br>(R)<br>GGGGTACCTCACAGCACATCGCCCTCCTCCAT
Fis1Δα1	pXJ40	(F)<br>CGGGATCCGGCTCGGTGTCCAAGAGCACGCAG<br>(R)<br>GGGGTACCTCAGGATTTGGACTTGGACACAG
Fis1ΔTPR1	pXJ40	(F)<br>CAGGCTCGGTGTCCCTGCCCAAAGGGAG<br>(R)<br>CTCCCTTTGGGCAGGGACACCGAGCCTG
Fis1ΔTPR2	pXJ40	(F)<br>CTGCTGCCCAAAGGGCCCCAGAACAACCAG<br>(R)<br>CTGGTTGTTCTGGGGCCCTTTGGGCAGCAG
Fis1<br>(TPR2+CT)	pXJ40	(F)<br>CGGGATCCATGAAGAGCACGCAGTTTGAGTAC<br>GCC<br>(R)<br>GGGGTACCTCACAGCTCCTCGAGCAGCACGATG<br>C
ATG16L	pXJ40	(F)<br>AAGCTTATGTCGTCGGGCCTCCGCGCCGCTG<br>(R)<br>GCGGCCGCTCAGTACTGTGCCCACAGCACAGC
ULK1	pXJ40	(F)

(Mus musculus)		CCCAAGCTTATGGAGCCGGGCCGCGGCGGC<br>(R)<br>CCGCTCGAGTCAGGCATAGACACCACTCAGC
Rab7	pXJ40	(F)<br>CGGGATCCATGACCTCTAGGAAGAAAGTGTTGC<br>(R)<br>CCGCTCGAGTCAGCAACTGCAGCTTTCTGCCG
STX17 K254C	pXJ40	(F)<br>GCCTCCTTGCAGGCTTCTGCGTGGCAGGAATTG<br>CAGC<br>(R)<br>GCTGCAATTCCTGCCACGCAGAAGCCTGCAAGG<br>AGGC

Supplementary Table 1. Primers for cloning.
