# Supplementary material for: STX17 dynamically regulated by Fis1 induces mitophagy via hierarchical macroautophagic mechanism
Source: Nat Commun. 2019 May 3;10:2059. doi: 10.1038/s41467-019-10096-1 (PMC6499814; doi:10.1038/s41467-019-10096-1)
Supplement: Supplementary file 4 — Supplementary Data 1 [file 41467_2019_10096_MOESM4_ESM.docx]

| Accession | Name | Peptides (95%) | Unused | %Cov(95) |
| --- | --- | --- | --- | --- |
| sp\|Q9Y3D6\|  FIS1_HUMAN | Mitochondrial fission 1 protein OS=Homo sapiens GN=FIS1 PE=1 SV=2  Protein sequence coverage:  M**EAVLNELVSVEDLLK**FEK**KFQSEKAAGSVSKSTQFEYAWCLVRSKYNDDIRKGIVLLEELLPKGSKEEQRDYVFYLAVGNYRLKEYEK**ALKYVR**GLLQTEPQNNQAKELER**LIDKAMK**KDGLVGMAIVGGMALGVAGLAGLIGLAVSK**SKS | 79 | 50.85 | 86.8 |
| sp\|P56962\|  STX17_HUMAN | Syntaxin-17 OS=Homo sapiens GN=STX17 PE=1 SV=2  Protein sequence coverage:  MSEDEEKVKLRRLEPAIQKFIK**IVIPTDLER**LRKHQINIEKYQRCRIWDKLHEEHINAGRTVQQLRSNIREIEKLCLKVRKDDLVLLKR**MIDPVKEEASAATAEFLQLHLESVEELKK**QFNDEETLLQPPLTRSMTVGGAFHTTEAEASSQSLTQIYALPEIPQDQNAAESWETLEADLIELSQLVTDFSLLVNSQQEK**IDSIADHVNSAAVNVEEGTK**NLGKAAKYKLAALPVAGALIGGMVGGPIGLLAGFKVAGIAAALGGGVLGFTGGKLIQRKKQKMMEK**LTSSCPDLPSQTDKK** | 4 | 4.43 | 11.6 |

**Supplementary Table 1. Related to Fig. 1b.**
