# Supplementary material for: STX17 dynamically regulated by Fis1 induces mitophagy via hierarchical macroautophagic mechanism
Source: Nat Commun. 2019 May 3;10:2059. doi: 10.1038/s41467-019-10096-1 (PMC6499814; doi:10.1038/s41467-019-10096-1)
Supplement: Supplementary file 2 — Peer review [file 41467_2019_10096_MOESM2_ESM.pdf]

Reviewers' comments:

Reviewer #1 (Remarks to the Author):

In this manuscript, the authors identified that Fis1 interacts with STX17. Then, they found that overexpression of STX17 in Fis1 KO/KD cells induce mitophagy. They further studied the specific domain required for Fis1-STX17 interaction and the role of the domains on mitophagy. As previously reported, they showed that STX17 interacts with Atg14 and localizes on MAM. As expected again, they showed that most of conventional autophagy factors are related with STX17 induced mitophagy in Fis1KO cells. Although there shown large number of experimental results in this paper, each experiments are not well performed to confirm their conclusion. Especially, the observation of mitophagy is not well performed. Most importantly, overexpression of STX17 in Fis1 KO/KD is not physiological situation. This reviewer thinks that the mitophagy induction used in this paper is too artificial and it is not reflects phenomena in vivo.
The authors should consider the following:

The amount of mitochondria change by the balance of mitochondrial synthesis and degradation. Thus, even if mitochondrial protein decreased in STX17 overexpressed Fis1 KO cells, it is not always result of mitophagy. Because it is thought that isolation membrane formed from mitochondria-ER contact site, co-localization of LC3 and mitochondria can be observed even during bulk-autophagy. Accordingly, the authors should observe mitophagy using alternative methods, such as using mito-keima or mito-GFP-mCherry. In addition, it should be tested whether Bafilomycin A1 treatment dramatically increases co-localization of mitochondria and LC3 in STX17 overexpressed Fis1 KO cells.

GFP-STX17 puncta does not co-localize with MTR in Fis1 KO cells (Figure 1f). However, it is shown that GFP-STX17 accumulates on MAM during mitophagy. In addition, Tom20 and STX17 are completely co-localized in Figure 2d. Are the puncta formed STX17 and MAM located STX17 different portion?

Autophagosome should not be labelled by p62 (Page 7 line 3).

In Figure 2d, majority of mitochondria co-localize with Lamp2. This means that almost all mitochondria will soon be degraded in this cell. What is the fate of these cells?

In Figure 3b, GFP-Parkin may co-localize with mitochondria in Fis1 KO and Flag-STX17 overexpressed cell. More cells should be shown to confirm whether GFP-Parkin co-localize with mitochondria or not.

Fig. S2h (p8 line 14) is not shown in Figure S2.

Page 8 line 24 to page 9 line 1; the authors mentioned that both Fis1dTRP2 and Fis1(TRP2+CT) are capable to mediate mitochondrial fission. Based on this result, it is unclear which domain of Fis1 is required for mitochondrial fission.

It is shown that TPR2 of Fis1 interacts with N-terminal domain of STX17. Although TPR2 of Fis1 negatively regulate mitophagy, N-terminal domain of STX17 positively regulate it. This finding makes it difficult to understand the molecular mechanism of mitophagy induction. What is the role of TPR2 of Fis1 and N-terminal domain of STX17 on mitophagy?

The authors demonstrated that STX17 localizes on MAM in Fis1 KO cells. However, it is unclear how STX17 can localize MAM only in Fis1 KO cells.

In Figure 6 d and f, it is shown that punctate formation of GFP-STX17 in Fis1 KO cells is inhibited by KD of ATG5 or ATG14. This mean that isolation membrane formation (or autophagosome) is required for GFP-STX17 puncta formation. Which is the initial step of mitophagy, STX17 puncta formation or isolation membrane formation?

Page 11 line14, (Fig. Sc-e) should be (Fig. S5c-e).

Page 12 line 8, “TBC1D15, the GTPase protein for Rab7” should be “TBC1D15, the GTPase-activating protein for Rab7.”

Reviewer #2 (Remarks to the Author):

In this manuscript, Xian et al. report the role of Syntaxin 17 (STX17), an ER/mitochondria-associated membrane (MAM)-localized SNARE family protein, in mitochondria-specific autophagy (mitophagy). Recent studies establish that STX17 regulates autophagy and mitochondrial fission in mammalian cells, however, whether it also functions in mitophagy has not been explored. In this study, the authors initially focused on Fis1, an evolutionarily conserved mitochondrial outer membrane protein that has been suggested to act in mitochondrial dynamics and mitophagy, and identified STX17 as a Fis1-interacting protein. Strikingly, overexpression of STX17 in cells depleting Fis1 (but not in wild-type cells) caused morphological alteration in mitochondria that were colocalized with LC3 (autophagy marker), p62 (LC3-binding protein), and Lamp2 (lysosome marker), indicating autophagy-dependent mitochondrial degradation. Under the same conditions, the levels of mitochondrial proteins were decreased. Domain mapping analysis revealed that the Fis1 tetratricopeptide repeat 2 (TPR2) and the STX17 N-terminal extension are crucial for Fis1-STX17 interaction, and that the STX17 N-terminal extension is required to promote mitophagy in cells depleting Fis1. In addition, STX17 and ATG14, a subunit of the phosphatidylinositol-3 kinase complex essential for autophagy, localizes to mitochondria and interact with each other in a manner dependent on loss of Fis1. Moreover, STX17 K254C, a variant defective in mitochondrial localization and ATG14 interaction, could not drive mitophagy in Fis1 knockout (KO) cells. The authors also found that mitophagy in cells overexpressing STX17 and depleting Fis1 requires canonical autophagy-related proteins, the small GTPase Rab7, and the transcription factor EB (TFEB). Finally, this type of mitophagy was significantly suppressed in cells under respiration-inducing conditions, suggesting a regulatory link to the mitochondrial metabolic state. Collectively, these findings implicate STX17 acting as a potential inducer of mitophagy and Fis1 acting as the antagonizer through its STX17 binding.

The data in this study are very interesting and could provide new insights into the molecular mechanisms of mitophagy in mammalian cells. However, there is no evidence suggesting that STX17 can promote mitophagy in wild-type cells under physiological conditions (without any genetic manipulations). For example, are there any cell types expressing (and/or culture conditions leading to) low and high levels of Fis1 and STX17, respectively? This manuscript would be significantly strengthened if the authors address this major issue and the following points.

Specific points:

1. In Figure 2e, 7d, S5c, and S5d, the authors should add western blot data for cells treated with lysosomal inhibitors such as Chloroquine or Bafilomycin A1.

2. In Figure 3, the authors should investigate if endogenous Parkin is upregulated in cells depleting Fis1 and overexpressing STX17. If so, Parkin/Fis1 DKO cells should be tested to promote STX17-mediated mitophagy.

3. In Figure 4, the authors should perform co-IP assays to examine STX17 ΔCT-Fis1 and STX17 ΔNT-ATG14 interactions.

4. In Figure 5, the authors should test whether mitochondrial targeting of ATG14 in Fis1 KO cells depends on overexpression of STX17.

5. In Figure 8, the authors should analyze STX17-ATG14 interaction by co-IP and mitochondrial targeting of STX17 and ATG14 by subcellular fractionation for cells grown in galactose medium.

Reviewer #3 (Remarks to the Author):

In the manuscript, Xian et al., showed that Fis1, one of mitochondrial outer membrane fission protein, is able to interact with STX17,and depletion of Fis1 induced mitophagy that is dependent on STX17, but not on Parkin translocation. They further showed that knockout of Fis1 can induce STX translocated on MAM, where it interacts with ATG14 which further recruits core autophagy proteins hierarchically to form mitophagosomes, followed by Rab7-dependent mitophagosome-lysosome fusion. While the results are interesting, much of the work relied on the overexpressing of particular genes and some of the images are not of high quality. The mitophagy assay is mainly based on the colocalization of autophagy gene products on mitochondria. More comprehensive analysis of biochemical hallmarks and by mt-Keima are required. Previous studies has already shown that high level of Fis1 promotes mitophagy, and these literatures need to be discussed in the discussion.
Specifics

1. There are reports showing Parkin independent pathway of mitophagy. For example, PINK1 directly interact with autophagy receptors such as OPTN, NDP52. Also, mitophagy receptors such as NIX, FUNDC1, PHB2 and others have been reported to mediated Parkin independent mitophagy. The authors may be interested to check if these receptors are involved.

2. In Figure 1, the authors showed that FIS1 interacts with STX17 when overexpressed. It is important to check if the endogenous FIS1 interacts with STX17 upon FCCP or Hypoxia stress. It would be better to have quantitative analysis of Figure 1e, 1f. Biochemical analysis of mitochondrial proteins in the outer membrane, inner membrane and mitochondrial matrix in addition to LC3 and p62 is needed. Same to Figure 2 and other figures

4. Figure 2a, it may be better to display key data such as Fis KD conditions and put quantitative analysis of other KO conditions. In Figure 2d, I do not see the expression of GFP signals location for STX17;

5. Figure 2F, EM images are of poor quality. Only GFP-STX17/FIS1 KO samples have autophasomal structures?

6. it would be interesting to check the localization of endogenous STX17 and Rab7 with FIS KO or FCCP stress?

Responses to the Reviewers' Comments

We are deeply thankful for the invaluable perspectives and constructive suggestions from the three reviewers to improve our manuscript. As requested, we have performed the following additional suggested experiments, as described in details below.

Reviewers' comments:

Reviewer #1 (Remarks to the Author):

In this manuscript, the authors identified that Fis1 interacts with STX17. Then, they found that overexpression of STX17 in Fis1 KO/KD cells induce mitophagy. They further studied the specific domain required for Fis1-STX17 interaction and the role of the domains on mitophagy. As previously reported, they showed that STX17 interacts with Atg14 and localizes on MAM. As expected again, they showed that most of conventional autophagy factors are related with STX17 induced mitophagy in Fis1KO cells. Although there shown large number of experimental results in this paper, each experiments are not well performed to confirm their conclusion. Especially, the observation of mitophagy is not well performed. Most importantly, overexpression of STX17 in Fis1 KO/KD is not physiological situation. This reviewer thinks that the mitophagy induction used in this paper is too artificial and it is not reflects phenomena in vivo.

A: We appreciate this reviewer's perspectives and thank the reviewer for pointing out these key issues. Firstly, to elaborate more comprehensive observations of mitophagy, we have extensively strengthened our notion of mitophagy, through conducting mt-Keima assay and examining the turnover of mitochondrial proteins (including OMM, IMS, IMM and matrix proteins), and validated the dramatic rescue effect using the lysosomal inhibitor chloroquine. These new data are now included in Fig 2e, 2g, 2h, S2d, S3d, 6f, 6i and 7d of our revised manuscript.

Secondly, in light of STX17 overexpression, we share the same concern with the reviewer. Nevertheless, to clarify this issue more clearly, here below we also list three lines of evidence to support our study and humbly hope for your consent.

(1) Basically, endogenous expression levels of autophagy/mitophagy-related proteins are generally low. Given this reason, most studies rely on the overexpression of proteins that are involved in autophagy/mitophagy (Elizabeth L. Axe *et al.*, *J Cell Biol*, 2008; Hayashi

Yamamoto *et al.*, *J Cell Biol*, 2012; Maho Hamasaki *et al.*, *Nature*, 2013; Ikuko Koyama-Honda *et al.*, *Autophagy*, 2013; Lei Liu *et al.*, *Nat Cell Biol*, 2012; Tomokazu Murakawa *et al.*, *Nat Commun*, 2015). Notably, the autophagy mediator, STX17, is not an exception. The amount of endogenous STX17 is rather low (Eisuke Itakura *et al.*, *Cell*, 2012), therefore the overexpression system would need to be adopted.

(2) Particularly, to investigate autophagosome/mitophagosome on ER-mitochondria contact sites, well-established techniques including confocal imaging by labelling proteins with fluorescent tags, would need to be carried out, by ectopically overexpressing STX17 (Maho Hamasaki *et al.*, *Nature*, 2013). With the similar motivation, here we unravel a novel role of mitophagic STX17 in a dose-dependent manner, autonomously regulated by Fis1, in which image acquisition needs to be extensively utilized. Therefore, to some extent, overexpression of STX17 is unavoidable.

(3) Additionally, to partially address this concern, during this revision process, we have generated "HeLa cells stably expressing GFP-STX17" and further highlighted the negative role of Fis1 in mitophagy via the STX17-mediated pathway. These results are consolidated in Fig S2 b-d.

The authors should consider the following:

The amount of mitochondria change by the balance of mitochondrial synthesis and degradation. Thus, even if mitochondrial protein decreased in STX17 overexpressed Fis1 KO cells, it is not always result of mitophagy. Because it is thought that isolation membrane formed from mitochondria-ER contact site, co-localization of LC3 and mitochondria can be observed even during bulk-autophagy. Accordingly, the authors should observe mitophagy using alternative methods, such as using mito-keima or mito-GFP-mCherry. In addition, it should be tested whether Bafilomycin A1 treatment dramatically increases co-localization of mitochondria and LC3 in STX17 overexpressed Fis1 KO cells.

A: We thank the reviewer very much for the constructive suggestions to improve our manuscript.

(1) To accomplish the reviewer's first advice in using alternative method to validate mitophagy, we have newly generated WT and Fis1 KO HeLa cells stably expressing mt-Keima. As suggested, we have provided new data to further validate mitophagy,

approached with confocal imaging of mt-Keima marker (Fig 2g). Determined by the ratio of acidified mt-Keima per cell by FACS, as a quantitative indicator of mitophagy, we observed that Fis1 deficiency resulted in a significant higher proportion of mito-lysosome in GFP-STX17 expressing cells from 7.37±2.34% to 56.88±2.24% (Fig 2h and S3d). Our data clearly demonstrate that STX17 initiates mitophagy upon Fis1 loss.

(2) As requested by the reviewer, we have also examined the co-localization between mitochondria (Tim23) and autophagosome (LC3) in STX17-mediated mitophagy (Fig R1 as attached below). Notably, Bafilomycin A1 is well-known to inhibit the fusion of autophagosome-lysosome and block the lysosomal degradation (Donelly A. van Schalkwyk *et al*., *Biochemical Pharmacology*, 2010). As expected, Bafilomycin A1 treatment markedly increased LC3 accumulation in both WT and Fis1 KO cells, with or without the overexpression of STX17 (comparing transfected cells with non-transfected cells), probably due to the inhibition of bulk autophagy (Fig R1a as attached below). However, the colocalization between mitochondria and autophagosome upon Bafilomycin A1 treatment was drastically decreased (Fig R1b as attached below, shown as aberrant accumulation of macroautophagosome). In line with this, we additionally demonstrated that mitophagy is blocked by another lysosome inhibitor, chloroquine (CQ) (please see Fig 2e and Fig S2d), suggesting that lysosomal inhibitors inhibit STX17-mediated mitophagy, but preferentially prime cells to the accumulation of bulk autophagosome. Given these results, we would interpret that, as selective autophagy, mitophagy may be distinct from non-selective macroautophagy. Adapting to acute stress, bulk autophagy may respond as the first-aid.

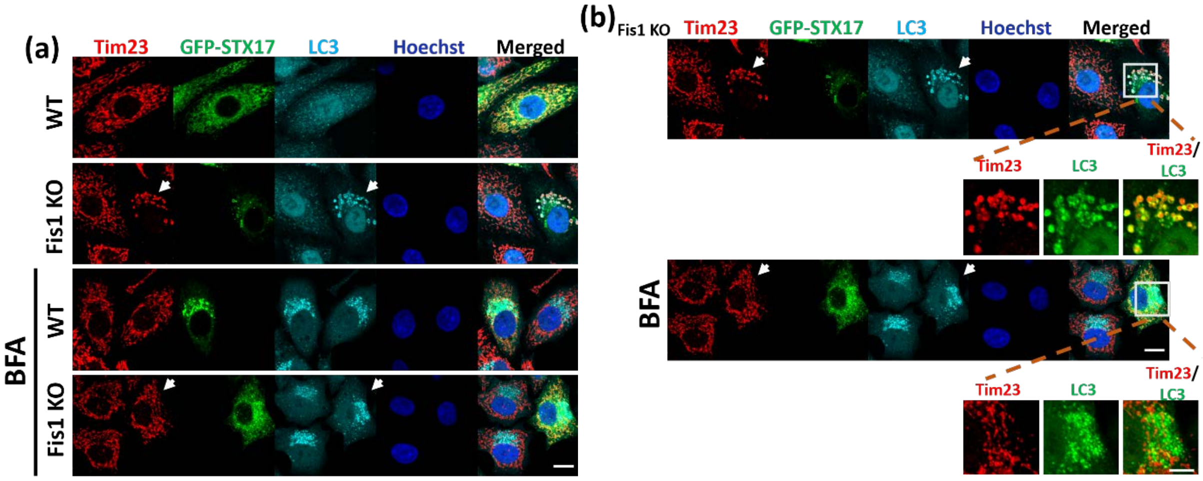

Figure R1 (a) WT or Fis1 KO HeLa cells were transfected with plasmid encoding GFP-STX17 (green) for 6 h and cells were cultured in medium with or without Bafilomycin A1 (BFA) at 100 nM for further 18 h. Cells were fixed and immunostained with Tim23 (red) and LC3 (cyan) antibodies. Hoechst, blue. White arrows indicate Fis1 KO cells expressing GFP-STX17. Scale bar, 10 µm. (b) The colocalization of Tim23 (red) and LC3 (green) in Fis1 KO cells with or without Bafilomycin A1 treatment from (a). Scale bar, 5 µm.

GFP-STX17 puncta does not co-localize with MTR in Fis1 KO cells (Figure 1f). However, it is shown that GFP-STX17 accumulates on MAM during mitophagy. In addition, Tom20 and STX17 are completely co-localized in Figure 2d. Are the puncta formed STX17 and MAM located STX17 different portion?

A: We appreciate the reviewer for pointing out this question. Firstly, we are sorry that we may not state or interpret Fig 2d clearly. In Fig 2d, we show the organization of Tom20 (mitochondria) and STX17 by zooming in a part of original image in Fis1 KO panel, whereby in this cell, Tom20 and STX17 are not completely co-localized but partially overlapped. To further validate this data, we additionally carried out immunofluorescence assay by labelling mitochondria with MTR and Tom20 simultaneously (Fig S1g). In Fis1 KO cells, STX17, as expected, shows punctate pattern. Importantly, the colocalization between GFP-STX17 and mitochondria in this cell is partial. As indicated in Fig S1g, in cropped image 1 (white arrows), STX17 completely colocalized with Tom20 and MTR, probably representative of mitophagosome. However, visualized by enlarged image 2, GFP-STX17 partially colocalized with mitochondria (purple arrow indicates non-colocalization), suggesting GFP-STX17 co-localizes with mitochondria but not essentially completely overlaps with mitochondria. Additionally, especially shown by 2, MTR unlikely colocalizes with Tom20 perfectly, because MTR signal relies on mitochondrial membrane potential but Tom20 does not. It is understandable that MTR may not show exactly the same pattern as Tom20.

Autophagosome should not be labelled by p62 (Page 7 line 3).

A: We are very sorry for this error and thank the reviewer very much for pointing out. We have amended this statement as "fragmented mitochondria were enclosed in

autophagosomes (LC3), autophagy receptor (P62), and lysosomes (Lamp2) observed by three-dimensional reconstructed images."

In Figure 2d, majority of mitochondria co-localize with Lamp2. This means that almost all mitochondria will soon be degraded in this cell. What is the fate of these cells?

A: We are grateful to this reviewer for this interesting question. Mitochondria are considered as energy powerhouse of cells and mitochondrial removal may result in cellular homeostatic dysregulation. Therefore, it is reasonable for us to couple mitophagy with cell survival. To address this, we employed propidium iodide (PI) to analyse cell death. PI is a fluorescent dye which binds to DNA, but PI could not passively traverse into healthy cells with intact plasma membrane. To this end, PI uptake determines dead cells in which plasma membranes become permeable. Interestingly, shown by Fig R2 below, we observed significantly increased population of dead cells with permeable plasma membrane during STX17-mediated mitophagy. Strikingly, PI intensity of cells was enhanced by 0.39±0.05 fold, substantiating that STX17-medaited mitophagy leads to cell death.

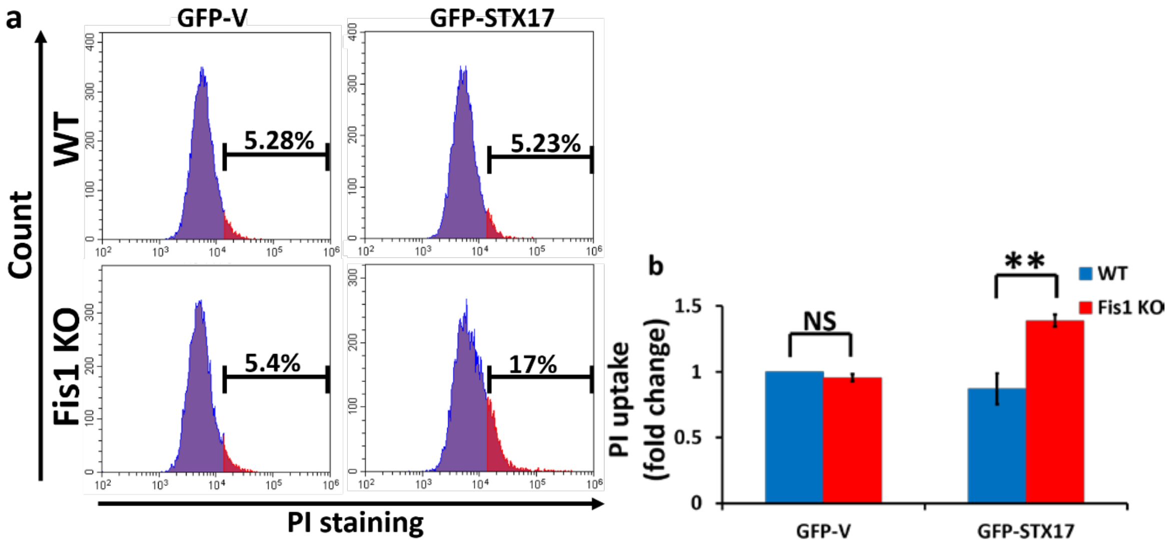

Figure R2 (a) WT or Fis1 KO HeLa cells were transiently transfected with GFP-tagged vector or STX17 for 72 h. Then cells were cultured in DMEM containing propidium iodide (PI) for further 15 min. The PI uptake of cells was analysed by flow cytometry. The percentage of cells with high PI uptake indicates the population of dead cells with permeable plasma membrane. (b) Quantification of the PI intensity from three independent experiments as in (a). Bars indicate mean ± SD. **P<0.01, NS, not significant.

In Figure 3b, GFP-Parkin may co-localize with mitochondria in Fis1 KO and Flag-STX17 overexpressed cell. More cells should be shown to confirm whether GFP-Parkin co-localize with mitochondria or not.

A: We are thankful for this constructive suggestion from this reviewer. To address this, we have examined the localization of Parkin during STX17-induced mitophagy upon Fis1 loss (Fig 3a, last panel). Parkin was not observed to co-localize with mitochondria even in Fis1 KO and Flag-STX17 overexpressed cells, despite striking mitochondria clearance was appreciated. In addition, our quantitative analysis using more than 150 cells further validates that STX17-mediated mitophagy upon Fis1 loss is not related to Parkin recruitment (Fig 3b).

Fig. S2h (p8 line 14) is not shown in Figure S2.

A: We are sorry that we may not elaborate the original Fig S2h clearly in Fig S2. Given new data applied to our revised manuscript and the re-organization of figures, we have re-arranged Fig S2h from the original manuscript to new Fig S4f in current revised manuscript, demonstrating that no gross enrichment of mitochondrial Ub was apparent in STX17-induced mitophagy of Fis1 KO cells.

Page 8 line 24 to page 9 line 1; the authors mentioned that both Fis1dTRP2 and Fis1(TRP2+CT) are capable to mediate mitochondrial fission. Based on this result, it is unclear which domain of Fis1 is required for mitochondrial fission.

A: We appreciate this reviewer for this question. We apologize for the original data without quantification. Here we have carried out comprehensive quantitative analyses to verify mitochondrial fragmentation using these truncations of Fis1. As shown in Fig S5a, we observed reduced mitochondrial fission from 72.3±4.2% in Fis1 FL-overexpressing cells to 24.8±2.3% in Fis1ΔTPR2-expressing cells or 56.1±1.9% of Fis1 (TPR2+CT)-expressing cells, suggesting that both TPR2 and additional domain beside TPR2 domain account for mitochondrial fission, essentially supporting a previous study (Tianzheng Yu *et al.*, J Cell Sci, 2005).

It is shown that TPR2 of Fis1 interacts with N-terminal domain of STX17. Although TPR2 of Fis1 negatively regulate mitophagy, N-terminal domain of STX17 positively regulate it. This finding makes it difficult to understand the molecular mechanism of mitophagy induction. What is the role of TPR2 of Fis1 and N-terminal domain of STX17 on mitophagy?

A: We thank this reviewer for pointing out this key notion. Yes, our results show that (1) The TPR2 domain is essential for Fis1 to bind with the N terminus of STX17. (2) The TPR2 domain of Fis1 negatively regulates mitophagy. (3) The N-terminus of STX17 positively regulates mitophagy. Given these data, we elaborate that through interacting with the "active" domain (the N-terminal domain) of STX17, the TPR2 domain of Fis1 prevents the over-translocation and aberrant accumulation of STX17 onto MAM and mitochondria. Therefore, Fis1 negatively regulates the formation of mitophagosome (Fig 9). Collectively, we reveal that Fis1 governs the onset of mitophagy, by "gatekeeping" the accessive recruitment of STX17 onto MAM/mitochondria.

The authors demonstrated that STX17 localizes on MAM in Fis1 KO cells. However, it is unclear how STX17 can localize MAM only in Fis1 KO cells.

A: To address this concern, we repeated our previous data by Percoll density-gradient centrifuge using WT and Fis1 KO cells. In attempt to visualize STX17 in mitochondrial fraction, we increased the loading amount of mitochondrial fraction equally for WT and Fis1 KO cells and performed immunoblotting. As indicated in Fig S6, compared with WT, increased translocation of endogenous STX17 onto MAM and mitochondrial fractions was apparent upon Fis1 loss, suggesting that Fis1 autonomously regulates the dynamic trafficking of STX17 among ER, MAM and mitochondria, probably mediated through the specific interaction between Fis1 and STX17. We humbly hope this could answer your doubt.

In Figure 6 d and f, it is shown that punctate formation of GFP-STX17 in Fis1 KO cells is inhibited by KD of ATG5 or ATG14. This mean that isolation membrane formation (or autophagosome) is required for GFP-STX17 puncta formation. Which is the initial step of mitophagy, STX17 puncta formation or isolation membrane formation?

A: Thank the reviewer for this interesting question. Yes, we have further confirmed that the puncta formation of GFP-STX17 in Fis1 KO cells is indeed inhibited by the depletion

of ATG5 or ATG14 (Fig S7c-e), suggesting that isolation membrane proteins ATG5 and ATG14 modulate the puncta formation of GFP-STX17. To further address the sequential step between STX17 puncta formation and isolation membrane formation, we devoted efforts in live cell imaging, to determine the initiation of mitophagy in Fis1 KO cells (Fig R3 as attached below). Of note, as early as 6 h of post-transfection by GFP-ATG14 (to label isolation membrane) and mCherry-STX17 (to indicate STX17 puncta), STX17 puncta was observed to form and isolation membrane was initiated by Fis1 loss. In addition, given the close relationship between ATG14 and STX17, in the cases whereby STX17 was observed to form puncta in Fis1 KO cells, ATG14 aggregated as puncta, co-localized with STX17 perfectly. These lines of evidence essentially reached the notion that, to discriminate the sequence between STX17 puncta and isolation membrane initiation would be difficult, and complementarily, STX17 itself could be a crucial part for isolation membrane as well. Taken together, our data reached the conclusion that isolation membrane is crucial for STX17 puncta formation and vice versa.

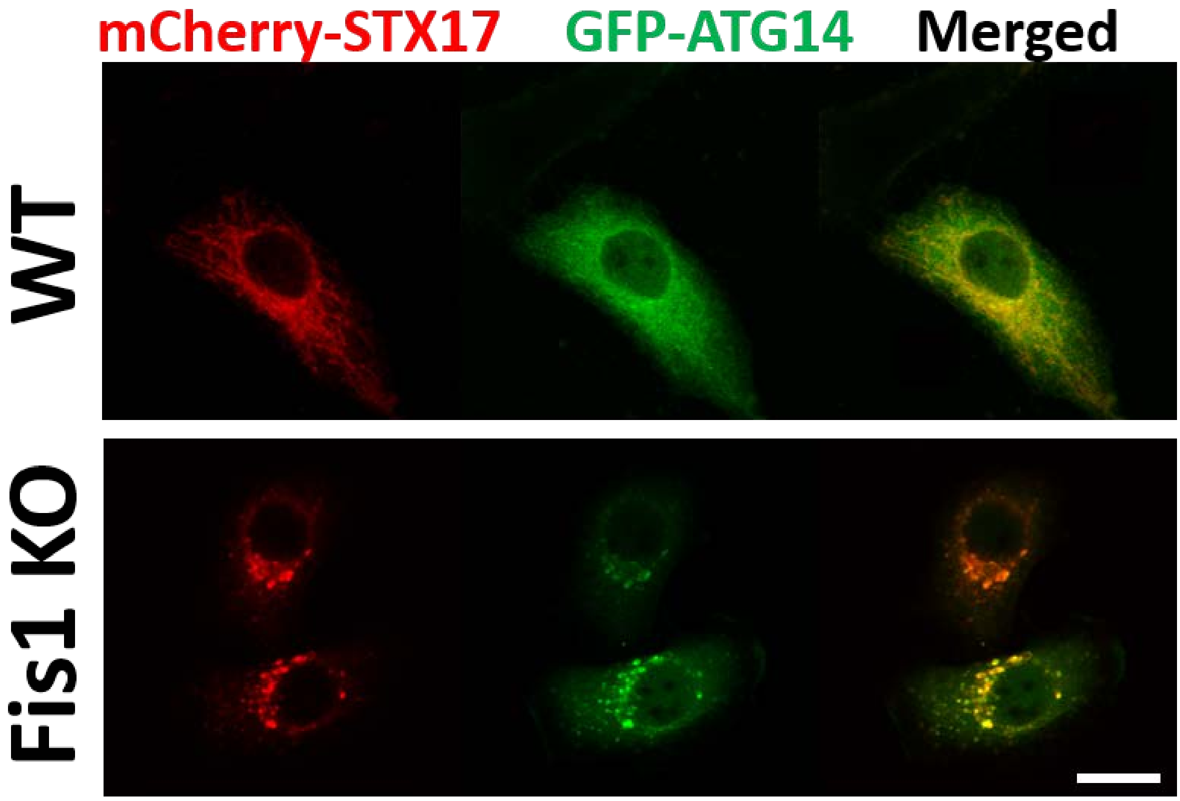

Figure R3 WT or Fis1 KO HeLa cells were transfected with plasmid encoding mCherry-STX17 (red) and GFP-ATG14 (green) for 6 h. Live cells were imaged. Scale bar, 10 µm.

Page 11 line14, (Fig. Sc-e) should be (Fig. S5c-e).

A: We are very sorry for this error and thank the reviewer very much for reminding. Given more data included in our revised manuscript, we have re-organized the figures and it has been amended accordingly.

Page 12 line 8, "TBC1D15, the GTPase protein for Rab7" should be "TBC1D15, the GTPase-activating protein for Rab7."

A: We apologize for this mistake and appreciate the reviewer for kindly pointing out. Yes, we have amended the phrase as "TBC1D15, the GTPase-activating protein for Rab7" (page 13).

Reviewer #2 (Remarks to the Author):

In this manuscript, Xian et al. report the role of Syntaxin 17 (STX17), an ER/mitochondria-associated membrane (MAM)-localized SNARE family protein, in mitochondria-specific autophagy (mitophagy). Recent studies establish that STX17 regulates autophagy and mitochondrial fission in mammalian cells, however, whether it also functions in mitophagy has not been explored. In this study, the authors initially focused on Fis1, an evolutionarily conserved mitochondrial outer membrane protein that has been suggested to act in mitochondrial dynamics and mitophagy, and identified STX17 as a Fis1-interacting protein. Strikingly, overexpression of STX17 in cells depleting Fis1 (but not in wild type cells) caused morphological alteration in mitochondria that were colocalized with LC3 (autophagy marker), p62 (LC3-binding protein), and Lamp2 (lysosome marker), indicating autophagy dependent mitochondrial degradation. Under the same conditions, the levels of mitochondrial proteins were decreased.

Domain mapping analysis revealed that the Fis1 tetratricopeptide repeat 2 (TPR2) and the STX17 N-terminal extension are crucial for Fis1-STX17 interaction, and that the STX17 N-terminal extension is required to promote mitophagy in cells depleting Fis1. In addition, STX17 and ATG14, a subunit of the phosphatidylinositol-3 kinase complex essential for autophagy, localizes to mitochondria and interact with each other in a manner dependent on loss of Fis1. Moreover, STX17 K254C, a variant defective in mitochondrial localization and ATG14 interaction, could not drive mitophagy in Fis1 knockout (KO) cells. The authors

also found that mitophagy in cells overexpressing STX17 and depleting Fis1 requires canonical autophagy-related proteins, the small GTPase Rab7, and the transcription factor EB (TFEB). Finally, this type of mitophagy was significantly suppressed in cells under respiration-inducing conditions, suggesting a regulatory link to the mitochondrial metabolic state. Collectively, these findings implicate STX17 acting as a potential inducer of mitophagy and Fis1 acting as the antagonizer through its STX17 binding.

The data in this study are very interesting and could provide new insights into the molecular mechanisms of mitophagy in mammalian cells. However, there is no evidence suggesting that STX17 can promote mitophagy in wild-type cells under physiological conditions (without any genetic manipulations). For example, are there any cell types expressing (and/or culture conditions leading to) low and high levels of Fis1 and STX17, respectively? This manuscript would be significantly strengthened if the authors address this major issue and the following points.

A: We thank Reviewer #2 very much for highlighting that our current study "*provides interesting data to the new sights into the molecular mechanisms of mitophagy in mammalian cells*". Nevertheless, we share the same concern with this reviewer on the physiological implications of this study. To address this key question, we have devoted many efforts and examined the level of STX17 and Fis1 respectively, by applying plethora of mitochondrial toxins (CCCP, FCCP, valinomycin, oligomycin, antimycin, H2O2, and hypoxia) and ER stresses (glucose deprivation, thapsigargin, tunicamycin, dithiothreitol, EGTA). Unfortunately, at this stage, we fail to observe significant change on the levels of Fis1 and STX17 simultaneously or satisfactory induction of mitophagy in those conditions. Perhaps more future work to follow up our initiation by this current study would be needed.

Here below we also list three lines of evidence to support our study and humbly hope for your consent.

(1) Basically, endogenous expression levels of autophagy/mitophagy-related proteins are generally low. Given this reason, most studies rely on the overexpression of proteins that are involved in autophagy/mitophagy (Elizabeth L. Axe *et al., J Cell Biol*, 2008; Hayashi Yamamoto *et al., J Cell Biol*, 2012; Maho Hamasaki *et al., Nature*, 2013; Ikuko Koyama-Honda *et al., Autophagy*, 2013; Lei Liu *et al., Nat Cell Biol*, 2012; Tomokazu Murakawa

***et al.*, *Nat Commun*, 2015). Notably, the autophagy mediator, STX17, is not an exception. The amount of endogenous STX17 is rather low (Eisuke Itakura *et al.*, *Cell*, 2012), therefore the overexpression system would need to be adopted.**

(2) Particularly, to investigate autophagosome/mitophagosome on ER-mitochondria contact sites, well-established techniques including confocal imaging by labelling proteins with fluorescent tags, would need to be carried out, by ectopically overexpressing STX17 (Maho Hamasaki *et al.*, *Nature*, 2013). With the similar motivation, here we unravel a novel role of mitophagic STX17 in a dose-dependent manner, autonomously regulated by Fis1, in which image acquisition needs to be extensively utilized. Therefore, to some extent, overexpression of STX17 is unavoidable.

(3) Additionally, to partially address this concern, during this revision process, we have generated "HeLa cells stably expressing GFP-STX17" and further highlighted the negative role of Fis1 in mitophagy via the STX17-mediated pathway. These results are consolidated in Fig S2 b-d.

We are deeply grateful to this reviewer for pointing out several detailed suggestions and comments to improve our manuscript.

Specific points:

1. In Figure 2e, 7d, S5c, and S5d, the authors should add western blot data for cells treated with lysosomal inhibitors such as Chloroquine or Bafilomycin A1.

A: We appreciate this valuable advice from this reviewer. In this revised manuscript, we have applied chloroquine (CQ) to the immunoblotting analyses to detect the turnover of the overall mitochondrial proteins. Given more data included, we have re-arranged the original figures. These new data are shown in Fig 2e, S2d, 6f, 6i, 7d. As expected, the treatment of lysosomal inhibitor significantly blocks STX17-mediated mitochondrial turnover upon Fis1 loss, substantiating that mitophagy allows for the overall reduction of mitochondrial protein levels.

2. In Figure 3, the authors should investigate if endogenous Parkin is upregulated in cells depleting Fis1 and overexpressing STX17. If so, Parkin/Fis1 DKO cells should be tested to promote STX17-mediated mitophagy.

A: We thank this reviewer very much for the thoughtful suggestion. To address this, we have newly carried out immunoblotting analysis in STX17-expressing HeLa cells upon Fis1 loss, to examine the expression level of endogenous Parkin (Fig 3e). Conceivably, Parkin was not detected in GFP-STX17 expressing cells upon Fis1 loss, albeit significant degradation of mitochondrial proteins was observed, coupled with mitophagy, illustrating that Parkin is not involved in STX17-induced mitophagy upon Fis1 depletion.

3. In Figure 4, the authors should perform co-IP assays to examine STX17 ΔCT-Fis1 and STX17 ΔNT-ATG14 interactions.

A: We deeply appreciate the valuable suggestion from this reviewer. Regarding the transmembrane domain (CT) of STX17, we examined the interaction of STX17ΔCT and Fis1 by pull-down assay (Fig 4f, lower panel). In line with our result indicated in Fig 4g that STX17ΔCT failed to induce mitophagy, the cytosolic-localized STX17ΔCT, is unable to interact with mitochondrial Fis1. In addition, we have also performed additional Co-IP experiment using Flag-STX17ΔNT and Myc-ATG14 (Fig S6b). The drastic decrease by 0.66±0.19 fold of the interaction between STX17ΔNT and ATG14 is consistent with the indispensability of N-terminus of STX17 to induce mitophagy (Fig 4g-h & Fig S5 c-e).

4. In Figure 5, the authors should test whether mitochondrial targeting of ATG14 in Fis1 KO cells depends on overexpression of STX17.

A: We are very thankful to this reviewer's constructive advice. To achieve this, we investigated the subcellular localization of ATG14 through the Percoll density-gradient centrifuge using WT and Fis1 KO cells, without the overexpression of STX17 (Fig S6a). Notably, without the introduction of STX17, ATG14 remained on the fraction of ER in Fis1 KO cells, despite of the apparent translocation of the endogenous STX17 onto MAM and mitochondria, supporting that during mitophagy, STX17 accounts for the

recruitment of isolation membrane protein ATG14 onto MAM and mitochondria, in a dose-dependent manner.

5. In Figure 8, the authors should analyze STX17-ATG14 interaction by co-IP and mitochondrial targeting of STX17 and ATG14 by subcellular fractionation for cells grown in galactose medium.

A: We are very grateful to the reviewer for these insightful suggestions and would like to characterize this. To address this issue, cells were cultured in galactose medium and subsequent Co-IP was performed to analyze the interaction of STX17-ATG14. Not surprisingly, the association between STX17 and ATG14 cultured in galactose was reduced by 0.55±0.21 fold, compared with cells cultured in glucose (Fig 8e). In addition, robust decreases of GFP-STX17 and ATG14 in MAM/mitochondrial fractions were detected upon galactose culturing (Fig 8f-h). Taken together, these results both clearly reached complementation to our conclusion that galactose suppresses STX17-medaited mitophagy (Fig 8c-d), probably by interfering the interaction of ATG14 and STX17 and the recruitment of isolation membrane proteins onto MAM/mitochondria.

Reviewer #3 (Remarks to the Author):

In the manuscript, Xian et al., showed that Fis1, one of mitochondrial outer membrane fission protein, is able to interact with STX17, and depletion of Fis1 induced mitophagy that is dependent on STX17, but not on Parkin translocation. They further showed that knockout of Fis1 can induce STX translocated on MAM, where it interacts with ATG14 which further recruits core autophagy proteins hierarchically to form mitophagosomes, followed by Rab7-dependent mitophagosome-lysosome fusion. While the results are interesting, much of the work relied on the overexpressing of particular genes and some of the images are not of high quality. The mitophagy assay is mainly based on the colocalization of autophagy gene products on mitochondria. More comprehensive analysis of biochemical hallmarks and by mt-Keima are required. Previous studies has already shown that high level of Fis1 promotes mitophagy, and these literatures need to be discussed in the discussion.

A: We appreciate this reviewer very much for pointing out that this is an interesting study.

Here below is to address the concerns raised by this reviewer.

(a) Regarding the overexpression of proteins, we reasoned that:

(1) Basically, endogenous expression levels of autophagy/mitophagy-related proteins are generally low. Given this reason, most studies rely on the overexpression of proteins that are involved in autophagy/mitophagy (Elizabeth L. Axe *et al.*, *J Cell Biol*, 2008; Hayashi Yamamoto *et al.*, *J Cell Biol*, 2012; Maho Hamasaki *et al.*, *Nature*, 2013; Ikuko Koyama-Honda *et al.*, *Autophagy*, 2013; Lei Liu *et al.*, *Nat Cell Biol*, 2012; Tomokazu Murakawa *et al.*, *Nat Commun*, 2015). Notably, the autophagy mediator, STX17, is not an exception. The amount of endogenous STX17 is rather low (Eisuke Itakura *et al.*, *Cell*, 2012), therefore the overexpression system would need to be adopted.
(2) Particularly, to investigate autophagosome/mitophagosome on ER-mitochondria contact sites, well-established techniques including confocal imaging by labelling proteins with fluorescent tags, would need to be carried out, by ectopically overexpressing STX17 (Maho Hamasaki *et al.*, *Nature*, 2013). With the similar motivation, here we unravel a novel role of mitophagic STX17 in a dose-dependent manner, autonomously regulated by Fis1, in which image acquisition needs to be extensively utilized. Therefore, to some extent, overexpression of STX17 is unavoidable.
(3) Additionally, to partially address this concern, during this revision process, we have generated "HeLa cells stably expressing GFP-STX17" and further highlighted the negative role of Fis1 in mitophagy via the STX17-mediated pathway. These results are consolidated in Fig S2 b-d.

(b) To elaborate more comprehensive observation of mitophagy, we have strengthened our notion of mitophagy, through conducting mt-Keima assay and using biochemical hallmarks to confirm the turnover of mitochondrial proteins (including OMM, IMS, IMM and matrix proteins), further validated by the dramatic rescue effect by the lysosomal inhibitor CQ. These new data are included in Fig 2e, 2g, 2h, S2d, S3d, 6f, 6i and 7d of our revised manuscript.

(c) For the reviewer's concern on Fis1, we have supplemented the discussion of Fis1 functions in PINK1/Parkin-dependent mitophagy and FUNDC1-mediated aggrephagy under proteostatic stress (page 17-18), which probably are distinct from STX17-mediated mitophagy we are unravelling in this study.

We deeply appreciate this reviewer for pointing out several invaluable suggestions and comprehensive comments to improve our manuscript.

Specifics

1. There are reports showing Parkin independent pathway of mitophagy. For example, PINK1 directly interact with autophagy receptors such as OPTN, NDP52. Also, mitophagy receptors such as NIX, FUNDC1, PHB2 and others have been reported to mediated Parkin independent mitophagy. The authors may be interested to check if these receptors are involved.

A: We are grateful for the kind advice from the reviewer. To address this question, we substantially analyzed mitophagy by RNA interference of mitophagy receptors, including OPTN, NDP52, NIX, FUNDC1, PHB2 (Fig S9). We did not observe any significant impact of these canonical mitophagy receptors on STX17-indcued mitophagy upon Fis1 loss, when we depleted these typical mitophagy receptors. These results illustrate that STX17-mediated mitophagy is more likely via a macroautophagic route, but independent of canonical mitophagy receptors.

2. In Figure 1, the authors showed that FIS1 interacts with STX17 when overexpressed. It is important to check if the endogenous FIS1 interacts with STX17 upon FCCP or Hypoxia stress. It would be better to have quantitative analysis of Figure 1e, 1f. Biochemical analysis of mitochondrial proteins in the outer membrane, inner membrane and mitochondrial matrix in addition to LC3 and p62 is needed. Same to Figure 2 and other figures.

A: We thank this reviewer very much for these constructive suggestions to improve our work.

Firstly, to address the endogenous interaction of Fis1 and STX17 upon FCCP or hypoxia stress, we have devoted many efforts and tried very hard to optimize conditions for the pull-down assay. Many attempts have been made to apply for this Co-IP assay. As shown in Fig R4a (as attached below), we found no appreciable effect of FCCP or hypoxia on the endogenous interaction between STX17 and Fis1 (indicated by red arrowhead, lower

band than the non-specific band shown by cyan asterisk). Unfortunately, we need to point out that the solely available commercial STX17 antibody is produced in rabbit, whereas the rabbit-Fis1 antibody is more efficient for Co-IP, compared with the mouse-Fis1 antibody (Fig R4b as attached below). In this case, after Co-IP, the immunoblotting analysis of STX17 (Fig R4a, around 37 kDa) would easily cross-talk with the heavy chain band (around 55kDa) and the light chain band (around 25kDa) of rabbit Fis1, all recognized by the anti-rabbit secondary antibody. On the other hand, endogenous STX17 level is rather low (which could not be recognized by the STX17 antibody from immunofluorescent analysis, shown in Fig R5 below), supporting that the endogenous interaction between Fis1 and STX17 might be very difficult to detect.

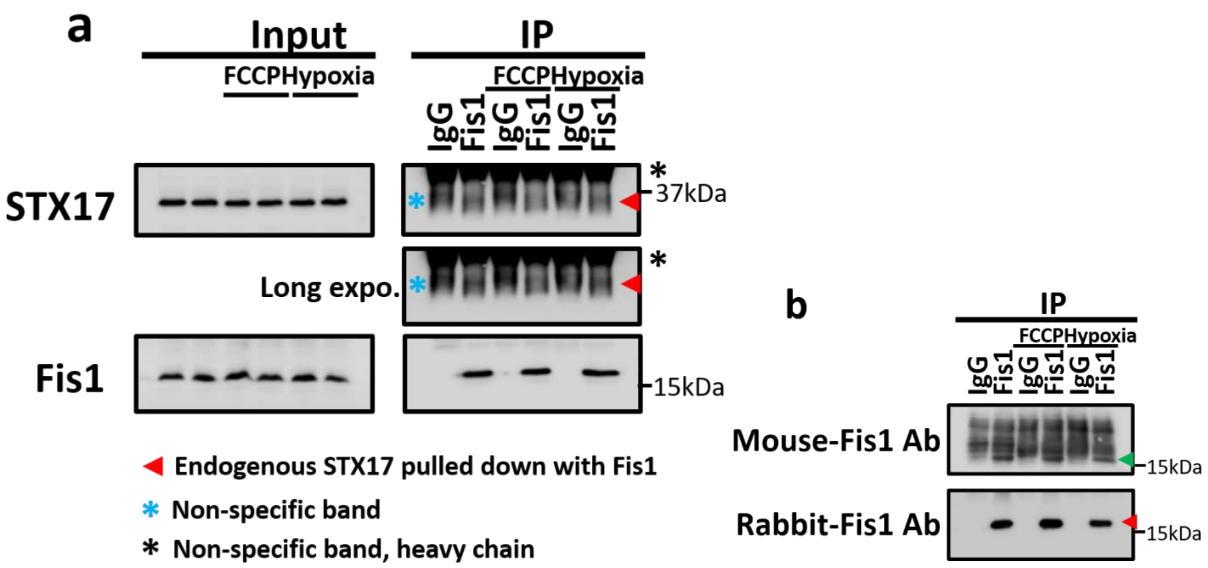

Figure R4 (a) HeLa cells were treated with or without FCCP at 10 μM for 6 h, or Cobalt (II) Chloride hexahydrate at 150 μM for 48 to stimulate hypoxia stress. Cells were solubilized for IP with anti-IgG or anti-Fis1 (rabbit), and analyzed with STX17 and Fis1 (rabbit) antibodies respectively. (b) Cells treated as (a) were harvested. Endogenous Fis1 was recruited by immunoprecipitation using mouse Fis1 antibody or rabbit Fis1 antibody, and further analysed by immunoblotting using rabbit Fis1 antibody.

For the concern of quantitative analyses of Fig 1e, we are sorry that here we need to explain that, image in the last panel (cells co-transfected with GFP-STX17 and mCherry-Fis1) is to show the partial localization of STX17 and Fis1, complementary by the line scan analysis (Fig S1c), but not to indicate the percentage of cells successfully co-

transfected with STX17 and Fis1. As for Fig 1f, as suggested, we have newly included the quantification of cells with decreased MTR to Fig 1g. In Fis1-deficient cells, 45.5±2.0% cells possessed GFP-tagged STX17 punctate structures and markedly abrogated MitoTracker signal.

In light of the biochemical analysis of the turnover of overall mitochondrial proteins, firstly, unfortunately we failed to get substantial reduction of mitochondrial proteins by transfecting cells with Fis1 siRNA (in duration of 72 h) and transient overexpression of GFP-STX17 (in duration of 48 h). One possibility to explain this scenario could be the low efficiency of transient transfection of GFP-STX17 into Fis1-silenced cells (rather than Fis1 KO cells). To address this issue, we have devoted many efforts and newly generated HeLa cells stably expressing GFP-STX17 (Fig S2b-c). We depleted Fis1 by RNA interference in HeLa cells stably expressing GFP-STX17, and further validated this conclusion by treating cells with lysosomal inhibitor chloroquine (CQ) (Fig S2d). We humbly hope these new data may clarify your doubts.

4. Figure 2a, it may be better to display key data such as Fis KD conditions and put quantitative analysis of other KO conditions. In Figure 2d, I do not see the expression of GFP signals location for STX17.

A: We are grateful for this reviewer's valuable advice. The quantitative analysis of Fig 2a has been included in Fig 2b of our revised manuscript. The population of cells with decreased mitochondria mediated by STX17 was 39.1±1.2% in Fis1 siRNA-treated cells, comparing to less than 2% in other control proteins siRNA-treated cells.

For the concern of Fig 2d, we are sorry that we may not state or interpret our result clearly in the original manuscript. GFP-STX17 formed puncta in Fis1 KO cells, which is indicated by white arrow in this revised manuscript.

5. Figure 2F, EM images are of poor quality. Only GFP-STX17/FIS1 KO samples have autophasomal structures?

A: We appreciate this reviewer very much for pointing out this concern. We have applied new set of EM images to Fig 2f, with higher quality, especially emphasized in obvious formation of mitophagosomes, labelled by yellow arrows. In control cells, autophagosomal structures indeed exist but mitophagosome occurs very few as less than 10% (Fig S3b). However, as we are focusing on mitochondria, we apologize that we did not include macroautophagosome structures here.

6. it would be interesting to check the localization of endogenous STX17 and Rab7 with FIS KO or FCCP stress?

A: We deeply appreciate these kind advices from this reviewer.

Regarding the endogenous STX17, we apologize that we failed to find a suitable commercial antibody for immunostaining analysis (Fig R5 appended as below). As shown in Fig R5a, the STX17 antibody (Sigma HPA001204) is applicable for overexpressed STX17 whereas it cannot detect the endogenous STX17. For the STX17 antibody (Invitrogen PA5-40127), endogenously, it picked up significant nonspecific signal (Fig R5b). This nonspecific background signal is validated by STX17 antibody (Invitrogen PA5-40127) pre-coated with Flga-STX17 antigen (second panel in Fig R5b as below). Even though the signal of STX17 antibody was reduced significantly, by the pre-incubation with the antigen of Flag-STX17 protein (seen from the drastic reduced ratio of STX17 signal to Flag signal in Flag-STX17-expressing cell), the nonspecific background still remained, suggesting that the signal is not specifically indicating endogenous STX17. Taken together, there is no appreciable result to detect endogenous STX17 by commercial antibodies. On the other hand, these data further substantiate that the basal expression level of STX17 is rather low. However, mitophagy may require the regulation of STX17 in a dose-dependent manner.

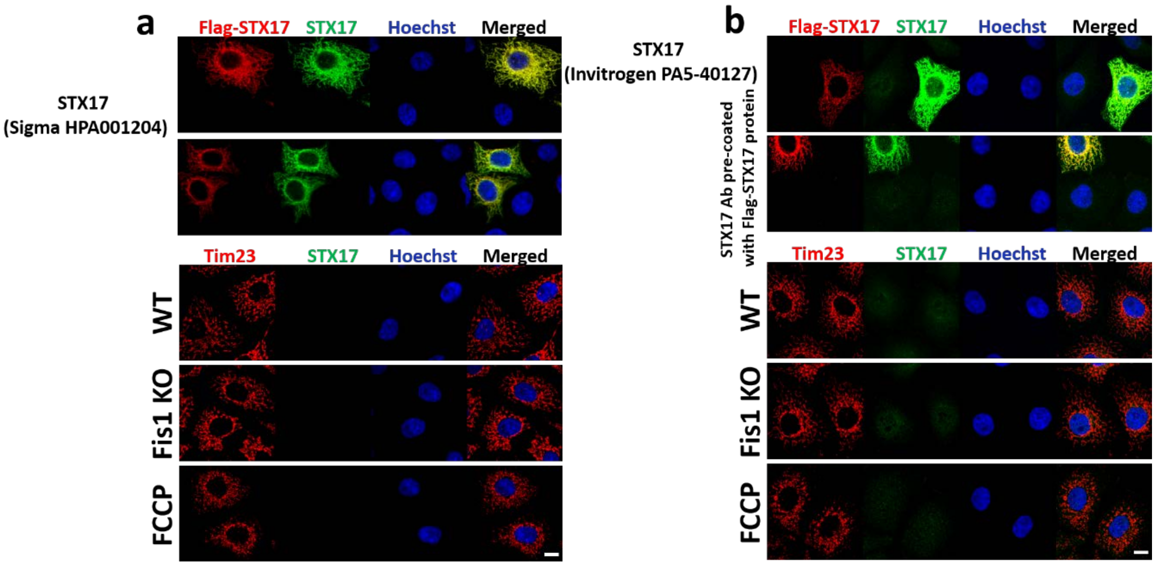

Figure R5 The immunostaining of two STX17 antibodies respectively. (a) Cells transfected with Flag-tagged STX17 (the upper two panels), WT, Fis1 or WT HeLa cells treated with 10 μM FCCP for 6 h (the lower three panels) were fixed and immunostained against STX17 (Sigma HPA001204) (green) and Flag (the upper two panel) or Tim23 (the lower three panels) (red). Hoechst, blue. Scale bar, 10 μm. (b) Cells treated as in (a) were stained using STX17 antibody (Invitrogen PA5-40127) with or without incubation of Flag-STX17 protein. Hoechst, blue. Scale bar, 10 μm.

In light of Rab7, as suggested, we have also further examined the localization of endogenous Rab7. As indicated in Fig S8a, no appreciable difference of the perinuclear localization of Rab7 was observed among WT, Fisi1 KO and FCCP treatment.

REVIEWERS' COMMENTS:

Reviewer #1 (Remarks to the Author):

The authors have made a substantial effort and included new data to address the comments made by this reviewer. I believe that the current manuscript becomes suitable for publication.

Reviewer #2 (Remarks to the Author):

In this manuscript, the authors provided additional data and descriptions to clarify most of the issues raised by the referees. At present, the revised manuscript has significantly been improved to be warranted for publication in Nature Communications with one definition change.

Definition point

1. Whether mitophagy is promoted in cells under some specific physiological conditions without both disrupting Fis1 and overexpressing GFP/mCherry/BFP/Myc/Flag-tagged STX17 still remains to be uncertain. The authors should describe “STX17-mediated mitophagy” more carefully like “STX17 overexpression-mediated mitophagy”.

Reviewer #3 (Remarks to the Author):

the authors have addressed my concerns. It has been recently reported that SNX17 involves FUNDC1 and Parkin (EMBO J. 2018 Nov 2). The author may discuss this in the discussion.

Responses to the Reviewers' Comments

We are again deeply thankful for the through comments and comprehensive perspectives from the three reviewers. As requested, to improve our manuscript, we have complemented the regulation of STX17 on FUNDC1 and PGAM5 in PINK1/Parkin-dependent mitophagy into Discussion section. Additionally, we have amended our description of "STX17-mediated mitophagy" accordingly, as described in details below.

REVIEWERS' COMMENTS:

Reviewer #1 (Remarks to the Author):

The authors have made a substantial effort and included new data to address the comments made by this reviewer. I believe that the current manuscript becomes suitable for publication.

A: We are very grateful to Reviewer #1 for the positive comments in our revised manuscript. Thanks to the insightful suggestions from the reviewers, we believe that the revised manuscript appended with new data is much more improved.

Reviewer #2 (Remarks to the Author):

In this manuscript, the authors provided additional data and descriptions to clarify most of the issues raised by the referees. At present, the revised manuscript has significantly been improved to be warranted for publication in Nature Communications with one definition change.

Definition point

1. Whether mitophagy is promoted in cells under some specific physiological conditions without both disrupting Fis1 and overexpressing GFP/mCherry/BFP/Myc/Flag-tagged STX17 still remains to be uncertain. The authors should describe "STX17-mediated mitophagy" more carefully like "STX17 overexpression-mediated mitophagy".

A: We sincerely appreciate Reviewer #2 for highlighting that *"the revised manuscript has significantly been improved to be warranted for publication in Nature Communications"*. Additionally, we would also like to thank the reviewer very much for kindly pointing out the definition change. Given by the precious advice from this reviewer, in this current revised manuscript, we have amended our description as "STX17-mediated mitophagy" into "STX17 overexpression-mediated mitophagy", for better clarity.

Reviewer #3 (Remarks to the Author):

the authors have addressed my concerns. It has been recently reported that SNX17 involves FUNDC1 and Parkin (EMBO J. 2018 Nov 2). The author may discuss this in the discussion.

A: We are deeply grateful to this reviewer for pointing out that "*the authors have addressed my concerns*." Great thanks to the constructive suggestion from this reviewer. In this current revised manuscript, we have supplemented discussion on the positive role of STX17 in PINK1/Parkin-mediated mitophagy, by assisting the cooperation of FUNDC1 and PGAM5 in the Discussion section (please see page 20).
